# Toward a dynamic model of Gelotophobia: Social support, workplace bullying and stress are connected with diverging trajectories of life and job satisfaction among Gelotophobes

**DOI:** 10.1007/s12144-020-01046-y

**Published:** 2020-09-08

**Authors:** Willibald Ruch, Alexander G. Stahlmann

**Affiliations:** 1 National Centre of Competence in Research LIVES – Overcoming Vulnerability: Life Course Perspectives, Switzerland; University of Zurich, Zurich, Switzerland; 2 Professorship for Personality and Assessment, Department of Psychology, University of Zurich, Binzmuehlestrasse 14/7, CH-8050 Zurich, Switzerland

**Keywords:** Gelotophobia, Fear of being laughed at, Vulnerability, Resilience, Social support, Bullying

## Abstract

Recent theoretical advances have grounded gelotophobia (Greek: *gelos* = laughter, *phobos* = fear) in a dynamic framework of causes, moderating factors, and consequences of the fear of being laughed at. This understanding corresponds to that of *vulnerability* and translates gelotophobia into a distinguishable pattern of lacking resources (i.e., misinterpretation of joy and laughter) that can result in negative consequences (e.g., reduced well-being and performance) if individuals have no access to further resources (e.g., social support) or are exposed to severe stressors (e.g., workplace bullying). Based on the panel data provided by the Swiss National Centre of Competence in Research LIVES (*N =* 2469 across six measurement intervals), this study takes the first step toward empirically testing this model’s assumptions: First, we computed exemplary zero-order correlations and showed that gelotophobia was negatively connected with social support (resource) and life and job satisfaction (consequences) and positively connected with perceived stress, work stress, and workplace bullying (stressors). Second, we used longitudinal cluster analyses (KmL; k-means-longitudinal) and showed that the panel data can be clustered into three stable patterns of life and job satisfaction and that gelotophobia is primarily related to the two clusters marked by lower levels of satisfaction. Third, we computed partial correlations and showed that social support, perceived stress, and work stress (but not workplace bullying) can weaken or completely resolve gelotophobia’s relationships with such diverging trajectories of life and job satisfaction. We concluded that seeing gelotophobia through the lens of vulnerability is useful and that such research warrants further attention using more dedicated, theoretically grounded projects.

**Electronic supplementary material:**

The online version of this article (10.1007/s12144-020-01046-y) contains supplementary material, which is available to authorized users.

The past decade has seen increasingly rapid advances in the research on gelotophobia: the fear of being laughed at and appearing ridiculous to social partners (Ruch et al. 2014; Ruch and Proyer 2008). In this process, a previously linear etiological model gradually evolved into a dynamic framework of individual differences, which acknowledges reciprocal effects between putative causes, moderating factors, and consequences of the fear of being laughed at (see Ruch et al. 2014). This framework translates gelotophobia into a process akin to vulnerability: A weakening state of reduced functionality in the face of diminishing personal resources and overwhelming stressors (Spini et al. 2013, 2017). Such a dynamic understanding necessitates the use of longitudinal studies to advance our knowledge of the factors that constitute and sustain gelotophobia (see Platt and Ruch 2010; Proyer et al. 2012a; Ruch and Proyer 2009). Notably, such research can also contribute to identifying factors that enable some gelotophobes to overcome their vulnerability and hence inform educated policies and training programs for more susceptible individuals. In this study, we will take the first step toward applying this dynamic understanding by exploring such factors within the panel data offered by the Swiss National Centre of Competence in Research LIVES – Overcoming vulnerability: Life course perspectives (NCCR LIVES: https://www.lives-nccr.ch/en). We hope that our account can serve as both an example and a catalyst in animating more such dedicated research into the dynamics of gelotophobia.

## Gelotophobia and its Consequences

Cross-sectional and retrospective studies have repeatedly shown that gelotophobes misinterpret genuine joy and laughter as mean-spirited ridicule (Platt 2008; Platt et al. 2013; Ruch et al. 2009a) and report having been bullied more frequently and more severely than non-gelotophobes (Edwards et al. 2010; Platt et al. 2009; Proyer et al. 2009). They typically identify themselves as being shy, inhibited, and insecure (Ruch et al. 2013), and accordingly as rather introverted, emotionally unstable, and low in openness to experience (Proyer et al. 2008; Ruch et al. 2013; Ruch and Proyer 2009). Moreover, they frequently lack confidence in themselves and their capabilities (e.g., emotion regulation, virtuousness, intelligence, and humor production; Papousek et al. 2009; Proyer and Ruch 2009a, 2009b; Ruch et al. 2009b) and report experiencing less social support and more social withdrawal (Platt et al. 2012; Platt et al. 2010; Weibel and Proyer 2012). Accordingly, gelotophobia has been shown to sustain medium to large negative relationships with various indicators of well-being, such as relationship satisfaction, job satisfaction, and overall life satisfaction (Brauer and Proyer 2018; Hofmann et al. 2017; Proyer et al. 2012c; Weibel and Proyer 2012). Gelotophobia can hence be assumed to pertain to several negative consequences in various life domains: In school, relationships, such as with family and friends, and the workplace.

Particularly in the workplace, gelotophobia can be assumed to account for severe negative consequences that also extend to potential damage to the employer or the larger society: Gelotophobia has been associated with dysfunctional behavior and inadequate coping strategies, such as withdrawing from social interactions (e.g., with customers, colleagues, and supervisors), selective absenteeism, and even quitting the job (Führ 2010; Hofmann et al. 2017). There is also emerging evidence that gelotophobia correlates strongly with the Imposter Phenomenon (Brauer and Proyer 2019)–the failure to internalize performance-related success and constant fear of being “exposed” as intellectual fraud–which in turn negatively relates to employees’ salary, organizational citizenship behavior, and organizational commitment (Neureiter and Traut-Mattausch 2016). Moreover, several authors have called for more research and awareness regarding the interaction of gelotophobia with workplace bullying (Platt et al. 2016; Platt et al. 2009; Hofmann et al. 2017): First, gelotophobes may make easy targets for bullies and respond more severely than other coworkers with diminished well-being and performance. Second, gelotophobes might misinterpret joyful teasing among colleagues as mean-spirited ridicule and raise “false alarms”. Especially the latter outcome would be likely to negatively affect the organizational climate, which might lead to spreading concern, dissatisfaction, and distrust, and hence to gelotophobes eventually feeling their preliminary suspicions validated.

## Toward a Dynamic Model of Gelotophobia

Gelotophobia’s interaction with workplace bullying highlights that it is best understood as a dynamic process, which is shaped by mutually dependent relationships between causes, moderators, and consequences of the fear of being laughed at. This dynamic understanding is recognized in Ruch et al.’ (2014) revised model of gelotophobia, which translates it into a process akin to vulnerability: “a lack of resources, which (…) places individuals (…) at a major risk of experiencing (1) negative consequences related to sources of stress; (2) the inability to cope effectively with stressors; and (3) the inability to recover from the stressor or to take advantage of opportunities by a given deadline” (Spini et al. 2013, p. 19). This definition of vulnerability is grounded in Pearlin et al.'s (1981) and Pearlin's (1989) stress process model, which similarly distinguishes between causes, mediators, and outcomes of stress. Key to the framework is the balance of personal resources and emerging stressors–in this language, these are the constituents and correlates mentioned before, such as the ability to correctly interpret genuine joy and laughter, self-confidence, and social support (resources) and demanding social interactions, workplace stress, and workplace bullying (stressors): On the one hand, a lack of essential resources will impede gelotophobes’ coping with daily hassles that would cause either negligible or no trouble for non-gelotophobes. On the other hand, although critical life events and prolonged episodes of fragilization would certainly strain most individuals, gelotophobes will presumably meet with worse consequences than non-gelotophobes (see Spini et al. 2017). Within this framework, we can hence define *gelotophobia* as a distinguishable pattern of lacking resources (i.e., misinterpretation of joy and laughter) that can result in negative consequences (e.g., reduced well-being and performance) if individuals have no access to further resources (e.g., social support) or are exposed to severe stressors (e.g., workplace bullying). We can further refer to *gelotophobes* as individuals who typically display this distinguishable pattern and frequently experience negative consequences due to their vulnerability.

Framing gelotophobia in the language of vulnerability has two main advantages: First, it connects the fear of being laughed at with a model that can be understood and used by researchers from various disciplines other than psychology, including sociology, gerontology, and demography (see Hanappi et al. 2015)–a necessary step to contend with the complexity of the phenomenon. Second, it highlights that individual differences in the access to further resources and exposure to stressors are important determinants of whether and to which degree gelotophobia pertains to negative consequences. This offers a new and interesting perspective on the fear of being laughed at: Although the previously mentioned literature sustains the notion that gelotophobia is generally associated with several negative consequences, we can also assume that there are specific individual differences that allow some gelotophobes to adequately cope with stressors, recover more quickly and soundly, and thus overcome their vulnerability or prevent its development (see Spini et al. 2013). Framed in the revised model of gelotophobia (Ruch et al. 2014), these individual differences correspond to moderators of the fear of being laughed at. Candidates for such individual differences are the correlates that have been identified in the cross-sectional and retrospective studies, but longitudinal research is needed to explore how they can influence gelotophobia’s dynamic relationship with negative consequences (see Ruch and Proyer 2009; Platt and Ruch 2010; Proyer et al. 2012b; Weibel and Proyer 2012). In the end, these individual differences can best inform how we should train more susceptible gelotophobes or design our workplaces to produce more satisfied and successful individuals.

Spini et al. (2017) advocated three analytical directions to guide such dynamic research comprising a multidimensional, a multilevel, and a multidirectional axis: Multidimensional research focuses on interdependencies and spillover effects between resources and stressors *across* different life domains (e.g., family, the workplace, and health). For example, Leitner and Durup (1996) have shown that family personal conflicts can affect perceived work overload and work interference with family. Thus, it can be assumed that domain-specific resources (e.g., social support from the family) may also account for positive spillover effects into other life domains (e.g., job satisfaction). Multilevel research takes into account the diffusion of effects across various layers of psycho-social organization *within* a given domain (e.g., individual, peer-group, and society). Finally, Multidirectional research breaks down individual differences into the dynamics of dispositional and biographical factors *over* time. Spini et al. (2017) recommend that comprehensive research programs aimed at investigating the systemic and dynamic properties of vulnerability are framed along these three major perspectives.

One such research program is the project IP7 Career Paths, which is embedded in the Swiss National Centre of Competence in Research LIVES – Overcoming vulnerability: Life course perspectives (NCCR LIVES: https://www.lives-nccr.ch/en). The project is aimed at relating diverging life and career paths among Swiss adults to individual differences in the access to resources and the exposure to stressors (see Maggiori et al. 2016). Based on longitudinal panel design, the project employs a variety of measures, including assessments of gelotophobia, social support (resource), perceived stress, work stress, and workplace bullying (stressors), and life and job satisfaction (consequences). However, it is important to note that the project has not been explicitly created to include the causes, moderators, and consequences of the fear of being laughed at that Ruch et al. (2014) have compiled. Research using this panel data can hence only be considered a first step toward investigating gelotophobia within a multidimensional and multidirectional framework–We hope that future research can build on our foundation and devise more theoretically grounded studies that are true to the revised model of gelotophobia (Ruch et al. 2014).

## Aims of this Study

Based on the panel data provided by the project IP7, this study seeks to serve as an example on how to identify resources and stressors that distinguish diverging trajectories in life and job satisfaction among gelotophobes and non-gelotophobes. Assuming a multidimensional and multidirectional perspective, we will adopt a three-step procedure to guide data analyses: First, we will examine the relationships of gelotophobia with social support (resource), perceived stress, work stress, and workplace bullying (stressors), and life and job satisfaction (consequences) across IP7’s multiple measurements. Second, we will explore whether trajectories of life and job satisfaction can be clustered into distinct groups and whether an individual’s cluster affiliation will map onto their gelotophobia. Third, we will build on the cluster analysis’ results and investigate whether social support, perceived stress, work stress, and workplace bullying moderate the relationships of gelotophobia with such diverging trajectories of life and job satisfaction. These analyses allow for investigating the potential spillover effect of social support into life- and job-related consequences: We expect that social support generally relates to increased life and job satisfaction. Conversely, we expect that perceived stress, work stress, and workplace bullying relate to decreased life and job satisfaction.

## Method

### Sampling and Procedure

The panel data is representative of the working-age population (i.e., aged 25–55 years) in Switzerland, and traces French- and German-speaking inhabitants’ vocational trajectories over seven consecutive years. Participants were initially selected based on the Swiss national register of inhabitants and approached via invitational letter. At the ensuing measurement intervals (referred to as *waves*), the data was collected using online, paper-pencil, and telephone protocols in both French and German. For this purpose, all the instruments that had not been translated into French and German before the onset of data collection were adapted in collaboration with the original authors. At each wave, job-related stressors and consequences (i.e., work stress, workplace bullying, and job satisfaction) were assessed only for employed participants. The overall characteristics of the corpus, the initial sampling strategy, and the data collection process have been described in detail by Maggiori et al. (2016).

In the study at hand, we tapped into the preliminary corpus comprising the first 6 years of data collection. The panel data will incrementally be made available to the public realm in collaboration with the Swiss Centre of Expertise in the Social Sciences (see https://forsbase.unil.ch/project/study-public-overview/14369/0/). The descriptive statistics of all study variables are provided in the supplementary material to this publication.

### Sample Statistics

Overall, *N* = 2469 participants provided complete data at the first wave in 2012. At this wave, the mean age of this sample was *M* = 41.92 years (*SD* = 8.64 years, *Mdn* = 43 years). Approximately half of the participants identified as female (50.91%) and the other half as male (49.09%). Most of the participants were Swiss citizens (78.98%), while the remainder mainly comprised German (5.51%), French and Portuguese (1.74% each), and Italian (1.62%) citizens. Approximately one third of the participants had been enrolled in tertiary education programs (33.45%), about half of the participants had received upper secondary or post-secondary education (50.68%), few received only lower secondary or less education (7.74%), and the remainder either received a different, non-specified form of education or chose not to provide further information (8.16%). Finally, more than three quarters of the participants reported being (self-)employed (77.52%), a large share of the remainder reported being currently searching for work (21.83%), and few reported being not searching for work (0.65%).

Over the course of data collection, the sample size dropped to *n*_Wave 2_ = 1944, *n*_Wave 3_ = 1629, *n*_Wave 4_ = 1535, *n*_Wave 5_ = 1406, and *n*_Wave 6_ = 1239 participants. This dropout rate was in line with the expectations and was not correlated with any of the demographics mentioned above nor with gelotophobia.

### Instruments

Gelotophobia was assessed by the respective subscale of the *PhoPhiKat-9* (Hofmann et al. 2017). The subscale consists of three items reflecting the facets acknowledged by Platt et al. (2012): (1) coping with derision, (2) disproportionate negative responses to being laughed at, and (3) paranoid sensitivity to anticipated ridicule. A sample item is “When strangers laugh in my presence, I often relate it to me personally” (paranoid sensitivity). The PhoPhiKat-9 uses a four-point scaling (from 1 *= strongly disagree* to 4 *= strongly agree*) and was shown to yield acceptable psychometric properties with Cronbach’s alpha of the gelotophobia subscale ranging between .64 ≤ α ≤ .70 and corrected item-total correlations generally exceeding *r* = .30 (Hofmann et al. 2017). Within IP7, the PhoPhiKat-9 was only employed at waves one and five in 2012 and 2016. Cronbach’s alpha was .65 at both waves, and the scores correlated with *r* = .60. We aggregated both measurements contemporaneously (i.e., across waves) to obtain a general, comprehensive estimate of the participants’ inclination toward gelotophobia.

Life satisfaction was assessed by the *Satisfaction with Life Scale* (SWLS; Diener et al. 1985). The scale consists of five items (e.g., “I am satisfied with my life”) and uses a seven-point scaling (ranging from 1 *= strongly disagree* to 7 *= strongly agree*). Within IP7, it yielded reliable psychometric statistics with Cronbach’s alpha generally exceeding α = .90 across all waves. Job satisfaction was assessed by an adapted version of the *Minnesota Satisfaction Questionnaire* (MSQ; for an extensive description of the instrument see Massoudi 2009). The scale consists of one item depicting overall job satisfaction (i.e., “How satisfied with your job are you as a whole?”) and five items addressing specific work-related aspects (i.e., satisfaction with the supervisor, salary, colleagues, job characteristics, and job security). The items use a four-point answer format (ranging from 1 *= not satisfied* at all to 4 *= very satisfied*). We regarded a compound score comprising the average of the six items as an estimate of the general satisfaction in the workplace. The compound score was corroborated by satisfactory psychometric statistics with Cronbach’s alpha generally exceeding α = .70 across all waves.

Two instruments assessed perceived stress: The *Perceived Stress Scale* (PSS; Cohen et al. 1983) and the *General Work Stress Scale* (GWSS; de Bruin 2006; de Bruin and Taylor 2005). The PSS aims at assessing perceived stress in life as a whole through five items (e.g., “How often have you felt nervous and stressed?”) and uses a five-point answer format (ranging from 1 *= never* to 5 *= very often*). The GWSS focuses specifically on stress perceived in the workplace and consists of nine items (e.g., “Do you feel that you are unable to manage your work?”) and also uses a five-point scaling (ranging from 1 *= never* to 5 *= always*). Within IP7, both instruments yielded reliable psychometric properties with Cronbach’s alpha generally exceeding α = .75 (PSS) and α = .85 (GWSS) across all waves.

Social support was assessed by the *Functional Social Support Questionnaire* (Duke-UNC; Broadhead et al. 1988). The instrument captures the quantity of social support that a person experiences in two dimensions: Affective support (e.g., “I have people who care about what happens to me”) and confidant support (e.g., “I have chances to talk to someone I trust about my personal and family problems”). It consists of eight items and uses a five-point scaling (ranging from 1 *= much less than I would like* to 5 *= as much as I would like*). We aggregated all items into a compound score to obtain an overall estimate of the participants’ experienced social support. Within IP7, the score was substantiated by Cronbach’s alpha generally surpassing α = .90 across all waves.

Workplace bullying was assessed by the *Workplace Incivility Scale* (WIS; Cortina et al. 2001). The instrument consists of four items and uses a five-point scaling (ranging from 1 *= never* to 5 *= very often*) A sample item is “During the past year / During your last job, how frequently did a boss or a co-worker of yours humiliate you or condescend to you?”. Within IP7, the WIS yielded reliable psychometric properties with Cronbach’s alpha generally exceeding α = .85 across all waves. Notably, participants generally reported being only little subject to workplace bullying with *M* < 1.78, Skewness *>*1.45, and Kurtosis >1.75 (i.e., leptokurtic floor effect).

### Data Analysis

All of the analyses were conducted within the R statistical computing environment (R Core Team 2018). First, we examined zero-order correlations of gelotophobia with social support (resource), perceived stress, work stress, workplace bullying (stressors), and life and job satisfaction (consequences) across all waves. Second, we conducted longitudinal cluster analyses to explore multidirectional trajectories of life and job satisfaction and their relationships with gelotophobia. Finally, we examined partial correlations to investigate whether social support, perceived stress, work stress, and workplace bullying moderate the relationships of gelotophobia with such diverging trajectories of life and job satisfaction. We adjusted the confidence intervals of all correlation coefficients reported in this publication via Holm’s correction using the R package *psych* (Revelle 2018).

### Cluster Analyses

We conducted the longitudinal cluster analyses using the modified k-means clustering algorithm *KmL* (Genolini et al. 2015; Genolini and Falissard 2010). In general, k-means aims at assigning each case to a suitable cluster by iteratively minimizing the mean deviation between cases and their respective centroids (i.e., cluster centers). As a result, cases summarized in the same cluster can be considered more similar to each other than cases assigned to different clusters (for an in-depth description and evaluation of the k-means algorithm see Jain 2010). The modified algorithm KmL extends this notion to longitudinal data and allows for identifying prevailing patterns within the *trajectories* of life and job satisfaction. Although KmL also allows for simultaneously evaluating multivariate trajectories (e.g., joint-trajectories of life and job satisfaction; see Genolini et al. 2015), we decided to evaluate both well-being measures independently of each other to account for potentially distinct relational patterns with gelotophobia.

To evaluate the resulting solutions, we compared the clusters regarding consistency (i.e., homogeneity within clusters), distinctiveness (i.e., degree of separation between clusters), directionality (i.e., flatness or trend) and criterion validity (i.e., relationship with gelotophobia). We evaluated consistency and distinctiveness simultaneously using the Caliński and Harabasz criterion *C*(g). This criterion has been repeatedly shown to be one of the most reliable validity indices (Milligan and Cooper 1985; Shim et al. 2005) within the framework of cluster analysis. Similar to the *F*-test statistic, the between-cluster-variance is divided by the within-cluster-variance, thus resulting in higher values corroborating the validity of the respective clustering solution (Caliński and Harabasz 1974; Genolini and Falissard 2010). By default, KmL runs each k-means algorithm 20 times with slightly varying starting parameters (i.e., randomly selected centroids). Accordingly, we compared the respective Caliński and Harabasz criteria across 20 partitions, each.

After examining the validity indices, we investigated distinctiveness and directionality via linear mixed-effects modeling (LMM) using the R package *lme4* (Bates et al. 2015): The models comprised either life or job satisfaction as criteria, the respective wave and cluster affiliation as fixed effects, and between-subjects variation within each cluster as a random effect. Significant effects were examined post hoc via Tukey contrasts. Finally, we evaluated the criterion validity of the clustering solutions via point-biserial correlations with gelotophobia.

## Results

### Zero-Order Correlation Analyses

The zero-order correlations of gelotophobia with social support, perceived stress, work stress, workplace bullying, and life and job satisfaction across all waves are depicted in Table 1. The pattern reflected our expectations with life satisfaction, job satisfaction, and social support sustaining negative relationships with gelotophobia while perceived stress, work stress, and workplace incivility correlated positively with gelotophobia. The effect sizes ranged from small effects (i.e., job satisfaction, workplace incivility) to small to medium effects (i.e., life satisfaction, social support, perceived stress, work stress). Our findings suggest that gelotophobia was consistently related to negative consequences, lacking resources, and stressors across all six waves.

**Table 1:** Zero-order correlations of gelotophobia with social support (resource), perceived stress, work stress, and workplace bullying (stressors), and life and job satisfaction (consequences) as a function of the respective wave

Correlate	Wave 1	Wave 2	Wave 3	Wave 4	Wave 5	Wave 6
Social support	−.26 [−.29 | −.22] *n* = 2440	−.21 [−.26 | −.17] *n* = 1651	−.21 [−.27 | −.16] *n* = 1280	−.26 [−.31 | −.21] *n* = 1279	−.30 [−.35 | −.24] *n* = 1105	−.29 [−.34 | −.22] *n* = 895
Perceived stress	.34 [.31 | .38] *n* = 2444	.30 [.25 | .34] *n* = 1650	.30 [.25 | .35] *n* = 1279	*n* = 0	.36 [.31 | .41] *n* = 1103	.34 [.28 | .39] *n* = 895
Work stress	.24 [.20 | .28] *n* = 1882	.24 [.19 | .29] *n* = 1420	.25 [.20 | .30] *n* = 1161	.25 [.20 | .30] *n* = 1149	.26 [.20 | .32] *n* = 989	.21 [.15 | .28] *n* = 788
Workplace bullying	.11 [.07 | .15] *n* = 2419	.14 [.09 | .18] *n* = 1607	.14 [.09 | .20] *n* = 1294	.15 [.09 | .20] *n* = 1268	.18 [.12 | .24] *n* = 1103	.13 [.07 | .20] *n* = 873
Life satisfaction	−.21 [−.25 | −.18] *n* = 2444	−.20 [−.24 | −.15] *n* = 1648	−.21 [−.26 | −.15] *n* = 1274	−.26 [−.31 | −.21] *n* = 1279	−.25 [−.31 | −.20] *n* = 1101	−.24 [−.30 | −.18] *n* = 893
Job satisfaction	−.15 [−.19 | −.11] *n* = 1882	−.16 [−.21 | −.11] *n* = 1507	−.17 [−.22 | −.11] *n* = 1231	−.16 [−.21 | −.10] *n* = 1211	−.14 [−.20 | −.08] *n* = 1040	−.11 [−.18 | −.04] *n* = 829

Each cell contains the correlation coefficient, the respective 95% confidence limits (adjusted via Holm’s correction), and the sample size. Perceived stress had not been measured at Wave 4

### Multidirectional Trajectories of Life and Job Satisfaction and their Relationships with Gelotophobia

Overall, *n*_*LS*_ = 1902 (life satisfaction) and *n*_JS_ = 1769 (job satisfaction) participants provided enough data to be considered eligible for the clustering algorithm KmL. We partitioned the data into two, three, four, and five clusters for life and job satisfaction and began with evaluating both consistency and distinctiveness of the resulting clustering solutions by examining the Caliński and Harabasz criteria. Regarding life satisfaction, the criteria were estimated at *C*_LS_(2) ≥ 2491.66, *C*_LS_(3) ≥ 2953.57, *C*_LS_(4) ≥ 3007.45, and *C*_LS_(5) ≥ 2731.93. Regarding job satisfaction, the criteria were estimated at *C*_JS_(2) ≥ 1187.04, *C*_JS_(3) ≥ 1331.15, *C*_JS_(4) ≥ 1257.73, and *C*_JS_(5) ≥ 1204.42. Taken together, the Caliński and Harabasz criteria suggested that either three-cluster or four-cluster solutions would best fit the trajectories of life and job satisfaction.

**Fig. 1:**
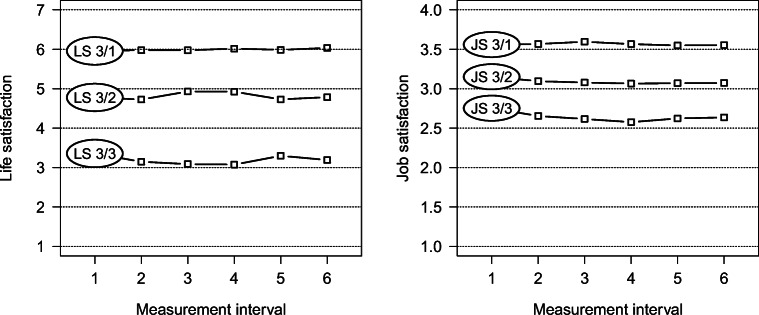
Temporally aggregated trajectories (i.e., across participants) of the three-cluster-solutions for life and job satisfaction, respectively

Next, we evaluated the distinctiveness and directionality of every clustering solution by examining the results of the LMM. The overall model estimates–expressed in the ANOVA framework–are depicted in Table 2. In general, almost all of the effects evaluated within the models achieved statistical significance. However, the cluster affiliation clearly accounted for the largest effect sizes with the respective *F*-statistics vastly outweighing the other estimates. Indeed, ensuing post hoc analyses sustained the notion that the various clusters primarily tell apart different levels of rather mean stationary trajectories (the particular estimates of the planned contrasts are provided in the supplementary material to this publication). Independent of the respective clustering solution, the trajectories can hence be considered largely flat and parallel to each other. This is exemplified in Fig. 1, which depicts the temporally aggregated trajectories (i.e., across participants) of both three-cluster solutions.

**Table 2:** Results of the LMM depicted as ANOVA F-statistics

Cluster solution	Wave	Cluster affiliation	Interaction effect
LS 2	*F*(5, 6179) = 1.90	*F*(1, 1900) = 4305.80 ***	*F*(5, 6179) = 9.30 ***
JS 2	*F*(5, 5565) = 8.00 ***	*F*(1, 1767) = 2963.00 ***	*F*(5, 5565) = 3.00 **
LS 3	*F*(5, 6174) = 2.20 *	*F*(2, 1899) = 5021.10 ***	*F*(10, 6174) = 7.60 ***
JS 3	*F*(5, 5560) = 8.00 ***	*F*(2, 1766) = 3256.00 ***	*F*(10, 5560) = 4.00 ***
LS 4	*F*(5, 6169) = 3.00 **	*F*(3, 1898) = 5474.00 ***	*F*(15, 6169) = 6.00 ***
JS 4	*F*(5, 5555) = 9.00 ***	*F*(3, 1765) = 2884.00 ***	*F*(15, 5555) = 17.00 ***
LS 5	*F*(5, 6164) = 3.00 **	*F*(4, 1897) = 5488.00 ***	*F*(20, 6164) = 7.00 ***
JS 5	*F*(5, 5550) = 10.00 ***	*F*(4, 1764) = 2304.00 ***	*F*(20, 5550) = 55.00 ***

‘’ n.s.; * *p* ≤ .050; ** *p* ≤ .010; *** *p* ≤ .001. LS = Life satisfaction. JS = Job satisfaction. The first (upper) statistic in each cell refers to life satisfaction whereas the second (lower) statistic refers to job satisfaction

**Fig. 2:**
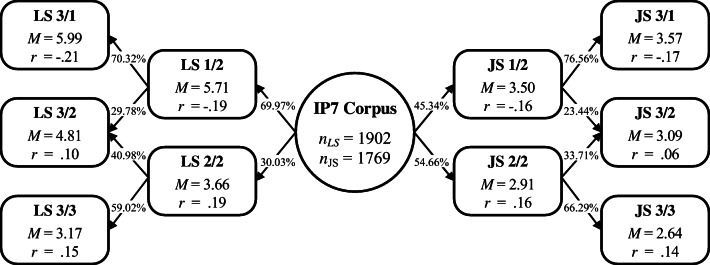
Two-cluster and three-cluster solutions for life satisfaction (left) and job satisfaction (right), including contemporaneous mean levels (i.e., aggregated across measurements) and point-biserial correlations with gelotophobia. All correlation coefficients are significant with *p ≤* .01 (adjusted via Holm’s correction)

Last, we examined the criterion validity of every clustering solution by relating the emerging groups to gelotophobia. Across the various solutions, gelotophobia was negatively correlated with the highest-scoring clusters. Regarding the two-cluster and the three-cluster solutions, gelotophobia was additionally positively correlated with the remaining clusters. The partition of the overall corpus into these two and three clusters, as well as the respective correlations with gelotophobia, are depicted in Fig. 2.

Regarding the four-cluster and the five-cluster solutions, an increasing number of the remaining clusters did not put forward significant correlations with gelotophobia anymore (the particular statistics are provided in the supplementary material to this publication). Although the data can be split into more than three groups, such partitioning would be irrelevant for our understanding of how gelotophobia maps onto diverging trajectories in life and job satisfaction. Accordingly–and in line with our previous findings regarding consistency, distinctiveness, and directionality–we concluded that the three-factor solutions best fit the data. Therefore, we partitioned the trajectories of life and job satisfaction into three mean stationary patterns. We labeled the high-scoring clusters LS/1 and JS/1, the average-scoring clusters LS/2 and JS/2, and the low-scoring clusters LS/3 and JS/3, respectively. The high-scoring clusters are related negatively to gelotophobia, and the two remaining clusters are related positively to gelotophobia.

### Resources and Stressors Moderate the Relationships of Gelotophobia with Multidirectional Trajectories of Life and Job Satisfaction

The cluster analyses suggested that the trajectories of life and job satisfaction in IP7 are primarily shaped by mean stationary patterns–in contrast to patterns of decline, ascent, or variation (e.g., sinuous trends). Accordingly, we cannot expect much variation across waves and predict that the majority of individuals would assess their life and job satisfaction similarly to their previous estimates in hypothetical future measurements. Regarding our analyses into how social support, perceived stress, work stress, and workplace bullying moderate the relationship of gelotophobia with such mean stationary patterns, we hence aggregated all measures contemporaneously (i.e., across waves) and compared partial correlations of the resulting mean scores. The results are depicted in Table 3. In general, our findings show that controlling for resources and stressors significantly weakened the negative relationships of gelotophobia with life and job satisfaction. In particular, the negative relationships with life satisfaction were weakened by margins of .14 (social support) and .07 (work stress), and they were completely resolved when controlling for perceived stress. In turn, the negative relationships with job satisfaction were weakened by margins of .07 (social support) and .11 (perceived stress/work stress). The correlations of gelotophobia with both life and job satisfaction were also completely resolved when simultaneously controlling for social support, perceived stress, work stress, and workplace bullying. However, although workplace bullying numerically weakened the relationships of gelotophobia with life and job satisfaction, this margin was not significant. Taken together, the results suggest that gelotophobia’s negative relationships with life and job satisfaction are moderated by social support and perceived stress (especially concerning life satisfaction) and by work stress (especially concerning job satisfaction).

**Table 3:** Correlations of gelotophobia with life and job satisfaction (consequences): Zero-order correlations (column 1) and partial correlations, controlled for social support (column 2), perceived stress (column 3), work stress (column 4), workplace bullying (column 5), and all of the above (column 6)

Consequence	Zero-order correlation	Con. for social support	Con. for perceived stress	Con. for work stress	Con. for workplace bullying	Con. for all
Life satisfaction	−.23 [−.29 | −.17] *n* = 2526	−.09 [−.14 | −.04] *n* = 2525	.00 [−.05 | .05] *n* = 2525	−.16 [−.21 | −.11] *n* = 2525	−.20 [−.24 | −.15] *n* = 2525	.05 [−.01 | .10] *n* = 2522
Job satisfaction	−.17 [−.23 | −.11] *n* = 2272	−.10 [−.15 | −.05] *n* = 2271	−.06 [−.11 | −.01] *n* = 2271	−.06 [−.11 | −.01] *n* = 2271	−.12 [−.16 | −.07] *n* = 2271	.00 [−.04 |.04] *n* = 2268

Each cell contains the correlation coefficient, the respective 95% confidence limits (adjusted via Holm’s correction), and the sample size. The data has been aggregated contemporaneously (i.e., across measurements) across the six waves of data collection

## Discussion

This account set out to exemplify how a study grounded in a dynamic understanding of gelotophobia can contribute to identifying factors that enable some gelotophobes–individuals who are typically susceptible to vulnerability–to lead satisfactory lives. For this purpose, we tapped into the longitudinal panel data provided by the project IP7 Career Paths and examined whether the access to social support (resource) and the exposure to perceived stress, work stress, and workplace bullying (stressors) map onto diverging trajectories in life and job satisfaction (consequences). Based on preliminary correlational analyses, we found gelotophobia to be negatively associated with life satisfaction, job satisfaction, and social support and positively associated with perceived stress, work stress, and workplace bullying across all measurement intervals. Next, we conducted longitudinal cluster analyses and showed that the panel data can be partitioned into two sets of three clusters that distinguish mean stationary trajectories of life and job satisfaction, respectively. Regarding both well-being measures, gelotophobia was related negatively to the high-scoring clusters and positively to the average-scoring and the low-scoring clusters. Last, we investigated whether the access to resources and the exposure to stressors can moderate the connection of gelotophobia with such mean stationary trajectories of life and job satisfaction, and our results suggest that social support, perceived stress, and work stress (but not workplace bullying) can weaken gelotophobia’s connection with life and job satisfaction. Our findings substantiate the results of previous correlational and retrospective studies in that gelotophobia was connected with less access to resources, more exposure to stressors, and lower levels of well-being. Furthermore, the cluster analyses suggested that these effects are largely stable across time and not much subject to any more change. However, gelotophobia was not unequivocally connected with reduced life and job satisfaction because further individual differences in resources and stressors could weaken or even resolve these negative relationships. Although gelotophobes are certainly susceptible to vulnerability, this differentiation shows that some individuals can overcome their vulnerability–either due to access to more resources or less exposure to stressors. We conclude that it is critical to further explore such resources and stressors, expand our understanding of the factors that empower these gelotophobes to lead satisfactory lives, and learn from them to better support more susceptible individuals.

### Social Support and Stress Discriminate between Gelotophobes Who Lead Satisfactory Lives and those Who Do Not

The results of this research suggest that social support constitutes one such important factor. At first glance, this finding may appear contradictory to the definition of gelotophobia, which entails social withdrawal to avoid being ridiculed (see Platt et al. 2012; Ruch et al. 2014). However, although social withdrawal and low social support can be assumed to be generally related, they are not necessarily equivalent: For example, withdrawal from social interactions in the workplace or at school will presumably result in gelotophobes reporting less social support from their colleagues and peers, but not necessarily from their spouse, family, and close friends. Indeed, in a recent study among young Italian adults, gelotophobes reported resorting to withdrawal *and* searching for social support–presumably from their families and close friends–when confronted with challenging situations (Canestrari et al. 2019). Gelotophobes seem to be generally less likely to engage in romantic relationships (see Brauer et al. 2020; Brauer and Proyer 2020), but there is also evidence that–when they can find a partner–they look for similar patterns in gelotophobia and further dispositions toward ridicule and laughter (Brauer and Proyer 2018; Proyer et al. 2012a). Maybe it is this “safe haven” among people that can mutually understand each other–and the resulting social support–that contributes to building resilience. Specifically, such intimate relationships may provide (a) room for discussion on whether individuals really are being ridiculed or not, (b) reassurance that they are not inherently derisible, and (c) build other resources, such as self-efficacy, when they experience some kind of mastery–for example when raising a mutual child. Notably, this idea strongly opposes the concept of a detrimental “folie à deux”, in which one or both partners would reinforce the other’s paranoia. Instead, our data suggests that most gelotophobes benefit from social support and dependability, and as such, fostering social support (or helping gelotophobes develop more appropriate attachment styles; see Brauer et al. 2020) could be one of the main objectives when working with gelotophobic clients in counseling settings. However, further empirical research into which, how, and with whom gelotophobes maintain supportive relationships is required, preferably assuming a multidimensional perspective.

The results on workplace bullying are more complex: Although it was negatively related to gelotophobia, it did not notably affect gelotophobia’s relationships with life and job satisfaction. Again, this seemingly constitutes a contradiction to the literature, which identified bullying as a potent risk factor for gelotophobes’ well-being and career development (see Hofmann et al. 2017; Platt et al. 2016). However, it is important to recall that reported levels of workplace bullying in the panel data were low and that this apparent floor effect may have skewed the results. Accordingly, our findings may not be as descriptive of gelotophobia as they are descriptive of Switzerland’s working-age population, in which gelotophobes’ well-being seems to hinge more on social support and stress than on bullying. Although this may appear as good news for the majority of gelotophobes living in similarly structured countries, it does not speak to the question of how much damage the interaction of gelotophobia in the face of intense bullying can do, and we reckon that other research designs will be needed to address this issue. For example, it can be assumed that institutional properties and conditions in the workplace affect gelotophobes’ sensitivity and susceptibility toward bullying, and such properties and conditions could be targeted in a dedicated research project. This could involve intervention programs aimed at changing organizational norms regarding face, honor, and shame, strong hierarchies, and status divisions (Davies 2009) but also such that aim at raising awareness of the potentially detrimental aspects of laughter among colleagues (Hofmann et al. 2017; Platt et al. 2016)–ideally in workplaces that have a problem with reported cases of bullying. As the results stand now, the overall burden of workplace bullying on gelotophobes’ well-being can be considered small, but the true costs for employers or the larger society remain unclear. The interaction of gelotophobia and bullying is certainly multifaceted and will be affected by several further factors, and more research into these factors needs to be undertaken–preferably assuming a multilevel perspective.

Stress constitutes the last and most abstract factor identified in this study. Building on Spini et al.' (2013) definition of vulnerability, perceived stress can be considered as a consequence of an imbalance between an individual’s access to resources and their exposure to stressors. Accordingly, stress will presumably be reflected by individual differences in resources such as social support and workplace bullying, but also by further differences that were not explored within this study. Additional resources could include character strengths, which have been repeatedly shown to sustain notable relationships with various positive outcomes, such as life and job satisfaction, mental health, and performance (for an overview, see Harzer 2016; Niemiec 2013). Although gelotophobes typically reported lower endorsement of most character strengths, gelotophobia was shown to be positively related to humility and prudence (Proyer and Ruch 2009a; Proyer et al. 2014)–strengths that protect individuals from arrogance and recklessness (Peterson 2006). It can be assumed that the endorsement of these strengths may enable gelotophobes to partially overcome their vulnerability–for example, by avoiding potentially awkward social interactions or by carefully choosing their careers. Notably, it might be the gelotophobes who turn their weaknesses into their strengths–self-deprecation into humility and prudishness into prudence–that eventually lead satisfactory, successful lives (see Peterson 2006; Proyer et al. 2014). On the other hand, additional stressors could include exposure to the growing uncertainty associated with the shift from industrial to post-industrial societies (Spini et al. 2013). This uncertainty refers, among others, to new social risks, such as family discontinuities, the demand for more flexibility and engagement in the workplace, and the fear of social decline (Spini et al. 2013). The post-industrial risk society “(…) dissolves the traditional parameters of industrial society: class culture and consciousness, gender and family roles” (Beck 1992, p. 87), and individuals are increasingly expected to become self-directed agents of their life courses (Kohli 1986). Accordingly, they are continually pressured to make the right choices without benefitting from the rigid but supportive framework that their grandparents may have experienced (Spini et al. 2013). Although gelotophobes and non-gelotophobes alike will be affected by these societal changes, gelotophobes will presumably have more trouble coping with the uncertainty (see Spini et al. 2017). Certainly, character strengths and new social risks only constitute hypothetical examples. Another example is the Imposter Phenomenon, which–by definition–refers to a constant fear of being exposed as intellectual fraud and has hence been associated with a constant experience of stress and emotional exhaustion (Whitman and Shanine 2012). There is abundant room for further progress in determining additional resources and stressors that may empower gelotophobes to lead satisfactory lives.

### Limitations

Although this study rested upon representative panel data, the results are limited by our methodological approach and the inherent sampling bias of the corpus. First, both longitudinal cluster analyses, as well as correlational analyses, are not suited to investigate causal relationships between resources, stressors, and consequences of vulnerability. Certainly, the longitudinal panel design would have allowed for conducting analyses aimed at evaluating causality, such as cross-lagged panel analyses. However, the strong stability within every cluster suggested that, at this age, most of the differences had already been “ingrained” into the trajectories, and that substantial intra-individual changes cannot be expected, anymore. Accordingly, we concluded that such analyses would not have yielded notable findings that go beyond the within-cluster variation. Certainly, these results do not imply that changes, for example, from the lowest to the higher of the two lower-scoring clusters, are impossible. However, we assume that the foundations for later differences are primarily determined during infancy, childhood, and adolescence. This conclusion further stresses the importance of life-course studies and early interventions, such as in school (see Platt et al. 2016).

Second, the panel data was based on the working-age population rather than on clinically diagnosed gelotophobes, and it can be assumed that gelotophobia affects their lives less severely. Indeed, the literature suggests that most of the more severe consequences of gelotophobia, such as paranoid tendencies, intense physiological responses, and social withdrawal primarily prevail among individuals with marked and extreme fear of being laughed at (Platt et al. 2012; Ruch 2009). Therefore, it is unclear whether the results of this study also extend to those more severely affected individuals. On the other hand, the relative absence of individuals with extreme gelotophobia (and hence paranoid tendencies) lends further credibility to the self-report data of the corpus, such as the assessment of workplace bullying. Moreover, the majority of gelotophobes typically report only slight fear of being laughed at (see Platt and Forabosco 2012). Although the results of this study may not represent all gelotophobes, they presumably depict a snapshot of the majority.

Third, despite being representative of Switzerland, it is unclear to what extent the implications of this study generalize to gelotophobes living and working in other countries and cultural groups. Green et al. (2005) showed that Switzerland can be considered a largely non-competitive, self-reliant nation–similar to most other Western European countries and Singapore (i.e., horizontal individualism; see Györkös et al. 2013; Singelis et al. 1995). Such nations are certainly individualistic and thus emphasize autonomy, self-direction, and hedonism (Green et al. 2005). However, competitive value orientations are typically replaced by orientations toward individual freedom, personal development, quality of life, and relational interdependence (Basabe and Ros 2005). Beyond Switzerland, it can be assumed that gelotophobes who live in more competitive nations will more often be left behind by their peers, employers, and welfare programs. This will presumably be reflected by a more distinct separation between few gelotophobes who get ahead and the outpaced remainder. In collectivist nations, laughter is typically considered a disgrace for both individuals as well as their families, corporate organizations, and classmates (Davies 2009). Consequently, gelotophobes are likely to fear laughter, both directed at themselves and their in-groups. In a comparison between one such collectivist nation–Mainland China–and Switzerland, the prevalence of gelotophobia was largely comparable between both countries (Proyer et al. 2012c). However, the fear of being laughed at weighted heavier on Chinese gelotophobes orientation toward pleasure-seeking and perceived meaning in life. Certainly, further empiric studies are required that explore individual differences among gelotophobes in competitive and collectivist nations.

Fourth, although this study investigated gelotophobia as a focal variable, the results may not be specific to the fear of being laughed at. Indeed, gelotophobia shares some overlap with similar phenomena, such as shame-proneness and social anxiety (see Carretero-Dios et al. 2010; Proyer et al. 2010), and it cannot be ruled out that it is this common factor that accounts for the differences in social support, perceived bullying, stress, and well-being. This raises the question of whether there are qualitative differences–whether gelotophobes garner social support differently than individuals who are subject to social anxiety, or whether they deal differently with bullying. The results of such research will decide whether more specific interventions are needed or whether broader interventions (that can also benefit individuals who are prone to vulnerability due to other yet similar reasons) should be advanced.

Finally, as the longitudinal panel design required the participants to subject themselves to long interviews and questionnaires over 6 years, it can be assumed that attrition was not randomly distributed. Instead, it was presumably shaped by several further individual differences, such as regarding personality, health, and job characteristics (for an overview of variables that commonly co-occur with attrition see, Chatfield et al. 2005; Young et al. 2006). Thus, it is unclear whether the findings might be subject to a certain degree of deflation (i.e., more variation would lead to bigger differences) or inflation (i.e., the results only concern a selected group).

### Conclusion

On the whole, this study offers first insights into resources and stressors that enable gelotophobes to overcome vulnerability. Our results suggest that social support, workplace bullying, and stress are differentially related to diverging life and career paths both between gelotophobes and non-gelotophobes and among gelotophobes specifically. However, this list is hardly exhaustive, and neither are the entries’ mechanisms well-explained. What is now needed is more focused and theoretically grounded research into these and further potential resources and stressors. Specifically, presumable resources and stressors should be evaluated concerning three critical questions: (1) “is the factor reflected by diverging life and career paths?”, (2) “does it causally account for these differences?”, and (3) “how exactly does it influence cognition, affect, and behavior?”. By assuming a multidimensional, multilevel, or multidirectional perspective, Spini et al.' (2017) dynamic life-course framework is well-suited to address these questions. Learning from gelotophobes who already built resilience will allow us to devise more beneficial policies and training programs, and hence to eventually build frameworks and conditions that also enable more susceptible gelotophobes to lead satisfactory, successful lives.

## Electronic supplementary material

ESM 1 (DOCX 58 kb)

ESM 2 (HTML 1.54 mb)
