# Supplementary material for: Toward a dynamic model of Gelotophobia: Social support, workplace bullying and stress are connected with diverging trajectories of life and job satisfaction among Gelotophobes
Source: Curr Psychol. 2020 Sep 8;42(19):16368–80. doi: 10.1007/s12144-020-01046-y (PMC10404568; doi:10.1007/s12144-020-01046-y)
Supplement: Supplementary file 1 — (DOCX 58 kb) [file 12144_2020_1046_MOESM1_ESM.docx]

## Supplementary material to the article “Toward a Dynamic Model of Gelotophobia: Social Support, Workplace Bullying and Stress Are Connected with Diverging Trajectories of Life and Job Satisfaction Among Gelotophobes”

**Tables 1-7** contain the descriptive statistics of the study instruments–PhoPhiKat-9: Gelotophobia; Satisfaction with Life Scale: Life satisfaction; Minnesota Satisfaction Questionnaire: Job satisfaction, Perceived Stress Scale: Perceived stress, General Work Stress Scale: Work stress, Functional Social Support Questionnaire: Social support, and Workplace Incivility Scale: Workplace bullying. The tables include sample size (n), mean (M), standard deviation (SD), median (Mdn), minimum (Min), maximum (Max), skewness (Skew), and kurtosis (Kurt) respectively.

**Tables 1-15** contain the planned contrasts evaluated within the LMM. All planned contrasts are orthogonal. The pipe operator indicates comparisons between the respective contemporaneously aggregated cluster solutions (distinctiveness). For example, C1|C2 indicates the comparison between clusters one and two. The colon operator indicates comparisons between a certain wave and all other waves within a given cluster (directionality/flatness). For example, C1: W1 indicates the comparison between wave one and waves two to six within cluster one.
Est.: estimated slope; SE: respective standard error; z: corresponding z-score;
Sig: ‘ ’ n.s.; * *p* ≤ .050; ** *p* ≤ .010; *** *p* ≤ .001.

**Table 16** contains the point-biserial correlations of gelotophobia with all clusters suggested by the respective solutions. Each cell contains the correlation coefficient and the respective 95% confidence limits (adjusted via Holm’s correction).

Table 1

*Descriptive statistics of the PhoPhiKat-9: Gelotophobia subscale.*

|  | n | M | SD | Mdn | Min | Max | Skew | Kurt |
| --- | --- | --- | --- | --- | --- | --- | --- | --- |
| Wave 1 | 2447 | 1.92 | 0.65 | 2.00 | 1.00 | 4.00 | 0.52 | -0.13 |
| Wave 2 |  |  |  |  |  |  |  |  |
| Wave 3 |  |  |  |  |  |  |  |  |
| Wave 4 |  |  |  |  |  |  |  |  |
| Wave 5 | 1112 | 1.91 | 0.65 | 1.67 | 1.00 | 4.00 | 0.58 | -0.04 |
| Wave 6 |  |  |  |  |  |  |  |  |

Table 2

*Descriptive statistics of the Satisfaction with Life Scale: Life satisfaction.*

|  | n | M | SD | Mdn | Min | Max | Skew | Kurt |
| --- | --- | --- | --- | --- | --- | --- | --- | --- |
| Wave 1 | 2448 | 5.03 | 1.27 | 5.40 | 1.00 | 7.00 | -0.90 | 0.15 |
| Wave 2 | 1751 | 5.08 | 1.26 | 5.40 | 1.00 | 7.00 | -1.00 | 0.46 |
| Wave 3 | 1320 | 5.12 | 1.25 | 5.40 | 1.00 | 7.00 | -1.00 | 0.47 |
| Wave 4 | 1320 | 5.17 | 1.29 | 5.60 | 1.00 | 7.00 | -1.10 | 0.56 |
| Wave 5 | 1102 | 5.14 | 1.22 | 5.60 | 1.00 | 7.00 | -1.00 | 0.47 |
| Wave 6 | 904 | 5.16 | 1.29 | 5.60 | 1.00 | 7.00 | -0.90 | 0.21 |

Table 3

*Descriptive statistics of the Minnesota Satisfaction Questionnaire: Job satisfaction.*

|  | n | M | SD | Mdn | Min | Max | Skew | Kurt |
| --- | --- | --- | --- | --- | --- | --- | --- | --- |
| Wave 1 | 1895 | 3.21 | 0.44 | 3.17 | 1.00 | 4.00 | -0.20 | 0.13 |
| Wave 2 | 1633 | 3.18 | 0.47 | 3.17 | 1.00 | 4.00 | -0.50 | 0.76 |
| Wave 3 | 1306 | 3.17 | 0.46 | 3.17 | 1.20 | 4.00 | -0.30 | 0.28 |
| Wave 4 | 1267 | 3.15 | 0.48 | 3.17 | 1.00 | 4.00 | -0.40 | 0.61 |
| Wave 5 | 1069 | 3.16 | 0.46 | 3.17 | 1.20 | 4.00 | -0.40 | 0.52 |
| Wave 6 | 855 | 3.17 | 0.46 | 3.17 | 1.30 | 4.00 | -0.30 | 0.62 |

Table 4

*Descriptive statistics of the Perceived Stress Scale: Perceived stress.*

|  | n | M | SD | Mdn | Min | Max | Skew | Kurt |
| --- | --- | --- | --- | --- | --- | --- | --- | --- |
| Wave 1 | 2445 | 2.39 | 0.66 | 2.4 | 1.00 | 5.00 | 0.37 | 0.29 |
| Wave 2 | 1753 | 2.38 | 0.65 | 2.4 | 1.00 | 5.00 | 0.45 | 0.17 |
| Wave 3 | 1325 | 2.41 | 0.67 | 2.4 | 1.00 | 5.00 | 0.51 | 0.39 |
| Wave 4 |  |  |  |  |  |  |  |  |
| Wave 5 | 1104 | 2.34 | 0.65 | 2.4 | 1.00 | 5.00 | 0.44 | 0.34 |
| Wave 6 | 906 | 2.36 | 0.67 | 2.4 | 1.00 | 4.60 | 0.39 | 0.04 |

Table 5

*Descriptive statistics of the General Work Stress Scale: Work stress.*

|  | n | M | SD | Mdn | Min | Max | Skew | Kurt |
| --- | --- | --- | --- | --- | --- | --- | --- | --- |
| Wave 1 | 1891 | 1.87 | 0.58 | 1.78 | 1.00 | 4.60 | 0.74 | 0.56 |
| Wave 2 | 1507 | 1.90 | 0.62 | 1.78 | 1.00 | 5.00 | 0.82 | 0.88 |
| Wave 3 | 1201 | 1.88 | 0.65 | 1.78 | 1.00 | 5.00 | 0.95 | 1.00 |
| Wave 4 | 1183 | 1.85 | 0.65 | 1.78 | 1.00 | 4.90 | 0.99 | 1.20 |
| Wave 5 | 992 | 1.88 | 0.63 | 1.78 | 1.00 | 5.00 | 0.85 | 0.80 |
| Wave 6 | 801 | 1.89 | 0.67 | 1.78 | 1.00 | 5.00 | 0.97 | 1.25 |

Table 6

*Descriptive statistics of the Functional Social Support Questionnaire: Social support.*

|  | n | M | SD | Mdn | Min | Max | Skew | Kurt |
| --- | --- | --- | --- | --- | --- | --- | --- | --- |
| Wave 1 | 2442 | 4.13 | 0.83 | 4.25 | 1.00 | 5.00 | -1.20 | 1.10 |
| Wave 2 | 1756 | 4.15 | 0.84 | 4.38 | 1.00 | 5.00 | -1.20 | 1.22 |
| Wave 3 | 1326 | 4.10 | 0.86 | 4.38 | 1.00 | 5.00 | -1.10 | 0.69 |
| Wave 4 | 1321 | 4.09 | 0.87 | 4.25 | 1.00 | 5.00 | -1.20 | 1.06 |
| Wave 5 | 1106 | 4.17 | 0.82 | 4.38 | 1.00 | 5.00 | -1.10 | 0.99 |
| Wave 6 | 906 | 4.10 | 0.85 | 4.25 | 1.00 | 5.00 | -1.00 | 0.50 |

Table 7

*Descriptive statistics of the Workplace Incivility Scale: Workplace bullying.*

|  | n | M | SD | Mdn | Min | Max | Skew | Kurt |
| --- | --- | --- | --- | --- | --- | --- | --- | --- |
| Wave 1 | 2435 | 1.78 | 0.89 | 1.50 | 1.00 | 5.00 | 1.45 | 1.75 |
| Wave 2 | 1749 | 1.65 | 0.80 | 1.50 | 1.00 | 5.00 | 1.74 | 3.08 |
| Wave 3 | 1378 | 1.65 | 0.76 | 1.50 | 1.00 | 5.00 | 1.50 | 2.10 |
| Wave 4 | 1330 | 1.62 | 0.77 | 1.25 | 1.00 | 5.00 | 1.70 | 2.99 |
| Wave 5 | 1134 | 1.64 | 0.75 | 1.50 | 1.00 | 5.00 | 1.55 | 2.30 |
| Wave 6 | 902 | 1.58 | 0.72 | 1.25 | 1.00 | 5.00 | 1.73 | 3.33 |

Table 8

*Life satisfaction (SWLS) two cluster model.*

|  | Est. | SE | z | Sig. |
| --- | --- | --- | --- | --- |
| C1\|C2 | -2.06 | 0.03 | -65.20 | *** |
| C1: W1 | 0.06 | 0.02 | 3.41 | ** |
| C1: W2 | 0.04 | 0.02 | 1.93 |  |
| C1: W3 | -0.04 | 0.02 | -1.93 |  |
| C1: W4 | -0.05 | 0.02 | -2.67 |  |
| C1: W5 | 0.02 | 0.02 | 0.83 |  |
| C1: W6 | -0.02 | 0.02 | -0.95 |  |
| C2: W1 | -0.15 | 0.03 | -5.40 | *** |
| C2: W2 | 0.06 | 0.03 | 2.03 |  |
| C2: W3 | 0.04 | 0.03 | 1.28 |  |
| C2: W4 | 0.02 | 0.03 | 0.65 |  |
| C2: W5 | -0.02 | 0.03 | -0.45 |  |
| C2: W6 | 0.05 | 0.04 | 1.26 |  |

Table 10

*Life satisfaction (SWLS) three cluster model.*

|  | Est. | SE | z | Sig. |
| --- | --- | --- | --- | --- |
| C1\|C2 | -1.18 | 0.02 | -50.46 | *** |
| C1\|C3 | -2.82 | 0.03 | -95.92 | *** |
| C2\|C3 | -1.64 | 0.03 | -52.52 | *** |
| C1: W1 | 0.04 | 0.02 | 1.67 |  |
| C1: W2 | 0.01 | 0.02 | 0.60 |  |
| C1: W3 | 0.01 | 0.02 | 0.34 |  |
| C1: W4 | -0.03 | 0.02 | -1.09 |  |
| C1: W5 | 0.01 | 0.03 | 0.27 |  |
| C1: W6 | -0.04 | 0.03 | -1.36 |  |
| C2: W1 | 0.03 | 0.03 | 1.22 |  |
| C2: W2 | 0.09 | 0.03 | 3.24 | * |
| C2: W3 | -0.12 | 0.03 | -3.93 | ** |
| C2: W4 | -0.11 | 0.03 | -3.88 | ** |
| C2: W5 | 0.08 | 0.03 | 2.43 |  |
| C2: W6 | 0.03 | 0.03 | 0.97 |  |
| C3: W1 | -0.18 | 0.04 | -4.97 | *** |
| C3: W2 | 0.03 | 0.04 | 0.82 |  |
| C3: W3 | 0.10 | 0.04 | 2.42 |  |
| C3: W4 | 0.12 | 0.04 | 2.91 |  |
| C3: W5 | -0.10 | 0.05 | -2.16 |  |
| C3: W6 | 0.03 | 0.05 | 0.55 |  |

Table 9

*Job satisfaction (MSQ) two cluster model.*

|  | Est. | SE | z | Sig. |
| --- | --- | --- | --- | --- |
| C1\|C2 | 0.58 | 0.01 | 53.73 | *** |
| C1: W1 | -0.07 | 0.01 | -6.16 | *** |
| C1: W2 | -0.01 | 0.01 | -0.56 |  |
| C1: W3 | 0.02 | 0.01 | 1.33 |  |
| C1: W4 | 0.04 | 0.01 | 3.16 | * |
| C1: W5 | 0.02 | 0.01 | 1.34 |  |
| C1: W6 | 0.01 | 0.01 | 0.49 |  |
| C2: W1 | -0.02 | 0.01 | -1.30 |  |
| C2: W2 | 0.00 | 0.01 | -0.20 |  |
| C2: W3 | -0.02 | 0.01 | -1.80 |  |
| C2: W4 | 0.00 | 0.01 | 0.19 |  |
| C2: W5 | 0.02 | 0.01 | 1.16 |  |
| C2: W6 | 0.02 | 0.01 | 1.53 |  |

Table 11

*Job satisfaction (MSQ) three cluster model.*

|  | Est. | SE | z | Sig. |
| --- | --- | --- | --- | --- |
| C1\|C2 | 0.48 | 0.01 | 52.47 | *** |
| C1\|C3 | -0.45 | 0.01 | -38.80 | *** |
| C2\|C3 | -0.92 | 0.01 | -76.32 | *** |
| C1: W1 | -0.06 | 0.01 | -5.38 | *** |
| C1: W2 | -0.01 | 0.01 | -0.62 |  |
| C1: W3 | 0.01 | 0.01 | 0.89 |  |
| C1: W4 | 0.02 | 0.01 | 1.96 |  |
| C1: W5 | 0.02 | 0.01 | 1.53 |  |
| C1: W6 | 0.02 | 0.01 | 1.20 |  |
| C2: W1 | 0.01 | 0.01 | 0.40 |  |
| C2: W2 | 0.00 | 0.01 | -0.10 |  |
| C2: W3 | -0.03 | 0.01 | -2.21 |  |
| C2: W4 | 0.00 | 0.01 | -0.12 |  |
| C2: W5 | 0.02 | 0.02 | 1.07 |  |
| C2: W6 | 0.01 | 0.02 | 0.77 |  |
| C3: W1 | -0.11 | 0.02 | -5.88 | *** |
| C3: W2 | -0.01 | 0.02 | -0.67 |  |
| C3: W3 | 0.03 | 0.02 | 1.31 |  |
| C3: W4 | 0.07 | 0.02 | 3.30 | * |
| C3: W5 | 0.02 | 0.02 | 0.96 |  |
| C3: W6 | 0.01 | 0.02 | 0.34 |  |

Table 12

*Life satisfaction (SWLS) four cluster model.*

|  | Est. | SE | z | Sig. |
| --- | --- | --- | --- | --- |
| C1\|C2 | -0.95 | 0.02 | -48.05 | *** |
| C1\|C3 | -2.09 | 0.02 | -88.05 | *** |
| C1\|C4 | -3.42 | 0.03 | -104.86 | *** |
| C2\|C3 | -1.13 | 0.03 | -45.04 | *** |
| C2\|C4 | -2.47 | 0.03 | -73.20 | *** |
| C3\|C4 | -1.34 | 0.04 | -36.93 | *** |
| C1: W1 | 0.04 | 0.02 | 1.66 |  |
| C1: W2 | 0.02 | 0.02 | 1.04 |  |
| C1: W3 | 0.00 | 0.03 | 0.20 |  |
| C1: W4 | -0.03 | 0.03 | -1.36 |  |
| C1: W5 | 0.01 | 0.03 | 0.28 |  |
| C1: W6 | -0.04 | 0.03 | -1.35 |  |
| C2: W1 | 0.09 | 0.03 | 3.49 | * |
| C2: W2 | 0.06 | 0.03 | 2.22 |  |
| C2: W3 | -0.12 | 0.03 | -4.02 | ** |
| C2: W4 | -0.09 | 0.03 | -3.08 |  |
| C2: W5 | 0.05 | 0.03 | 1.40 |  |
| C2: W6 | 0.01 | 0.04 | 0.36 |  |
| C3: W1 | -0.19 | 0.04 | -5.46 | *** |
| C3: W2 | 0.02 | 0.04 | 0.55 |  |
| C3: W3 | 0.07 | 0.04 | 1.86 |  |
| C3: W4 | 0.00 | 0.04 | 0.04 |  |
| C3: W5 | 0.03 | 0.04 | 0.71 |  |
| C3: W6 | 0.07 | 0.05 | 1.44 |  |
| C4: W1 | -0.16 | 0.05 | -2.99 |  |
| C4: W2 | 0.10 | 0.05 | 1.85 |  |
| C4: W3 | 0.10 | 0.06 | 1.63 |  |
| C4: W4 | 0.13 | 0.06 | 2.06 |  |
| C4: W5 | -0.15 | 0.07 | -2.25 |  |
| C4: W6 | -0.02 | 0.07 | -0.22 |  |

Table 13

*Job satisfaction (MSQ) four cluster model.*

|  | Est. | SE | z | Sig. |
| --- | --- | --- | --- | --- |
| C1\|C2 | 0.33 | 0.01 | 37.68 | *** |
| C1\|C3 | 0.71 | 0.01 | 71.59 | *** |
| C1\|C4 | -0.45 | 0.01 | -32.29 | *** |
| C2\|C3 | 0.38 | 0.01 | 37.30 | *** |
| C2\|C4 | -0.79 | 0.01 | -55.48 | *** |
| C3\|C4 | -1.16 | 0.01 | -78.23 | *** |
| C1: W1 | 0.02 | 0.01 | 1.81 |  |
| C1: W2 | -0.03 | 0.01 | -2.72 |  |
| C1: W3 | -0.01 | 0.01 | -0.55 |  |
| C1: W4 | 0.00 | 0.01 | -0.09 |  |
| C1: W5 | 0.00 | 0.01 | -0.26 |  |
| C1: W6 | 0.02 | 0.02 | 1.48 |  |
| C2: W1 | -0.12 | 0.01 | -9.12 | *** |
| C2: W2 | 0.04 | 0.01 | 3.01 |  |
| C2: W3 | 0.01 | 0.01 | 0.70 |  |
| C2: W4 | 0.04 | 0.01 | 2.94 |  |
| C2: W5 | 0.04 | 0.01 | 2.85 |  |
| C2: W6 | -0.01 | 0.02 | -0.60 |  |
| C3: W1 | 0.06 | 0.02 | 3.50 | * |
| C3: W2 | -0.03 | 0.02 | -1.88 |  |
| C3: W3 | -0.04 | 0.02 | -2.32 |  |
| C3: W4 | -0.02 | 0.02 | -0.90 |  |
| C3: W5 | -0.02 | 0.02 | -0.87 |  |
| C3: W6 | 0.05 | 0.02 | 2.29 |  |
| C4: W1 | -0.30 | 0.02 | -12.07 | *** |
| C4: W2 | -0.02 | 0.02 | -0.66 |  |
| C4: W3 | 0.04 | 0.03 | 1.68 |  |
| C4: W4 | 0.15 | 0.03 | 5.35 | *** |
| C4: W5 | 0.13 | 0.03 | 4.21 | *** |
| C4: W6 | -0.01 | 0.03 | -0.31 |  |

Table 14

*Life satisfaction (SWLS) five cluster model.*

|  | Est. | SE | | z | | Sig. | |
| --- | --- | --- | --- | --- | --- | --- | --- |
| C1\|C2 | 0.65 | 0.02 | 33.06 | | *** | |  |
| C1\|C3 | -0.84 | 0.02 | -41.18 | | *** | |  |
| C1\|C4 | -1.90 | 0.02 | -81.59 | | *** | |  |
| C1\|C5 | -3.19 | 0.03 | -93.46 | | *** | |  |
| C2\|C3 | -1.49 | 0.02 | -69.79 | | *** | |  |
| C2\|C4 | -2.55 | 0.02 | -105.57 | | *** | |  |
| C2\|C5 | -3.84 | 0.03 | -110.57 | | *** | |  |
| C3\|C4 | -1.06 | 0.02 | -43.09 | | *** | |  |
| C3\|C5 | -2.36 | 0.04 | -67.07 | | *** | |  |
| C4\|C5 | -1.29 | 0.04 | -35.01 | | *** | |  |
| C1: W1 | 0.02 | 0.03 | 0.76 | |  | |  |
| C1: W2 | 0.02 | 0.03 | 0.77 | |  | |  |
| C1: W3 | -0.01 | 0.03 | -0.30 | |  | |  |
| C1: W4 | -0.02 | 0.03 | -0.77 | |  | |  |
| C1: W5 | -0.02 | 0.03 | -0.76 | |  | |  |
| C1: W6 | 0.01 | 0.03 | 0.42 | |  | |  |
| C2: W1 | 0.05 | 0.03 | 1.67 | |  | |  |
| C2: W2 | 0.03 | 0.03 | 1.04 | |  | |  |
| C2: W3 | 0.00 | 0.03 | 0.02 | |  | |  |
| C2: W4 | -0.07 | 0.03 | -2.08 | |  | |  |
| C2: W5 | 0.03 | 0.03 | 1.01 | |  | |  |
| C2: W6 | -0.05 | 0.04 | -1.26 | |  | |  |
| C3: W1 | 0.12 | 0.03 | 3.80 | | ** | |  |
| C3: W2 | 0.05 | 0.03 | 1.76 | |  | |  |
| C3: W3 | -0.16 | 0.03 | -4.70 | | *** | |  |
| C3: W4 | -0.12 | 0.03 | -3.58 | | * | |  |
| C3: W5 | 0.10 | 0.04 | 2.74 | |  | |  |
| C3: W6 | 0.01 | 0.04 | 0.32 | |  | |  |
| C4: W1 | -0.30 | 0.04 | -7.85 | | *** | |  |
| C4: W2 | -0.02 | 0.04 | -0.41 | |  | |  |
| C4: W3 | 0.10 | 0.04 | 2.48 | |  | |  |
| C4: W4 | 0.18 | 0.04 | 4.11 | | ** | |  |
| C4: W5 | -0.07 | 0.05 | -1.52 | |  | |  |
| C4: W6 | 0.10 | 0.05 | 2.02 | |  | |  |
| C5: W1 | -0.20 | 0.06 | -3.36 | | * | |  |
| C5: W2 | 0.12 | 0.06 | 2.00 | |  | |  |
| C5: W3 | 0.11 | 0.07 | 1.60 | |  | |  |
| C5: W4 | 0.03 | 0.07 | 0.38 | |  | |  |
| C5: W5 | -0.05 | 0.08 | -0.60 | |  | |  |
| C5: W6 | -0.01 | 0.09 | -0.17 | |  | |  |

Table 15

*Job satisfaction (MSQ) five cluster model.*

|  | Est. | SE | z | Sig. |
| --- | --- | --- | --- | --- |
| C1\|C2 | 0.28 | 0.01 | 28.68 | *** |
| C1\|C3 | 0.75 | 0.01 | 73.43 | *** |
| C1\|C4 | 0.41 | 0.01 | 39.73 | *** |
| C1\|C5 | -0.44 | 0.01 | -31.54 | *** |
| C2\|C3 | 0.47 | 0.01 | 43.03 | *** |
| C2\|C4 | 0.13 | 0.01 | 11.68 | *** |
| C2\|C5 | -0.72 | 0.01 | -49.80 | *** |
| C3\|C4 | -0.34 | 0.01 | -30.02 | *** |
| C3\|C5 | -1.19 | 0.01 | -80.60 | *** |
| C4\|C5 | -0.85 | 0.01 | -57.29 | *** |
| C1: W1 | 0.02 | 0.01 | 1.20 |  |
| C1: W2 | 0.02 | 0.01 | 1.63 |  |
| C1: W3 | 0.01 | 0.01 | 0.92 |  |
| C1: W4 | -0.03 | 0.01 | -2.16 |  |
| C1: W5 | -0.05 | 0.01 | -3.19 |  |
| C1: W6 | 0.03 | 0.02 | 1.62 |  |
| C2: W1 | -0.32 | 0.02 | -20.31 | *** |
| C2: W2 | -0.11 | 0.01 | -7.55 | *** |
| C2: W3 | 0.02 | 0.02 | 1.06 |  |
| C2: W4 | 0.13 | 0.02 | 8.15 | *** |
| C2: W5 | 0.20 | 0.02 | 11.27 | *** |
| C2: W6 | 0.09 | 0.02 | 4.76 | *** |
| C3: W1 | -0.02 | 0.02 | -1.11 |  |
| C3: W2 | -0.04 | 0.02 | -2.17 |  |
| C3: W3 | -0.04 | 0.02 | -2.19 |  |
| C3: W4 | 0.01 | 0.02 | 0.70 |  |
| C3: W5 | 0.02 | 0.02 | 1.04 |  |
| C3: W6 | 0.06 | 0.02 | 2.98 |  |
| C4: W1 | 0.25 | 0.02 | 15.20 | *** |
| C4: W2 | 0.12 | 0.02 | 7.63 | *** |
| C4: W3 | -0.05 | 0.02 | -2.66 |  |
| C4: W4 | -0.09 | 0.02 | -4.96 | *** |
| C4: W5 | -0.12 | 0.02 | -6.35 | *** |
| C4: W6 | -0.12 | 0.02 | -5.84 | *** |
| C5: W1 | -0.32 | 0.02 | -13.18 | *** |
| C5: W2 | -0.04 | 0.02 | -1.69 |  |
| C5: W3 | 0.03 | 0.03 | 1.33 |  |
| C5: W4 | 0.17 | 0.03 | 6.29 | *** |
| C5: W5 | 0.16 | 0.03 | 5.27 | *** |
| C5: W6 | -0.01 | 0.03 | -0.28 |  |

Table 16

*Point-biserial correlations of cluster affiliations with gelotophobia.*

| Cluster solution | Cluster | | Life  Satisfaction | Job  Satisfaction | |
| --- | --- | --- | --- | --- | --- |
| 2 | 1 | -.19 | | | -.16 |
|  |  | (-.26 \| -.12) | | | (-.23 \| -.09) |
| 2 | 2 | .19 | | | .16 |
|  |  | (.12 \| .26) | | | (.09 \| .23) |
| 3 | 1 | -.21 | | | -.17 |
|  |  | (-.28 \| -.14) | | | (-.24 \| -.10) |
| 3 | 2 | .10 | | | .06 |
|  |  | (.04 \| .17) | | | (-.01 \| .12) |
| 3 | 3 | .15 | | | .14 |
|  |  | (.08 \| .22) | | | (.07 \| .21) |
| 4 | 1 | -.22 | | | -.17 |
|  |  | (-.29 \| -.15) | | | (-.24 \| -.10) |
| 4 | 2 | .05 | | | -.02 |
|  |  | (-.01 \| .11) | | | (-.07 \| .04) |
| 4 | 3 | .10 | | | .08 |
|  |  | (.04 \| .17) | | | (.01 \| .14) |
| 4 | 4 | .16 | | | .12 |
|  |  | (.09 \| .22) | | | (.05 \| .19) |
| 5 | 1 | -.20 | | | -.15 |
|  |  | (-.26 \| -.13) | | | (-.22 \| -.08) |
| 5 | 2 | -.03 | | | -.04 |
|  |  | (-.09 \| .02) | | | (-.10 \| .03) |
| 5 | 3 | .06 | | | -.01 |
|  |  | (-.01 \| .12) | | | (-.06 \| .04) |
| 5 | 4 | .09 | | | .06 |
|  |  | (.03 \| .15) | | | (.00 \| .13) |
| 5 | 5 | .15 | | | .13 |
|  |  | (.08 \| .22) | | | (.06 \| .20) |
