# Supplementary material for: Toward a dynamic model of Gelotophobia: Social support, workplace bullying and stress are connected with diverging trajectories of life and job satisfaction among Gelotophobes
Source: Curr Psychol. 2020 Sep 8;42(19):16368–80. doi: 10.1007/s12144-020-01046-y (PMC10404568; doi:10.1007/s12144-020-01046-y)
Supplement: Supplementary file 2 — (HTML 1.54 mb) [file 12144_2020_1046_MOESM2_ESM.html]

Toward a Dynamic Model of Gelotophobia: Social Support, Workplace Bullying and Stress Are Connected With Diverging Trajectories of Life and Job Satisfaction Among Gelotophobes and Non-Gelotophobes: Input and output


# Toward a Dynamic Model of Gelotophobia: Social Support, Workplace Bullying and Stress Are Connected With Diverging Trajectories of Life and Job Satisfaction Among Gelotophobes and Non-Gelotophobes: Input and output

#### Ruch, W., & Stahlmann, A. G.

#### June 5th, 2020

```
### Preparation ####
## Read packages
library(psych)
library(corrplot)
```

```
## corrplot 0.84 loaded
```

```
library(kml)
```

```
## Loading required package: clv
```

```
## Loading required package: cluster
```

```
## Loading required package: class
```

```
## Loading required package: longitudinalData
```

```
## Loading required package: rgl
```

```
## Loading required package: misc3d
```

```
library(plotrix)
```

```
## 
## Attaching package: 'plotrix'
```

```
## The following object is masked from 'package:rgl':
## 
##     mtext3d
```

```
## The following object is masked from 'package:psych':
## 
##     rescale
```

```
library(lmerTest)
```

```
## Loading required package: lme4
```

```
## Loading required package: Matrix
```

```
## 
## Attaching package: 'lmerTest'
```

```
## The following object is masked from 'package:lme4':
## 
##     lmer
```

```
## The following object is masked from 'package:stats':
## 
##     step
```

```
library(nlme)
```

```
## 
## Attaching package: 'nlme'
```

```
## The following object is masked from 'package:lme4':
## 
##     lmList
```

```
library(multcomp)
```

```
## Loading required package: mvtnorm
```

```
## Loading required package: survival
```

```
## Loading required package: TH.data
```

```
## Loading required package: MASS
```

```
## 
## Attaching package: 'TH.data'
```

```
## The following object is masked from 'package:MASS':
## 
##     geyser
```

```
## 
## Attaching package: 'multcomp'
```

```
## The following object is masked from 'package:kml':
## 
##     cld
```

```
library(car)
```

```
## Loading required package: carData
```

```
## Registered S3 methods overwritten by 'car':
##   method                          from
##   influence.merMod                lme4
##   cooks.distance.influence.merMod lme4
##   dfbeta.influence.merMod         lme4
##   dfbetas.influence.merMod        lme4
```

```
## 
## Attaching package: 'car'
```

```
## The following object is masked from 'package:psych':
## 
##     logit
```

```
## Options
options(max.print = 99999, digits = 5, warn = -1)
set.seed(555)

## Read data
setwd("/Users/astahlmann/Dropbox/AS/Schreiben/NCCR_gelotophobia/R1/")
load("PHO.RData")

## Decriptive analyses
PD = data.frame(PHO[, 1], rep(NA, nrow(PHO)), rep(NA, nrow(PHO)), rep(NA, nrow(PHO)), PHO[, 2], rep(NA, nrow(PHO)))
colnames(PD) = colnames(SWLS) = colnames(JSAT) = colnames(PSS) = colnames(GWSS) = colnames(DUFF) = colnames(WIS) = c("Wave 1", "Wave 2", "Wave 3", "Wave 4", "Wave 5", "Wave 6")

PD.D = describe(PD)[, c(2, 3, 4, 5, 8, 9, 11, 12)]
SWLS.D = describe(SWLS)[, c(2, 3, 4, 5, 8, 9, 11, 12)]
JSAT.D = describe(JSAT)[, c(2, 3, 4, 5, 8, 9, 11, 12)]
PSS.D = describe(PSS)[, c(2, 3, 4, 5, 8, 9, 11, 12)]
GWSS.D = describe(GWSS)[, c(2, 3, 4, 5, 8, 9, 11, 12)]
DUFF.D = describe(DUFF)[, c(2, 3, 4, 5, 8, 9, 11, 12)]
WIS.D = describe(WIS)[, c(2, 3, 4, 5, 8, 9, 11, 12)]

PD.D = format(round(PD.D, 2), 2)
SWLS.D = format(round(SWLS.D, 2), 2)
JSAT.D = format(round(JSAT.D, 2), 2)
PSS.D = format(round(PSS.D, 2), 2)
GWSS.D = format(round(GWSS.D, 2), 2)
DUFF.D = format(round(DUFF.D, 2), 2)
WIS.D = format(round(WIS.D, 2), 2)

PD.D
```

```
##           n mean   sd median min  max skew kurtosis
## Wave 1 2447 1.92 0.65   2.00   1    4 0.52    -0.13
## Wave 2    0  NaN   NA     NA Inf -Inf   NA       NA
## Wave 3    0  NaN   NA     NA Inf -Inf   NA       NA
## Wave 4    0  NaN   NA     NA Inf -Inf   NA       NA
## Wave 5 1112 1.91 0.65   1.67   1    4 0.58    -0.04
## Wave 6    0  NaN   NA     NA Inf -Inf   NA       NA
```

```
SWLS.D
```

```
##           n mean   sd median min max  skew kurtosis
## Wave 1 2448 5.03 1.27    5.4   1   7 -0.87     0.15
## Wave 2 1751 5.08 1.26    5.4   1   7 -0.98     0.46
## Wave 3 1320 5.12 1.25    5.4   1   7 -1.02     0.47
## Wave 4 1320 5.17 1.29    5.6   1   7 -1.05     0.56
## Wave 5 1102 5.14 1.22    5.6   1   7 -0.99     0.47
## Wave 6  904 5.16 1.29    5.6   1   7 -0.94     0.21
```

```
JSAT.D
```

```
##           n mean   sd median  min max  skew kurtosis
## Wave 1 1895 3.21 0.44   3.17 1.00   4 -0.24     0.13
## Wave 2 1633 3.18 0.47   3.17 1.00   4 -0.45     0.76
## Wave 3 1306 3.17 0.46   3.17 1.17   4 -0.27     0.28
## Wave 4 1267 3.15 0.48   3.17 1.00   4 -0.44     0.61
## Wave 5 1069 3.16 0.46   3.17 1.17   4 -0.39     0.52
## Wave 6  855 3.17 0.46   3.17 1.33   4 -0.29     0.62
```

```
PSS.D
```

```
##           n mean   sd median min  max skew kurtosis
## Wave 1 2445 2.39 0.66    2.4   1  5.0 0.37     0.29
## Wave 2 1753 2.38 0.65    2.4   1  5.0 0.45     0.17
## Wave 3 1325 2.41 0.67    2.4   1  5.0 0.51     0.39
## Wave 4    0  NaN   NA     NA Inf -Inf   NA       NA
## Wave 5 1104 2.34 0.65    2.4   1  5.0 0.44     0.34
## Wave 6  906 2.36 0.67    2.4   1  4.6 0.39     0.04
```

```
GWSS.D
```

```
##           n mean   sd median min  max skew kurtosis
## Wave 1 1891 1.87 0.58   1.78   1 4.56 0.74     0.56
## Wave 2 1507 1.90 0.62   1.78   1 5.00 0.82     0.88
## Wave 3 1201 1.88 0.65   1.78   1 5.00 0.95     1.00
## Wave 4 1183 1.85 0.65   1.78   1 4.89 0.99     1.20
## Wave 5  992 1.88 0.63   1.78   1 5.00 0.85     0.80
## Wave 6  801 1.89 0.67   1.78   1 5.00 0.97     1.25
```

```
DUFF.D
```

```
##           n mean   sd median min max  skew kurtosis
## Wave 1 2442 4.13 0.83   4.25   1   5 -1.17     1.10
## Wave 2 1756 4.15 0.84   4.38   1   5 -1.23     1.22
## Wave 3 1326 4.10 0.86   4.38   1   5 -1.10     0.69
## Wave 4 1321 4.09 0.87   4.25   1   5 -1.19     1.06
## Wave 5 1106 4.17 0.82   4.38   1   5 -1.13     0.99
## Wave 6  906 4.10 0.85   4.25   1   5 -1.04     0.50
```

```
WIS.D
```

```
##           n mean   sd median min max skew kurtosis
## Wave 1 2435 1.78 0.89   1.50   1   5 1.45     1.75
## Wave 2 1749 1.65 0.80   1.50   1   5 1.74     3.08
## Wave 3 1378 1.65 0.76   1.50   1   5 1.50     2.10
## Wave 4 1330 1.62 0.77   1.25   1   5 1.70     2.99
## Wave 5 1134 1.64 0.75   1.50   1   5 1.55     2.30
## Wave 6  902 1.58 0.72   1.25   1   5 1.73     3.33
```

```
write.csv(PD.D, "PD.D.csv", na = " ", row.names = T, col.names = T)
write.csv(SWLS.D, "SWLS.D.csv", na = " ", row.names = T, col.names = T)
write.csv(JSAT.D, "JST.D.csv", na = " ", row.names = T, col.names = T)
write.csv(PSS.D, "PSS.D.csv", na = " ", row.names = T, col.names = T)
write.csv(GWSS.D, "GWSS.D.csv", na = " ", row.names = T, col.names = T)
write.csv(DUFF.D, "DUFF.D.csv", na = " ", row.names = T, col.names = T)
write.csv(WIS.D, "WIS.D.csv", na = " ", row.names = T, col.names = T)

### Preliminary Correlational Analyses ####
CT1 = corr.test(PHO[, 3], SWLS)
CT2 = corr.test(PHO[, 3], JSAT)
CT3 = corr.test(PHO[, 3], PSS)
CT4 = corr.test(PHO[, 3], GWSS)
CT5 = corr.test(PHO[, 3], DUFF)
CT6 = corr.test(PHO[, 3], WIS)

CT = rbind(CT1[[1]], CT2[[1]], CT3[[1]], CT4[[1]], CT5[[1]], CT6[[1]])
PT = rbind(CT1[[4]], CT2[[4]], CT3[[4]], CT4[[4]], CT5[[4]], CT6[[4]])

colnames(CT) = c("W1", "W2", "W3", "W4", "W5", "W6")
rownames(CT) = c("SWLS", "JSAT", "PSS", "GWSS", "DUFF", "WIS")

corrplot(CT, outline = F, method = "pie", p.mat = PT, sig.level = .05, insig = "pch", diag = T, tl.col = 1)
```

```
print(CT1, short = F)
```

```
## Call:corr.test(x = PHO[, 3], y = SWLS)
## Correlation matrix 
##      Wave 1 Wave 2 Wave 3 Wave 4 Wave 5 Wave 6
## [1,]  -0.21   -0.2  -0.21  -0.26  -0.25  -0.24
## Sample Size 
##      Wave 1 Wave 2 Wave 3 Wave 4 Wave 5 Wave 6
## [1,]   2444   1648   1274   1279   1101    893
## Probability values  adjusted for multiple tests. 
##      Wave 1 Wave 2 Wave 3 Wave 4 Wave 5 Wave 6
## [1,]      0      0      0      0      0      0
## 
##  Confidence intervals based upon normal theory.  To get bootstrapped values, try cor.ci
##          raw.lower raw.r raw.upper raw.p lower.adj upper.adj
## NA-Wave1     -0.25 -0.21     -0.18     0     -0.26     -0.17
## NA-Wave2     -0.24 -0.20     -0.15     0     -0.24     -0.15
## NA-Wave3     -0.26 -0.21     -0.15     0     -0.27     -0.15
## NA-Wave4     -0.31 -0.26     -0.21     0     -0.33     -0.19
## NA-Wave5     -0.31 -0.25     -0.20     0     -0.32     -0.18
## NA-Wave6     -0.30 -0.24     -0.18     0     -0.32     -0.16
```

```
print(CT2, short = F)
```

```
## Call:corr.test(x = PHO[, 3], y = JSAT)
## Correlation matrix 
##      Wave 1 Wave 2 Wave 3 Wave 4 Wave 5 Wave 6
## [1,]  -0.15  -0.16  -0.17  -0.16  -0.14  -0.11
## Sample Size 
##      Wave 1 Wave 2 Wave 3 Wave 4 Wave 5 Wave 6
## [1,]   1882   1507   1231   1211   1040    829
## Probability values  adjusted for multiple tests. 
##      Wave 1 Wave 2 Wave 3 Wave 4 Wave 5 Wave 6
## [1,]      0      0      0      0      0      0
## 
##  Confidence intervals based upon normal theory.  To get bootstrapped values, try cor.ci
##          raw.lower raw.r raw.upper raw.p lower.adj upper.adj
## NA-Wave1     -0.19 -0.15     -0.11     0     -0.20     -0.10
## NA-Wave2     -0.21 -0.16     -0.11     0     -0.23     -0.10
## NA-Wave3     -0.22 -0.17     -0.11     0     -0.24     -0.09
## NA-Wave4     -0.21 -0.16     -0.10     0     -0.23     -0.09
## NA-Wave5     -0.20 -0.14     -0.08     0     -0.21     -0.07
## NA-Wave6     -0.18 -0.11     -0.04     0     -0.18     -0.04
```

```
print(CT3, short = F)
```

```
## Call:corr.test(x = PHO[, 3], y = PSS)
## Correlation matrix 
##      Wave 1 Wave 2 Wave 3 Wave 4 Wave 5 Wave 6
## [1,]   0.34    0.3    0.3     NA   0.36   0.34
## Sample Size 
##      Wave 1 Wave 2 Wave 3 Wave 4 Wave 5 Wave 6
## [1,]   2444   1650   1279      0   1103    895
## Probability values  adjusted for multiple tests. 
##      Wave 1 Wave 2 Wave 3 Wave 4 Wave 5 Wave 6
## [1,]      0      0      0     NA      0      0
## 
##  Confidence intervals based upon normal theory.  To get bootstrapped values, try cor.ci
##          raw.lower raw.r raw.upper raw.p lower.adj upper.adj
## NA-Wave1      0.31  0.34      0.38     0      0.30      0.38
## NA-Wave2      0.25  0.30      0.34     0      0.25      0.34
## NA-Wave3      0.25  0.30      0.35     0      0.24      0.36
## NA-Wave4        NA    NA        NA    NA        NA        NA
## NA-Wave5      0.31  0.36      0.41     0      0.29      0.42
## NA-Wave6      0.28  0.34      0.39     0      0.26      0.41
```

```
print(CT4, short = F)
```

```
## Call:corr.test(x = PHO[, 3], y = GWSS)
## Correlation matrix 
##      Wave 1 Wave 2 Wave 3 Wave 4 Wave 5 Wave 6
## [1,]   0.24   0.24   0.25   0.25   0.26   0.21
## Sample Size 
##      Wave 1 Wave 2 Wave 3 Wave 4 Wave 5 Wave 6
## [1,]   1882   1420   1161   1149    989    788
## Probability values  adjusted for multiple tests. 
##      Wave 1 Wave 2 Wave 3 Wave 4 Wave 5 Wave 6
## [1,]      0      0      0      0      0      0
## 
##  Confidence intervals based upon normal theory.  To get bootstrapped values, try cor.ci
##          raw.lower raw.r raw.upper raw.p lower.adj upper.adj
## NA-Wave1      0.20  0.24      0.28     0      0.19      0.29
## NA-Wave2      0.19  0.24      0.29     0      0.18      0.30
## NA-Wave3      0.20  0.25      0.30     0      0.18      0.32
## NA-Wave4      0.20  0.25      0.30     0      0.18      0.32
## NA-Wave5      0.20  0.26      0.32     0      0.18      0.34
## NA-Wave6      0.15  0.21      0.28     0      0.15      0.28
```

```
print(CT5, short = F)
```

```
## Call:corr.test(x = PHO[, 3], y = DUFF)
## Correlation matrix 
##      Wave 1 Wave 2 Wave 3 Wave 4 Wave 5 Wave 6
## [1,]  -0.26  -0.21  -0.21  -0.26   -0.3  -0.29
## Sample Size 
##      Wave 1 Wave 2 Wave 3 Wave 4 Wave 5 Wave 6
## [1,]   2440   1651   1280   1279   1105    895
## Probability values  adjusted for multiple tests. 
##      Wave 1 Wave 2 Wave 3 Wave 4 Wave 5 Wave 6
## [1,]      0      0      0      0      0      0
## 
##  Confidence intervals based upon normal theory.  To get bootstrapped values, try cor.ci
##          raw.lower raw.r raw.upper raw.p lower.adj upper.adj
## NA-Wave1     -0.29 -0.26     -0.22     0     -0.30     -0.21
## NA-Wave2     -0.26 -0.21     -0.17     0     -0.26     -0.17
## NA-Wave3     -0.27 -0.21     -0.16     0     -0.27     -0.15
## NA-Wave4     -0.31 -0.26     -0.21     0     -0.32     -0.19
## NA-Wave5     -0.35 -0.30     -0.24     0     -0.37     -0.22
## NA-Wave6     -0.34 -0.29     -0.22     0     -0.36     -0.20
```

```
print(CT6, short = F)
```

```
## Call:corr.test(x = PHO[, 3], y = WIS)
## Correlation matrix 
##      Wave 1 Wave 2 Wave 3 Wave 4 Wave 5 Wave 6
## [1,]   0.11   0.14   0.14   0.15   0.18   0.13
## Sample Size 
##      Wave 1 Wave 2 Wave 3 Wave 4 Wave 5 Wave 6
## [1,]   2419   1607   1294   1268   1103    873
## Probability values  adjusted for multiple tests. 
##      Wave 1 Wave 2 Wave 3 Wave 4 Wave 5 Wave 6
## [1,]      0      0      0      0      0      0
## 
##  Confidence intervals based upon normal theory.  To get bootstrapped values, try cor.ci
##          raw.lower raw.r raw.upper raw.p lower.adj upper.adj
## NA-Wave1      0.07  0.11      0.15     0      0.07      0.15
## NA-Wave2      0.09  0.14      0.18     0      0.08      0.19
## NA-Wave3      0.09  0.14      0.20     0      0.08      0.21
## NA-Wave4      0.09  0.15      0.20     0      0.07      0.22
## NA-Wave5      0.12  0.18      0.24     0      0.10      0.25
## NA-Wave6      0.07  0.13      0.20     0      0.06      0.21
```

```
AG = data.frame(rowMeans(SWLS, na.rm = T), rowMeans(JSAT, na.rm = T),
                rowMeans(PSS, na.rm = T), rowMeans(GWSS, na.rm = T),
                rowMeans(DUFF, na.rm = T), rowMeans(WIS, na.rm = T), PHO$P_M)
colnames(AG) = c("SWLS", "JSAT", "PSS", "GWSS", "DUFF", "WIS", "PHO")
AG[is.na(AG)] = NA

CT = corr.test(AG)
corrplot(CT$r, outline = F, method = "pie", p.mat = CT$p, sig.level = .05, insig = "pch", diag = F, tl.col = 1)
```

```
N = CT$n-1
N1 = N[c(1, 2, 7), c(1, 2, 7)]

C0 = CT$r
C1 = partial.r(CT$r, c(1:2, 7), 3)
C2 = partial.r(CT$r, c(1:2, 7), 4)
C3 = partial.r(CT$r, c(1:2, 7), 5)
C4 = partial.r(CT$r, c(1:2, 7), 6)
C5 = partial.r(CT$r, c(1:2, 7), 3:6)

P0 = corr.p(C0, N)$ci.adj[c(6, 11), ]
P1 = corr.p(C1, N1)$ci.adj[c(2, 3), ]
P2 = corr.p(C2, N1)$ci.adj[c(2, 3), ]
P3 = corr.p(C3, N1)$ci.adj[c(2, 3), ]
P4 = corr.p(C4, N1)$ci.adj[c(2, 3), ]
P5 = corr.p(C5, N1)$ci.adj[c(3, 6), ]

CPHO = data.frame(CT$r[1:2, 7],
                  partial.r(CT$r, c(1:2, 7), 3)[1:2, 3], partial.r(CT$r, c(1:2, 7), 4)[1:2, 3],
                  partial.r(CT$r, c(1:2, 7), 5)[1:2, 3], partial.r(CT$r, c(1:2, 7), 6)[1:2, 3],
                  partial.r(CT$r, c(1:2, 7), 3:6)[1:2, 3])
colnames(CPHO) = c("Zero-order", "C-PSS", "C-GWSS", "C-DUFF", "C-WIS", "C-ALL")
CPHO = round(CPHO, 2)
CPHO
```

```
##      Zero-order C-PSS C-GWSS C-DUFF C-WIS C-ALL
## SWLS      -0.23  0.00  -0.16  -0.09 -0.20  0.05
## JSAT      -0.17 -0.06  -0.06  -0.10 -0.12  0.00
```

```
### Multidirectional Trajectories of Well-being ####
M6 = cbind(TW$D_USERNAME[1:2759], SWLS)
J6 = cbind(TW$D_USERNAME[1:2759], JSAT)
colnames(M6) = c("ID", paste("SWLS", 1:6, sep = "_"))
colnames(J6) = c("ID", paste("JSAT", 1:6, sep = "_"))

# M6 = M6[order(M6$ID), ]
# J6 = J6[order(J6$ID), ]

M2 = clusterLongData(M6[, 2:7], M6$ID, c(1:6))
M3 = clusterLongData(J6[, 2:7], J6$ID, c(1:6))
kml(M2)
```

```
##  ~ Fast KmL ~
## ***************************************************************************************************S
## 100 S
```

```
kml(M3)
```

```
##  ~ Fast KmL ~
## ***************************************************************************************************S
## 100 S
```

```
## Calinski/Harabasz-Criteria
# SWLS
M2C = NULL
M3C = NULL
M4C = NULL
M5C = NULL
for(i in 1:20) {
  M2C = c(M2C, M2@c2[[i]]@criterionValues[[3]])
  M3C = c(M3C, M2@c3[[i]]@criterionValues[[3]])
  M4C = c(M4C, M2@c4[[i]]@criterionValues[[3]])
  M5C = c(M5C, M2@c5[[i]]@criterionValues[[3]])
}
c(min(M2C), min(M3C), min(M4C), min(M5C))
```

```
## [1] 2491.7 2953.6 3007.5 2731.9
```

```
# JSAT
M2C = NULL
M3C = NULL
M4C = NULL
M5C = NULL
for(i in 1:20) {
  M2C = c(M2C, M3@c2[[i]]@criterionValues[[3]])
  M3C = c(M3C, M3@c3[[i]]@criterionValues[[3]])
  M4C = c(M4C, M3@c4[[i]]@criterionValues[[3]])
  M5C = c(M5C, M3@c5[[i]]@criterionValues[[3]])
}
c(min(M2C), min(M3C), min(M4C), min(M5C))
```

```
## [1] 1187.0 1331.2 1257.7 1204.4
```

```
## Dummy-coding for further analyses
M6$SWLS_C2 = as.integer(getClusters(M2, 2))
M6$SWLS_C3 = as.integer(getClusters(M2, 3))
M6$SWLS_C4 = as.integer(getClusters(M2, 4))
M6$SWLS_C5 = as.integer(getClusters(M2, 5))

J6$JSAT_C2 = as.integer(getClusters(M3, 2))
J6$JSAT_C3 = as.integer(getClusters(M3, 3))
J6$JSAT_C4 = as.integer(getClusters(M3, 4))
J6$JSAT_C5 = as.integer(getClusters(M3, 5))


PHO$C2_1 = rep(0, 2759)
PHO$C2_2 = rep(0, 2759)

PHO$C3_1 = rep(0, 2759)
PHO$C3_2 = rep(0, 2759)
PHO$C3_3 = rep(0, 2759)

PHO$C4_1 = rep(0, 2759)
PHO$C4_2 = rep(0, 2759)
PHO$C4_3 = rep(0, 2759)
PHO$C4_4 = rep(0, 2759)

PHO$J2_1 = rep(0, 2759)
PHO$J2_2 = rep(0, 2759)

PHO$J3_1 = rep(0, 2759)
PHO$J3_2 = rep(0, 2759)
PHO$J3_3 = rep(0, 2759)

PHO$J4_1 = rep(0, 2759)
PHO$J4_2 = rep(0, 2759)
PHO$J4_3 = rep(0, 2759)
PHO$J4_4 = rep(0, 2759)


colnames(PHO) = c("PHO_1", "PHO_2", "PHO_M", "CPHO_1", "CPHO_2", "CPHO_M", "C2_1", "C2_2", "C3_1", "C3_2", "C3_3", "C4_1", "C4_2", "C4_3", "C4_4",
                  "J2_1", "J2_2", "J3_1", "J3_2", "J3_3", "J4_1", "J4_2", "J4_3", "J4_4")

for(i in 1:2759) {
  # SWLS
  if(is.na(M6[i, 8])) {
    PHO$C2_1[i] = NA
    PHO$C2_2[i] = NA
  } else {
    if(M6[i, 8] == 1) {
      PHO$C2_1[i] = 1
    } else if(M6[i, 8] == 2) {
      PHO$C2_2[i] = 1
    }
  }
  if(is.na(M6[i, 9])) {
    PHO$C3_1[i] = NA
    PHO$C3_2[i] = NA
    PHO$C3_3[i] = NA
  } else {
    if(M6[i, 9] == 1) {
      PHO$C3_1[i] = 1
    } else if(M6[i, 9] == 2) {
      PHO$C3_2[i] = 1
    } else if(M6[i, 9] == 3) {
      PHO$C3_3[i] = 1
    }
  }
  if(is.na(M6[i, 10])) {
    PHO$C4_1[i] = NA
    PHO$C4_2[i] = NA
    PHO$C4_3[i] = NA
    PHO$C4_4[i] = NA
  } else {
    if(M6[i, 10] == 1) {
      PHO$C4_1[i] = 1
    } else if(M6[i, 10] == 2) {
      PHO$C4_2[i] = 1
    } else if(M6[i, 10] == 3) {
      PHO$C4_3[i] = 1
    } else if(M6[i, 10] == 4) {
      PHO$C4_4[i] = 1
    }
  }
  # JSAT
  if(is.na(J6[i, 8])) {
    PHO$J2_1[i] = NA
    PHO$J2_2[i] = NA
  } else {
    if(J6[i, 8] == 1) {
      PHO$J2_1[i] = 1
    } else if(J6[i, 8] == 2) {
      PHO$J2_2[i] = 1
    }
  }
  if(is.na(J6[i, 9])) {
    PHO$J3_1[i] = NA
    PHO$J3_2[i] = NA
    PHO$J3_3[i] = NA
  } else {
    if(J6[i, 9] == 1) {
      PHO$J3_1[i] = 1
    } else if(J6[i, 9] == 2) {
      PHO$J3_2[i] = 1
    } else if(J6[i, 9] == 3) {
      PHO$J3_3[i] = 1
    }
  }
  if(is.na(J6[i, 10])) {
    PHO$J4_1[i] = NA
    PHO$J4_2[i] = NA
    PHO$J4_3[i] = NA
    PHO$J4_4[i] = NA
  } else {
    if(J6[i, 10] == 1) {
      PHO$J4_1[i] = 1
    } else if(J6[i, 10] == 2) {
      PHO$J4_2[i] = 1
    } else if(J6[i, 10] == 3) {
      PHO$J4_3[i] = 1
    } else if(J6[i, 10] == 4) {
      PHO$J4_4[i] = 1
    }
  }
}

PHO[is.na(PHO)] = NA
PHO = data.frame(TW$D_USERNAME[1:2759], PHO)
colnames(PHO)[1] = "ID"
PHO = PHO[order(PHO$ID), ] 

## Mean differences and percentages
aggregate(rowMeans(M6[, 2:7], na.rm = T), list(M6$SWLS_C2), mean)
```

```
##   Group.1      x
## 1       1 5.7122
## 2       2 3.6605
```

```
aggregate(rowMeans(M6[, 2:7], na.rm = T), list(M6$SWLS_C3), mean)
```

```
##   Group.1      x
## 1       1 5.9865
## 2       2 4.8063
## 3       3 3.1647
```

```
aggregate(rowMeans(J6[, 2:7], na.rm = T), list(J6$JSAT_C2), mean)
```

```
##   Group.1      x
## 1       1 2.9071
## 2       2 3.4991
```

```
aggregate(rowMeans(J6[, 2:7], na.rm = T), list(J6$JSAT_C3), mean)
```

```
##   Group.1      x
## 1       1 3.0932
## 2       2 3.5713
## 3       3 2.6392
```

```
table(M6$SWLS_C2)/1902*100
```

```
## 
##      1      2 
## 69.979 30.021
```

```
table(M6$SWLS_C2, M6$SWLS_C3)
```

```
##    
##       1   2   3
##   1 936 395   0
##   2   0 234 337
```

```
936/(936+395)*100
```

```
## [1] 70.323
```

```
234/(234+337)*100
```

```
## [1] 40.981
```

```
table(J6$JSAT_C2)/1769*100
```

```
## 
##      1      2 
## 54.664 45.336
```

```
table(J6$JSAT_C2, J6$JSAT_C3)
```

```
##    
##       1   2   3
##   1 641   0 326
##   2 188 614   0
```

```
641/(641+326)*100
```

```
## [1] 66.287
```

```
188/(188+614)*100
```

```
## [1] 23.441
```

```
## Figure 1. Temporally aggregated trajectories of the three-cluster-solutions for life and job satisfaction respectively.
# pdf("NCCR_KML.pdf", width = 12, height = 6, pointsize = 14)
par(mfrow = c(1, 2))

## SWLS
plot(NA, type = "l", xlim = c(.5, 6.5), ylim = c(1, 7), col = "gray",
     las = 1, xlab = "Measurement interval", ylab = "Life satisfaction",
     cex.lab = 1.2, cex.axis = 1.2)
box(lwd = 1.2)

abline(h = c(1:7), lwd = .5, lty = 2)

S1M = apply(subset(M6[, c(2:7)], M6$SWLS_C3 == 1), 2, mean, na.rm = T)
S2M = apply(subset(M6[, c(2:7)], M6$SWLS_C3 == 2), 2, mean, na.rm = T)
S3M = apply(subset(M6[, c(2:7)], M6$SWLS_C3 == 3), 2, mean, na.rm = T)

lines(S1M, type = "b", pch = 0, lwd = 2, cex = 1)
lines(S2M, type = "b", pch = 0, lwd = 2, cex = 1)
lines(S3M, type = "b", pch = 0, lwd = 2, cex = 1)

draw.ellipse(1, S1M[1], .6, .35, col = "white", lwd = 2)
draw.ellipse(1, S2M[1], .6, .35, col = "white", lwd = 2)
draw.ellipse(1, S3M[1], .6, .35, col = "white", lwd = 2)

text(S1M[1], labels = "LS 3/1", cex = 1.15)
text(S2M[1], labels = "LS 3/2", cex = 1.15)
text(S3M[1], labels = "LS 3/3", cex = 1.15)

## JSAT
plot(NA, type = "l", xlim = c(.5, 6.5), ylim = c(1, 4), col = "gray",
     las = 1, xlab = "Measurement interval", ylab = "Job satisfaction",
     cex.lab = 1.2, cex.axis = 1.2)
box(lwd = 1.2)

abline(h = seq(1, 4, .5), lwd = .5, lty = 2)

J1M = apply(subset(J6[, c(2:7)], J6$JSAT_C3 == 2), 2, mean, na.rm = T)
J2M = apply(subset(J6[, c(2:7)], J6$JSAT_C3 == 1), 2, mean, na.rm = T)
J3M = apply(subset(J6[, c(2:7)], J6$JSAT_C3 == 3), 2, mean, na.rm = T)

lines(J1M, type = "b", pch = 0, lwd = 2, cex = 1)
lines(J2M, type = "b", pch = 0, lwd = 2, cex = 1)
lines(J3M, type = "b", pch = 0, lwd = 2, cex = 1)

draw.ellipse(1, J1M[1], .6, .16, col = "white", lwd = 2)
draw.ellipse(1, J2M[1], .6, .16, col = "white", lwd = 2)
draw.ellipse(1, J3M[1], .6, .16, col = "white", lwd = 2)

text(J1M[1], labels = "JS 3/1", cex = 1.15)
text(J2M[1], labels = "JS 3/2", cex = 1.15)
text(J3M[1], labels = "JS 3/3", cex = 1.15)
```

```
# dev.off()


## LMM levelsignificance and flatness
# Contrast matrices
CH2 = rbind("G1|G2" = c(-1/6, -1/6, -1/6, -1/6, -1/6, -1/6, 1/6, 1/6, 1/6, 1/6, 1/6, 1/6),
            "G1: M1" = c(-5/6, 1/6, 1/6, 1/6, 1/6, 1/6, 0, 0, 0, 0, 0, 0),
            "G1: M2" = c(1/6, -5/6, 1/6, 1/6, 1/6, 1/6, 0, 0, 0, 0, 0, 0),
            "G1: M3" = c(1/6, 1/6, -5/6, 1/6, 1/6, 1/6, 0, 0, 0, 0, 0, 0),
            "G1: M4" = c(1/6, 1/6, 1/6, -5/6, 1/6, 1/6, 0, 0, 0, 0, 0, 0),
            "G1: M5" = c(1/6, 1/6, 1/6, 1/6, -5/6, 1/6, 0, 0, 0, 0, 0, 0),
            "G1: M6" = c(1/6, 1/6, 1/6, 1/6, 1/6, -5/6, 0, 0, 0, 0, 0, 0),
            "G2: M1" = c(0, 0, 0, 0, 0, 0, -5/6, 1/6, 1/6, 1/6, 1/6, 1/6),
            "G2: M2" = c(0, 0, 0, 0, 0, 0, 1/6, -5/6, 1/6, 1/6, 1/6, 1/6),
            "G2: M3" = c(0, 0, 0, 0, 0, 0, 1/6, 1/6, -5/6, 1/6, 1/6, 1/6),
            "G2: M4" = c(0, 0, 0, 0, 0, 0, 1/6, 1/6, 1/6, -5/6, 1/6, 1/6),
            "G2: M5" = c(0, 0, 0, 0, 0, 0, 1/6, 1/6, 1/6, 1/6, -5/6, 1/6),
            "G2: M6" = c(0, 0, 0, 0, 0, 0, 1/6, 1/6, 1/6, 1/6, 1/6, -5/6))

CH3 = rbind("G1|G2" = c(-1/6, -1/6, -1/6, -1/6, -1/6, -1/6, 1/6, 1/6, 1/6, 1/6, 1/6, 1/6, 0, 0, 0, 0, 0, 0),
            "G1|G3" = c(-1/6, -1/6, -1/6, -1/6, -1/6, -1/6, 0, 0, 0, 0, 0, 0, 1/6, 1/6, 1/6, 1/6, 1/6, 1/6),
            "G2|G3" = c(0, 0, 0, 0, 0, 0, -1/6, -1/6, -1/6, -1/6, -1/6, -1/6, 1/6, 1/6, 1/6, 1/6, 1/6, 1/6),
            "G1: M1" = c(-5/6, 1/6, 1/6, 1/6, 1/6, 1/6, 0, 0, 0, 0, 0, 0, 0, 0, 0, 0, 0, 0),
            "G1: M2" = c(1/6, -5/6, 1/6, 1/6, 1/6, 1/6, 0, 0, 0, 0, 0, 0, 0, 0, 0, 0, 0, 0),
            "G1: M3" = c(1/6, 1/6, -5/6, 1/6, 1/6, 1/6, 0, 0, 0, 0, 0, 0, 0, 0, 0, 0, 0, 0),
            "G1: M4" = c(1/6, 1/6, 1/6, -5/6, 1/6, 1/6, 0, 0, 0, 0, 0, 0, 0, 0, 0, 0, 0, 0),
            "G1: M5" = c(1/6, 1/6, 1/6, 1/6, -5/6, 1/6, 0, 0, 0, 0, 0, 0, 0, 0, 0, 0, 0, 0),
            "G1: M6" = c(1/6, 1/6, 1/6, 1/6, 1/6, -5/6, 0, 0, 0, 0, 0, 0, 0, 0, 0, 0, 0, 0),
            "G2: M1" = c(0, 0, 0, 0, 0, 0, -5/6, 1/6, 1/6, 1/6, 1/6, 1/6, 0, 0, 0, 0, 0, 0),
            "G2: M2" = c(0, 0, 0, 0, 0, 0, 1/6, -5/6, 1/6, 1/6, 1/6, 1/6, 0, 0, 0, 0, 0, 0),
            "G2: M3" = c(0, 0, 0, 0, 0, 0, 1/6, 1/6, -5/6, 1/6, 1/6, 1/6, 0, 0, 0, 0, 0, 0),
            "G2: M4" = c(0, 0, 0, 0, 0, 0, 1/6, 1/6, 1/6, -5/6, 1/6, 1/6, 0, 0, 0, 0, 0, 0),
            "G2: M5" = c(0, 0, 0, 0, 0, 0, 1/6, 1/6, 1/6, 1/6, -5/6, 1/6, 0, 0, 0, 0, 0, 0),
            "G2: M6" = c(0, 0, 0, 0, 0, 0, 1/6, 1/6, 1/6, 1/6, 1/6, -5/6, 0, 0, 0, 0, 0, 0),
            "G3: M1" = c(0, 0, 0, 0, 0, 0, 0, 0, 0, 0, 0, 0, -5/6, 1/6, 1/6, 1/6, 1/6, 1/6),
            "G3: M2" = c(0, 0, 0, 0, 0, 0, 0, 0, 0, 0, 0, 0, 1/6, -5/6, 1/6, 1/6, 1/6, 1/6),
            "G3: M3" = c(0, 0, 0, 0, 0, 0, 0, 0, 0, 0, 0, 0, 1/6, 1/6, -5/6, 1/6, 1/6, 1/6),
            "G3: M4" = c(0, 0, 0, 0, 0, 0, 0, 0, 0, 0, 0, 0, 1/6, 1/6, 1/6, -5/6, 1/6, 1/6),
            "G3: M5" = c(0, 0, 0, 0, 0, 0, 0, 0, 0, 0, 0, 0, 1/6, 1/6, 1/6, 1/6, -5/6, 1/6),
            "G3: M6" = c(0, 0, 0, 0, 0, 0, 0, 0, 0, 0, 0, 0, 1/6, 1/6, 1/6, 1/6, 1/6, -5/6))

## SWLS
# C2
GM6 = data.frame(rep(c(1:nrow(M6)), 6), rep(M6$SWLS_C2, 6), rep(c(1:6), each = nrow(M6)), c(M6$SWLS_1, M6$SWLS_2, M6$SWLS_3, M6$SWLS_4, M6$SWLS_5, M6$SWLS_6))
colnames(GM6) = c("ID", "CLASS", "MZP", "SWLS")
GM6$ID = factor(GM6$ID)
GM6$CLASS = factor(GM6$CLASS)
GM6$MZP = factor(GM6$MZP)
GM6 = na.omit(GM6)
GM6$INT = interaction(GM6$MZP, GM6$CLASS)

GM6$INT
```

```
##    [1] 1.2 1.1 1.1 1.2 1.1 1.1 1.1 1.1 1.2 1.1 1.1 1.2 1.2 1.2 1.1 1.1 1.1 1.1
##   [19] 1.1 1.1 1.2 1.1 1.1 1.1 1.1 1.1 1.2 1.2 1.1 1.2 1.2 1.2 1.1 1.2 1.2 1.1
##   [37] 1.1 1.2 1.1 1.2 1.2 1.2 1.2 1.1 1.2 1.1 1.2 1.1 1.2 1.2 1.2 1.2 1.2 1.2
##   [55] 1.1 1.2 1.2 1.1 1.1 1.1 1.2 1.1 1.2 1.1 1.1 1.1 1.1 1.1 1.1 1.2 1.1 1.1
##   [73] 1.1 1.1 1.1 1.2 1.2 1.2 1.1 1.1 1.1 1.1 1.1 1.2 1.1 1.1 1.1 1.1 1.1 1.1
##   [91] 1.2 1.2 1.1 1.2 1.2 1.1 1.1 1.1 1.2 1.2 1.1 1.2 1.2 1.2 1.2 1.2 1.1 1.2
##  [109] 1.2 1.1 1.1 1.2 1.1 1.2 1.2 1.2 1.1 1.1 1.2 1.2 1.2 1.2 1.2 1.2 1.2 1.2
##  [127] 1.2 1.2 1.1 1.2 1.2 1.2 1.1 1.2 1.2 1.1 1.2 1.2 1.2 1.1 1.1 1.2 1.2 1.2
##  [145] 1.2 1.2 1.2 1.1 1.1 1.1 1.2 1.1 1.2 1.2 1.1 1.2 1.1 1.1 1.1 1.2 1.2 1.1
##  [163] 1.1 1.1 1.1 1.1 1.2 1.1 1.1 1.1 1.1 1.2 1.2 1.1 1.1 1.1 1.1 1.2 1.2 1.2
##  [181] 1.1 1.1 1.2 1.2 1.2 1.2 1.2 1.1 1.1 1.1 1.2 1.1 1.2 1.2 1.1 1.2 1.2 1.2
##  [199] 1.2 1.1 1.1 1.1 1.1 1.1 1.1 1.2 1.1 1.2 1.2 1.1 1.1 1.2 1.2 1.1 1.2 1.1
##  [217] 1.1 1.1 1.2 1.1 1.2 1.1 1.2 1.2 1.1 1.1 1.1 1.1 1.2 1.1 1.1 1.1 1.2 1.1
##  [235] 1.2 1.2 1.2 1.1 1.1 1.1 1.2 1.2 1.2 1.2 1.2 1.1 1.2 1.1 1.2 1.1 1.1 1.1
##  [253] 1.2 1.2 1.1 1.2 1.2 1.2 1.2 1.2 1.2 1.2 1.1 1.2 1.2 1.2 1.1 1.2 1.2 1.1
##  [271] 1.2 1.2 1.1 1.2 1.2 1.2 1.2 1.2 1.2 1.2 1.1 1.2 1.2 1.1 1.1 1.1 1.1 1.1
##  [289] 1.1 1.1 1.1 1.1 1.2 1.1 1.2 1.1 1.1 1.1 1.1 1.1 1.1 1.2 1.2 1.2 1.1 1.1
##  [307] 1.1 1.1 1.1 1.1 1.1 1.1 1.1 1.1 1.1 1.2 1.1 1.1 1.1 1.1 1.2 1.1 1.1 1.1
##  [325] 1.1 1.1 1.1 1.1 1.1 1.1 1.1 1.1 1.1 1.1 1.2 1.2 1.2 1.1 1.1 1.1 1.2 1.1
##  [343] 1.2 1.1 1.1 1.1 1.1 1.1 1.1 1.1 1.1 1.1 1.1 1.1 1.1 1.1 1.1 1.1 1.1 1.1
##  [361] 1.1 1.1 1.1 1.1 1.1 1.1 1.1 1.1 1.1 1.1 1.1 1.1 1.1 1.1 1.2 1.1 1.2 1.2
##  [379] 1.1 1.1 1.1 1.1 1.1 1.1 1.1 1.1 1.1 1.1 1.1 1.1 1.1 1.2 1.2 1.1 1.1 1.2
##  [397] 1.1 1.1 1.1 1.1 1.1 1.1 1.2 1.1 1.1 1.1 1.1 1.1 1.1 1.1 1.1 1.1 1.2 1.2
##  [415] 1.1 1.1 1.1 1.1 1.1 1.1 1.1 1.1 1.1 1.1 1.1 1.1 1.1 1.1 1.1 1.1 1.1 1.1
##  [433] 1.1 1.1 1.1 1.1 1.2 1.1 1.1 1.2 1.1 1.2 1.2 1.1 1.1 1.1 1.2 1.1 1.1 1.1
##  [451] 1.1 1.1 1.1 1.2 1.1 1.1 1.1 1.1 1.1 1.2 1.1 1.1 1.1 1.1 1.1 1.1 1.1 1.1
##  [469] 1.1 1.1 1.1 1.1 1.1 1.1 1.1 1.1 1.1 1.1 1.1 1.2 1.1 1.1 1.1 1.1 1.1 1.1
##  [487] 1.2 1.1 1.1 1.2 1.1 1.1 1.1 1.1 1.1 1.2 1.1 1.2 1.1 1.1 1.2 1.1 1.2 1.1
##  [505] 1.2 1.1 1.1 1.1 1.1 1.1 1.1 1.1 1.1 1.2 1.2 1.1 1.1 1.1 1.1 1.1 1.1 1.1
##  [523] 1.1 1.1 1.1 1.1 1.2 1.2 1.2 1.1 1.1 1.1 1.1 1.2 1.1 1.2 1.1 1.1 1.1 1.1
##  [541] 1.1 1.1 1.1 1.2 1.1 1.1 1.1 1.2 1.2 1.1 1.1 1.1 1.1 1.2 1.1 1.2 1.1 1.1
##  [559] 1.1 1.2 1.1 1.1 1.1 1.1 1.1 1.1 1.1 1.1 1.1 1.1 1.1 1.1 1.1 1.1 1.1 1.2
##  [577] 1.1 1.2 1.1 1.1 1.1 1.1 1.2 1.2 1.1 1.1 1.1 1.1 1.2 1.1 1.1 1.2 1.1 1.1
##  [595] 1.1 1.1 1.1 1.1 1.2 1.1 1.1 1.2 1.1 1.1 1.1 1.1 1.1 1.1 1.1 1.1 1.1 1.1
##  [613] 1.1 1.1 1.1 1.1 1.1 1.1 1.1 1.1 1.1 1.1 1.1 1.2 1.1 1.1 1.1 1.1 1.1 1.2
##  [631] 1.1 1.1 1.1 1.2 1.1 1.2 1.2 1.2 1.1 1.1 1.1 1.1 1.1 1.1 1.1 1.2 1.1 1.1
##  [649] 1.1 1.1 1.1 1.1 1.1 1.1 1.2 1.1 1.1 1.1 1.2 1.1 1.2 1.1 1.1 1.1 1.2 1.1
##  [667] 1.1 1.1 1.1 1.1 1.1 1.1 1.1 1.1 1.1 1.1 1.1 1.1 1.1 1.1 1.1 1.1 1.1 1.1
##  [685] 1.1 1.2 1.1 1.1 1.1 1.1 1.1 1.1 1.2 1.1 1.1 1.2 1.1 1.1 1.1 1.1 1.1 1.1
##  [703] 1.1 1.2 1.1 1.1 1.2 1.1 1.1 1.1 1.1 1.1 1.1 1.2 1.1 1.1 1.1 1.1 1.1 1.1
##  [721] 1.1 1.1 1.1 1.2 1.1 1.1 1.2 1.2 1.1 1.1 1.1 1.1 1.1 1.1 1.1 1.1 1.1 1.1
##  [739] 1.2 1.1 1.1 1.1 1.1 1.1 1.2 1.1 1.1 1.1 1.1 1.1 1.1 1.1 1.1 1.1 1.1 1.1
##  [757] 1.1 1.1 1.1 1.1 1.2 1.1 1.1 1.1 1.1 1.1 1.1 1.1 1.1 1.1 1.2 1.1 1.1 1.1
##  [775] 1.1 1.1 1.1 1.1 1.1 1.1 1.1 1.1 1.1 1.1 1.2 1.1 1.1 1.2 1.1 1.2 1.1 1.1
##  [793] 1.1 1.1 1.1 1.1 1.2 1.1 1.1 1.1 1.1 1.1 1.2 1.1 1.1 1.1 1.2 1.1 1.1 1.1
##  [811] 1.1 1.1 1.1 1.1 1.2 1.1 1.1 1.2 1.2 1.1 1.1 1.1 1.1 1.2 1.1 1.1 1.1 1.1
##  [829] 1.2 1.1 1.1 1.2 1.2 1.2 1.1 1.2 1.1 1.1 1.1 1.1 1.1 1.1 1.1 1.1 1.1 1.1
##  [847] 1.1 1.1 1.1 1.1 1.1 1.1 1.2 1.1 1.1 1.1 1.1 1.1 1.1 1.1 1.2 1.1 1.1 1.2
##  [865] 1.1 1.1 1.1 1.2 1.1 1.1 1.1 1.1 1.2 1.1 1.1 1.1 1.1 1.2 1.1 1.1 1.1 1.1
##  [883] 1.1 1.1 1.1 1.1 1.2 1.1 1.1 1.1 1.1 1.1 1.1 1.1 1.1 1.1 1.1 1.1 1.2 1.1
##  [901] 1.1 1.1 1.2 1.1 1.2 1.1 1.1 1.1 1.1 1.2 1.1 1.1 1.1 1.1 1.2 1.1 1.1 1.1
##  [919] 1.1 1.1 1.1 1.1 1.1 1.1 1.1 1.1 1.1 1.1 1.1 1.2 1.2 1.1 1.1 1.1 1.1 1.1
##  [937] 1.1 1.1 1.1 1.2 1.2 1.2 1.1 1.1 1.1 1.2 1.2 1.1 1.2 1.2 1.1 1.1 1.2 1.1
##  [955] 1.1 1.1 1.1 1.1 1.1 1.2 1.2 1.1 1.1 1.1 1.2 1.1 1.2 1.2 1.2 1.2 1.1 1.1
##  [973] 1.1 1.1 1.1 1.1 1.1 1.1 1.1 1.1 1.1 1.1 1.2 1.2 1.2 1.1 1.1 1.1 1.1 1.2
##  [991] 1.1 1.2 1.1 1.2 1.1 1.1 1.2 1.1 1.1 1.1 1.1 1.2 1.1 1.2 1.1 1.2 1.1 1.2
## [1009] 1.1 1.1 1.2 1.2 1.1 1.1 1.1 1.1 1.1 1.2 1.2 1.2 1.2 1.2 1.2 1.2 1.1 1.2
## [1027] 1.2 1.2 1.1 1.1 1.2 1.1 1.1 1.2 1.1 1.2 1.1 1.1 1.1 1.1 1.1 1.1 1.1 1.1
## [1045] 1.1 1.1 1.1 1.1 1.1 1.1 1.1 1.1 1.2 1.2 1.1 1.1 1.1 1.1 1.1 1.1 1.1 1.1
## [1063] 1.1 1.1 1.1 1.1 1.1 1.2 1.1 1.1 1.1 1.2 1.1 1.1 1.1 1.1 1.1 1.1 1.1 1.1
## [1081] 1.1 1.1 1.2 1.1 1.1 1.2 1.1 1.2 1.1 1.1 1.1 1.1 1.1 1.1 1.2 1.2 1.1 1.1
## [1099] 1.1 1.1 1.1 1.1 1.1 1.1 1.1 1.1 1.1 1.1 1.1 1.2 1.1 1.2 1.1 1.2 1.1 1.1
## [1117] 1.2 1.1 1.2 1.1 1.1 1.2 1.2 1.1 1.1 1.1 1.2 1.1 1.1 1.1 1.2 1.2 1.1 1.1
## [1135] 1.2 1.2 1.2 1.1 1.1 1.1 1.1 1.1 1.1 1.2 1.1 1.1 1.1 1.2 1.1 1.1 1.2 1.2
## [1153] 1.2 1.2 1.1 1.2 1.1 1.1 1.1 1.1 1.1 1.1 1.2 1.2 1.1 1.1 1.1 1.2 1.2 1.1
## [1171] 1.1 1.1 1.1 1.1 1.1 1.2 1.1 1.1 1.1 1.1 1.2 1.2 1.1 1.1 1.1 1.1 1.2 1.1
## [1189] 1.2 1.2 1.1 1.2 1.1 1.1 1.2 1.2 1.1 1.1 1.1 1.1 1.2 1.2 1.1 1.1 1.1 1.1
## [1207] 1.2 1.1 1.2 1.1 1.2 1.1 1.1 1.2 1.1 1.1 1.1 1.1 1.1 1.2 1.2 1.1 1.1 1.1
## [1225] 1.1 1.1 1.1 1.1 1.2 1.2 1.1 1.1 1.1 1.1 1.1 1.2 1.1 1.1 1.1 1.1 1.1 1.2
## [1243] 1.2 1.1 1.2 1.1 1.1 1.1 1.1 1.1 1.1 1.1 1.1 1.1 1.1 1.1 1.1 1.1 1.1 1.1
## [1261] 1.1 1.1 1.2 1.1 1.1 1.2 1.1 1.2 1.2 1.1 1.2 1.1 1.2 1.2 1.1 1.1 1.1 1.1
## [1279] 1.1 1.1 1.1 1.1 1.1 1.1 1.1 1.1 1.2 1.1 1.1 1.1 1.1 1.1 1.1 1.2 1.2 1.1
## [1297] 1.1 1.1 1.2 1.1 1.1 1.2 1.2 1.2 1.1 1.1 1.1 1.1 1.2 1.2 1.1 1.1 1.2 1.2
## [1315] 1.2 1.1 1.1 1.1 1.2 1.2 1.1 1.1 1.1 1.2 1.1 1.1 1.1 1.1 1.1 1.1 1.1 1.2
## [1333] 1.2 1.1 1.1 1.2 1.1 1.1 1.1 1.2 1.1 1.1 1.2 1.1 1.1 1.2 1.2 1.1 1.1 1.2
## [1351] 1.1 1.1 1.1 1.1 1.1 1.1 1.1 1.1 1.1 1.1 1.1 1.1 1.1 1.1 1.1 1.1 1.1 1.1
## [1369] 1.1 1.1 1.1 1.1 1.1 1.1 1.1 1.1 1.1 1.1 1.2 1.2 1.2 1.1 1.2 1.1 1.1 1.1
## [1387] 1.2 1.1 1.1 1.2 1.1 1.1 1.2 1.1 1.1 1.2 1.2 1.1 1.2 1.1 1.1 1.1 1.1 1.1
## [1405] 1.1 1.1 1.1 1.1 1.1 1.1 1.2 1.1 1.2 1.2 1.1 1.1 1.2 1.1 1.2 1.1 1.1 1.1
## [1423] 1.1 1.1 1.1 1.1 1.1 1.2 1.1 1.2 1.1 1.1 1.1 1.1 1.1 1.1 1.1 1.1 1.1 1.1
## [1441] 1.2 1.1 1.1 1.1 1.1 1.1 1.1 1.1 1.2 1.1 1.1 1.1 1.1 1.1 1.1 1.1 1.1 1.2
## [1459] 1.2 1.1 1.1 1.1 1.1 1.1 1.2 1.1 1.1 1.2 1.1 1.1 1.1 1.1 1.1 1.2 1.1 1.1
## [1477] 1.1 1.1 1.1 1.1 1.1 1.1 1.1 1.1 1.2 1.1 1.2 1.1 1.1 1.1 1.1 1.1 1.2 1.1
## [1495] 1.2 1.2 1.1 1.1 1.2 1.2 1.2 1.1 1.2 1.2 1.1 1.1 1.1 1.2 1.2 1.1 1.1 1.1
## [1513] 1.2 1.1 1.2 1.1 1.2 1.2 1.2 1.2 1.2 1.2 1.1 1.2 1.1 1.2 1.1 1.2 1.2 1.1
## [1531] 1.2 1.1 1.2 1.2 1.2 1.2 1.2 1.1 1.1 1.1 1.1 1.1 1.1 1.2 1.2 1.1 1.1 1.1
## [1549] 1.1 1.2 1.2 1.1 1.2 1.2 1.2 1.2 1.1 1.2 1.2 1.2 1.2 1.2 1.1 1.1 1.1 1.2
## [1567] 1.1 1.1 1.1 1.2 1.2 1.1 1.2 1.2 1.2 1.2 1.1 1.1 1.2 1.2 1.2 1.2 1.2 1.2
## [1585] 1.2 1.2 1.2 1.2 1.1 1.1 1.1 1.1 1.1 1.2 1.2 1.1 1.2 1.1 1.2 1.1 1.2 1.2
## [1603] 1.1 1.1 1.1 1.1 1.1 1.1 1.1 1.2 1.2 1.1 1.1 1.1 1.1 1.2 1.1 1.1 1.1 1.2
## [1621] 1.1 1.1 1.2 1.1 1.1 1.1 1.1 1.1 1.1 1.1 1.2 1.1 1.1 1.1 1.2 1.1 1.1 1.1
## [1639] 1.1 1.1 1.1 1.1 1.1 1.1 1.2 1.1 1.1 1.1 1.1 1.1 1.2 1.1 1.2 1.1 1.2 1.1
## [1657] 1.1 1.1 1.1 1.1 1.2 1.1 1.1 1.1 1.1 1.1 1.2 1.2 1.2 1.1 1.2 1.2 1.1 1.2
## [1675] 1.1 1.1 1.1 1.1 1.1 1.1 1.2 1.1 1.1 1.1 1.1 1.1 1.1 1.1 1.2 1.2 1.2 1.2
## [1693] 1.1 1.2 1.1 1.1 1.1 1.1 1.1 1.1 1.1 1.2 1.1 1.2 1.1 1.1 1.1 1.2 1.2 1.1
## [1711] 1.1 1.1 1.1 1.2 1.1 1.2 1.1 1.2 1.1 1.1 1.1 1.1 1.1 1.1 1.2 1.1 1.1 1.1
## [1729] 1.1 1.1 1.1 1.2 1.1 1.2 1.1 1.1 1.1 1.2 1.1 1.1 1.2 1.2 1.2 1.2 1.2 1.2
## [1747] 1.2 1.1 1.1 1.2 1.1 1.1 1.1 1.1 1.1 1.2 1.2 1.1 1.1 1.2 1.1 1.2 1.1 1.1
## [1765] 1.2 1.1 1.2 1.2 1.1 1.2 1.2 1.2 1.2 1.2 1.2 1.1 2.1 2.1 2.2 2.1 2.1 2.1
## [1783] 2.1 2.2 2.1 2.1 2.2 2.1 2.1 2.1 2.1 2.1 2.1 2.1 2.1 2.1 2.1 2.1 2.2 2.2
## [1801] 2.1 2.1 2.2 2.2 2.2 2.2 2.1 2.2 2.2 2.1 2.2 2.1 2.2 2.2 2.2 2.1 2.2 2.2
## [1819] 2.1 2.2 2.2 2.1 2.1 2.2 2.2 2.2 2.2 2.2 2.2 2.2 2.1 2.2 2.2 2.1 2.1 2.1
## [1837] 2.2 2.1 2.2 2.1 2.1 2.1 2.1 2.1 2.1 2.1 2.1 2.1 2.1 2.1 2.2 2.2 2.2 2.1
## [1855] 2.1 2.1 2.1 2.1 2.2 2.1 2.1 2.1 2.1 2.1 2.1 2.2 2.2 2.1 2.2 2.2 2.1 2.1
## [1873] 2.1 2.2 2.2 2.1 2.2 2.2 2.2 2.2 2.2 2.2 2.1 2.2 2.2 2.1 2.2 2.1 2.2 2.2
## [1891] 2.2 2.1 2.1 2.2 2.2 2.1 2.2 2.2 2.2 2.2 2.2 2.2 2.2 2.1 2.2 2.2 2.2 2.1
## [1909] 2.2 2.2 2.1 2.2 2.2 2.2 2.1 2.1 2.2 2.2 2.2 2.2 2.2 2.1 2.1 2.1 2.2 2.1
## [1927] 2.2 2.2 2.1 2.2 2.1 2.1 2.1 2.2 2.2 2.1 2.1 2.1 2.2 2.1 2.2 2.1 2.1 2.1
## [1945] 2.1 2.2 2.2 2.1 2.2 2.1 2.1 2.1 2.1 2.2 2.2 2.2 2.1 2.1 2.2 2.2 2.2 2.2
## [1963] 2.1 2.1 2.1 2.2 2.1 2.2 2.1 2.2 2.1 2.2 2.2 2.2 2.2 2.1 2.1 2.1 2.1 2.1
## [1981] 2.1 2.2 2.1 2.2 2.2 2.1 2.1 2.2 2.1 2.2 2.1 2.2 2.1 2.1 2.1 2.2 2.1 2.1
## [1999] 2.2 2.2 2.1 2.1 2.1 2.1 2.1 2.2 2.1 2.1 2.1 2.2 2.1 2.2 2.2 2.2 2.1 2.1
## [2017] 2.1 2.2 2.2 2.2 2.2 2.2 2.1 2.2 2.1 2.2 2.1 2.1 2.1 2.1 2.2 2.2 2.2 2.1
## [2035] 2.2 2.2 2.2 2.2 2.2 2.2 2.2 2.1 2.2 2.2 2.2 2.1 2.2 2.2 2.1 2.2 2.2 2.1
## [2053] 2.2 2.2 2.2 2.1 2.2 2.2 2.2 2.2 2.1 2.2 2.2 2.1 2.1 2.1 2.1 2.1 2.1 2.1
## [2071] 2.1 2.1 2.2 2.1 2.1 2.1 2.1 2.2 2.2 2.1 2.2 2.1 2.1 2.1 2.1 2.1 2.1 2.1
## [2089] 2.1 2.1 2.1 2.1 2.1 2.2 2.1 2.1 2.1 2.1 2.1 2.2 2.1 2.1 2.1 2.1 2.1 2.1
## [2107] 2.1 2.1 2.1 2.1 2.1 2.1 2.2 2.2 2.2 2.1 2.1 2.1 2.1 2.1 2.2 2.1 2.2 2.1
## [2125] 2.1 2.1 2.1 2.1 2.1 2.1 2.1 2.1 2.1 2.1 2.1 2.1 2.1 2.1 2.1 2.1 2.1 2.1
## [2143] 2.1 2.1 2.1 2.1 2.1 2.1 2.1 2.1 2.1 2.1 2.1 2.1 2.1 2.1 2.2 2.1 2.1 2.1
## [2161] 2.1 2.1 2.1 2.1 2.1 2.1 2.1 2.1 2.1 2.1 2.2 2.2 2.1 2.1 2.2 2.1 2.2 2.1
## [2179] 2.1 2.1 2.1 2.1 2.1 2.1 2.1 2.1 2.1 2.1 2.1 2.1 2.1 2.1 2.1 2.2 2.1 2.1
## [2197] 2.1 2.1 2.1 2.1 2.1 2.1 2.1 2.1 2.1 2.1 2.1 2.1 2.1 2.1 2.1 2.1 2.1 2.1
## [2215] 2.2 2.1 2.1 2.2 2.1 2.2 2.2 2.1 2.1 2.1 2.2 2.1 2.1 2.1 2.1 2.1 2.2 2.1
## [2233] 2.1 2.1 2.1 2.1 2.1 2.1 2.1 2.1 2.1 2.1 2.1 2.1 2.1 2.1 2.1 2.1 2.1 2.1
## [2251] 2.1 2.1 2.1 2.1 2.1 2.1 2.2 2.1 2.1 2.1 2.1 2.1 2.1 2.2 2.1 2.1 2.2 2.1
## [2269] 2.1 2.1 2.1 2.1 2.2 2.1 2.2 2.1 2.1 2.2 2.1 2.1 2.2 2.1 2.2 2.1 2.1 2.1
## [2287] 2.1 2.1 2.1 2.1 2.1 2.1 2.2 2.2 2.1 2.1 2.1 2.1 2.1 2.1 2.1 2.1 2.1 2.1
## [2305] 2.1 2.2 2.2 2.2 2.1 2.1 2.1 2.1 2.2 2.1 2.2 2.1 2.1 2.1 2.1 2.1 2.1 2.1
## [2323] 2.2 2.1 2.1 2.1 2.2 2.1 2.1 2.1 2.1 2.1 2.2 2.1 2.2 2.1 2.1 2.1 2.1 2.1
## [2341] 2.1 2.1 2.1 2.1 2.1 2.1 2.1 2.1 2.1 2.1 2.1 2.1 2.1 2.2 2.1 2.1 2.1 2.1
## [2359] 2.1 2.2 2.2 2.1 2.1 2.1 2.1 2.2 2.1 2.1 2.2 2.1 2.1 2.1 2.1 2.1 2.1 2.2
## [2377] 2.1 2.1 2.2 2.1 2.1 2.1 2.1 2.1 2.1 2.1 2.1 2.1 2.1 2.1 2.1 2.1 2.1 2.1
## [2395] 2.1 2.1 2.1 2.1 2.2 2.1 2.1 2.1 2.1 2.1 2.2 2.1 2.1 2.1 2.2 2.1 2.2 2.2
## [2413] 2.2 2.1 2.1 2.1 2.1 2.1 2.1 2.1 2.2 2.1 2.1 2.1 2.1 2.1 2.1 2.1 2.2 2.1
## [2431] 2.1 2.1 2.2 2.1 2.2 2.1 2.1 2.1 2.2 2.1 2.1 2.1 2.1 2.1 2.1 2.1 2.1 2.1
## [2449] 2.1 2.1 2.1 2.1 2.1 2.1 2.1 2.1 2.1 2.1 2.1 2.2 2.1 2.1 2.1 2.1 2.1 2.1
## [2467] 2.2 2.1 2.1 2.2 2.1 2.1 2.1 2.1 2.1 2.1 2.2 2.1 2.1 2.2 2.1 2.1 2.1 2.1
## [2485] 2.1 2.1 2.1 2.2 2.1 2.1 2.1 2.1 2.1 2.1 2.1 2.1 2.1 2.1 2.1 2.1 2.2 2.1
## [2503] 2.2 2.1 2.1 2.1 2.1 2.1 2.1 2.1 2.1 2.1 2.1 2.1 2.1 2.1 2.1 2.1 2.2 2.1
## [2521] 2.1 2.1 2.1 2.1 2.1 2.1 2.1 2.1 2.1 2.1 2.1 2.1 2.1 2.1 2.1 2.2 2.1 2.1
## [2539] 2.1 2.1 2.1 2.1 2.1 2.1 2.1 2.2 2.1 2.1 2.1 2.1 2.1 2.1 2.1 2.1 2.1 2.1
## [2557] 2.1 2.1 2.1 2.2 2.1 2.1 2.2 2.1 2.2 2.1 2.1 2.1 2.1 2.1 2.1 2.2 2.1 2.1
## [2575] 2.1 2.1 2.1 2.1 2.1 2.2 2.1 2.1 2.1 2.1 2.1 2.1 2.1 2.1 2.2 2.1 2.1 2.2
## [2593] 2.2 2.1 2.1 2.2 2.1 2.1 2.1 2.1 2.2 2.1 2.1 2.2 2.2 2.2 2.2 2.1 2.2 2.1
## [2611] 2.1 2.1 2.1 2.1 2.1 2.1 2.1 2.1 2.1 2.1 2.1 2.1 2.1 2.1 2.2 2.1 2.2 2.1
## [2629] 2.1 2.1 2.1 2.1 2.1 2.2 2.1 2.1 2.2 2.1 2.1 2.2 2.1 2.1 2.1 2.1 2.2 2.1
## [2647] 2.1 2.1 2.1 2.1 2.1 2.1 2.1 2.1 2.1 2.1 2.1 2.1 2.2 2.1 2.1 2.1 2.1 2.1
## [2665] 2.1 2.2 2.1 2.1 2.1 2.2 2.1 2.1 2.1 2.2 2.1 2.2 2.1 2.1 2.1 2.1 2.1 2.2
## [2683] 2.1 2.1 2.1 2.1 2.1 2.1 2.1 2.1 2.1 2.1 2.1 2.1 2.1 2.1 2.1 2.1 2.1 2.2
## [2701] 2.2 2.1 2.1 2.1 2.1 2.1 2.1 2.1 2.1 2.2 2.2 2.2 2.1 2.1 2.1 2.2 2.2 2.1
## [2719] 2.2 2.2 2.1 2.1 2.2 2.1 2.1 2.1 2.1 2.1 2.1 2.2 2.2 2.1 2.1 2.1 2.2 2.1
## [2737] 2.2 2.2 2.1 2.2 2.2 2.1 2.2 2.1 2.1 2.1 2.1 2.1 2.1 2.1 2.1 2.1 2.2 2.2
## [2755] 2.2 2.1 2.1 2.1 2.2 2.1 2.2 2.1 2.2 2.1 2.2 2.1 2.1 2.2 2.1 2.1 2.1 2.2
## [2773] 2.2 2.1 2.2 2.2 2.1 2.1 2.1 2.2 2.2 2.1 2.1 2.1 2.1 2.1 2.1 2.2 2.2 2.2
## [2791] 2.2 2.2 2.1 2.2 2.2 2.1 2.1 2.2 2.1 2.1 2.1 2.2 2.1 2.2 2.1 2.1 2.1 2.1
## [2809] 2.1 2.1 2.1 2.1 2.1 2.1 2.1 2.1 2.1 2.1 2.1 2.2 2.2 2.1 2.1 2.1 2.1 2.1
## [2827] 2.1 2.1 2.1 2.1 2.1 2.1 2.1 2.1 2.1 2.2 2.1 2.1 2.1 2.2 2.1 2.1 2.1 2.1
## [2845] 2.1 2.1 2.1 2.1 2.1 2.1 2.2 2.1 2.1 2.1 2.2 2.1 2.1 2.1 2.1 2.1 2.1 2.2
## [2863] 2.2 2.1 2.1 2.1 2.1 2.1 2.1 2.1 2.1 2.1 2.1 2.1 2.1 2.1 2.1 2.1 2.1 2.2
## [2881] 2.1 2.2 2.1 2.2 2.1 2.1 2.2 2.1 2.1 2.1 2.2 2.2 2.1 2.1 2.1 2.2 2.1 2.1
## [2899] 2.1 2.2 2.1 2.1 2.2 2.2 2.1 2.2 2.1 2.1 2.1 2.1 2.1 2.1 2.1 2.1 2.1 2.2
## [2917] 2.2 2.1 2.1 2.1 2.1 2.1 2.2 2.1 2.2 2.2 2.1 2.1 2.1 2.2 2.2 2.1 2.1 2.1
## [2935] 2.1 2.1 2.1 2.1 2.1 2.1 2.2 2.2 2.1 2.1 2.1 2.1 2.2 2.2 2.2 2.1 2.1 2.2
## [2953] 2.1 2.1 2.1 2.1 2.2 2.1 2.1 2.1 2.1 2.2 2.2 2.1 2.1 2.2 2.1 2.1 2.1 2.1
## [2971] 2.1 2.2 2.1 2.1 2.1 2.1 2.1 2.1 2.2 2.2 2.1 2.1 2.1 2.1 2.2 2.1 2.1 2.1
## [2989] 2.1 2.2 2.2 2.1 2.2 2.1 2.1 2.1 2.1 2.1 2.1 2.1 2.1 2.1 2.1 2.1 2.1 2.2
## [3007] 2.1 2.1 2.2 2.1 2.2 2.2 2.2 2.1 2.1 2.1 2.1 2.1 2.1 2.1 2.1 2.1 2.1 2.1
## [3025] 2.1 2.1 2.1 2.1 2.1 2.1 2.1 2.1 2.1 2.2 2.2 2.1 2.1 2.1 2.2 2.1 2.1 2.1
## [3043] 2.2 2.2 2.1 2.1 2.1 2.1 2.1 2.2 2.2 2.1 2.1 2.1 2.2 2.2 2.2 2.1 2.1 2.2
## [3061] 2.1 2.1 2.1 2.2 2.1 2.1 2.1 2.1 2.1 2.1 2.1 2.2 2.2 2.1 2.1 2.1 2.2 2.1
## [3079] 2.1 2.2 2.1 2.1 2.2 2.1 2.1 2.1 2.2 2.1 2.1 2.1 2.1 2.1 2.1 2.1 2.1 2.1
## [3097] 2.1 2.1 2.1 2.1 2.1 2.1 2.1 2.1 2.1 2.1 2.1 2.1 2.1 2.1 2.1 2.2 2.1 2.1
## [3115] 2.1 2.1 2.2 2.1 2.1 2.2 2.1 2.1 2.2 2.1 2.1 2.2 2.2 2.1 2.2 2.2 2.1 2.1
## [3133] 2.1 2.1 2.1 2.1 2.1 2.1 2.2 2.1 2.2 2.2 2.1 2.1 2.2 2.1 2.1 2.1 2.1 2.1
## [3151] 2.1 2.1 2.1 2.2 2.1 2.1 2.1 2.1 2.1 2.1 2.1 2.1 2.1 2.1 2.2 2.1 2.1 2.1
## [3169] 2.1 2.1 2.1 2.2 2.1 2.1 2.1 2.1 2.1 2.1 2.2 2.1 2.1 2.1 2.1 2.2 2.1 2.2
## [3187] 2.1 2.1 2.1 2.1 2.1 2.2 2.1 2.1 2.1 2.1 2.1 2.1 2.1 2.1 2.2 2.2 2.1 2.2
## [3205] 2.1 2.1 2.1 2.1 2.1 2.2 2.2 2.2 2.1 2.2 2.1 2.2 2.2 2.1 2.1 2.2 2.2 2.1
## [3223] 2.1 2.1 2.2 2.1 2.2 2.1 2.2 2.2 2.2 2.2 2.1 2.2 2.1 2.2 2.1 2.2 2.1 2.2
## [3241] 2.2 2.2 2.1 2.2 2.1 2.2 2.2 2.2 2.2 2.2 2.1 2.1 2.1 2.1 2.2 2.1 2.1 2.1
## [3259] 2.2 2.2 2.1 2.1 2.1 2.1 2.2 2.2 2.1 2.2 2.2 2.2 2.1 2.2 2.2 2.2 2.2 2.1
## [3277] 2.1 2.1 2.2 2.1 2.1 2.1 2.2 2.2 2.1 2.2 2.2 2.2 2.2 2.1 2.1 2.2 2.2 2.2
## [3295] 2.2 2.2 2.2 2.2 2.2 2.1 2.1 2.1 2.1 2.1 2.2 2.2 2.1 2.2 2.2 2.1 2.2 2.2
## [3313] 2.1 2.1 2.1 2.1 2.1 2.1 2.2 2.1 2.1 2.2 2.1 2.1 2.2 2.1 2.1 2.2 2.1 2.1
## [3331] 2.2 2.1 2.1 2.1 2.1 2.1 2.1 2.1 2.1 2.1 2.2 2.1 2.1 2.1 2.2 2.1 2.1 2.1
## [3349] 2.1 2.1 2.1 2.1 2.1 2.2 2.1 2.2 2.1 2.1 2.1 2.2 2.1 2.1 2.2 2.1 2.1 2.1
## [3367] 2.1 2.1 2.1 2.1 2.1 2.2 2.2 2.1 2.2 2.1 2.2 2.1 2.1 2.1 2.1 2.1 2.2 2.1
## [3385] 2.1 2.1 2.1 2.1 2.2 2.2 2.2 2.2 2.1 2.1 2.2 2.1 2.1 2.1 2.1 2.1 2.2 2.2
## [3403] 2.1 2.1 2.1 2.2 2.2 2.1 2.1 2.1 2.2 2.1 2.2 2.1 2.2 2.1 2.1 2.1 2.1 2.1
## [3421] 2.1 2.1 2.1 2.1 2.1 2.1 2.2 2.1 2.2 2.1 2.1 2.2 2.2 2.1 2.2 2.2 2.2 2.2
## [3439] 2.2 2.2 2.2 2.1 2.1 2.2 2.1 2.2 2.1 2.2 2.2 2.1 2.1 2.2 2.1 2.2 2.1 2.1
## [3457] 2.2 2.1 2.2 2.2 2.1 2.1 2.2 2.2 2.2 2.2 2.2 3.2 3.1 3.1 3.2 3.1 3.1 3.2
## [3475] 3.1 3.1 3.1 3.2 3.2 3.1 3.1 3.1 3.1 3.1 3.1 3.1 3.1 3.2 3.2 3.2 3.1 3.2
## [3493] 3.1 3.1 3.2 3.2 3.1 3.1 3.2 3.2 3.2 3.1 3.2 3.1 3.1 3.2 3.2 3.2 3.2 3.2
## [3511] 3.1 3.2 3.2 3.1 3.1 3.2 3.1 3.1 3.1 3.1 3.1 3.1 3.2 3.1 3.1 3.1 3.1 3.2
## [3529] 3.2 3.2 3.1 3.1 3.1 3.1 3.1 3.2 3.1 3.1 3.1 3.1 3.1 3.2 3.2 3.1 3.2 3.2
## [3547] 3.1 3.1 3.2 3.2 3.2 3.2 3.2 3.2 3.1 3.2 3.1 3.1 3.1 3.2 3.2 3.2 3.2 3.1
## [3565] 3.2 3.2 3.2 3.2 3.2 3.2 3.2 3.1 3.2 3.2 3.2 3.2 3.2 3.2 3.1 3.1 3.2 3.2
## [3583] 3.2 3.2 3.2 3.1 3.1 3.1 3.2 3.2 3.2 3.1 3.1 3.1 3.2 3.2 3.1 3.1 3.1 3.1
## [3601] 3.2 3.1 3.2 3.1 3.1 3.1 3.1 3.2 3.2 3.2 3.1 3.1 3.1 3.1 3.2 3.2 3.2 3.1
## [3619] 3.1 3.2 3.2 3.2 3.2 3.1 3.2 3.1 3.2 3.2 3.2 3.1 3.2 3.2 3.1 3.1 3.1 3.2
## [3637] 3.1 3.1 3.2 3.2 3.1 3.2 3.1 3.1 3.1 3.2 3.1 3.1 3.2 3.2 3.1 3.1 3.2 3.1
## [3655] 3.1 3.2 3.1 3.1 3.2 3.1 3.2 3.2 3.2 3.1 3.1 3.1 3.2 3.2 3.2 3.2 3.1 3.2
## [3673] 3.1 3.1 3.1 3.2 3.2 3.2 3.2 3.2 3.2 3.2 3.2 3.2 3.2 3.2 3.1 3.2 3.2 3.1
## [3691] 3.2 3.2 3.2 3.1 3.2 3.2 3.2 3.2 3.1 3.2 3.2 3.2 3.1 3.1 3.1 3.1 3.1 3.1
## [3709] 3.1 3.1 3.2 3.1 3.1 3.1 3.1 3.2 3.2 3.2 3.1 3.1 3.1 3.1 3.1 3.1 3.1 3.1
## [3727] 3.2 3.1 3.1 3.1 3.2 3.1 3.1 3.1 3.1 3.1 3.1 3.1 3.1 3.1 3.2 3.2 3.2 3.1
## [3745] 3.1 3.1 3.1 3.1 3.2 3.1 3.2 3.1 3.1 3.1 3.1 3.1 3.1 3.1 3.1 3.1 3.1 3.1
## [3763] 3.1 3.1 3.1 3.1 3.1 3.1 3.1 3.1 3.1 3.1 3.1 3.1 3.1 3.1 3.1 3.1 3.1 3.2
## [3781] 3.1 3.1 3.1 3.1 3.1 3.1 3.1 3.1 3.1 3.1 3.2 3.2 3.1 3.1 3.1 3.1 3.1 3.1
## [3799] 3.1 3.2 3.1 3.1 3.1 3.1 3.1 3.1 3.1 3.1 3.1 3.1 3.2 3.2 3.1 3.1 3.1 3.1
## [3817] 3.1 3.1 3.1 3.1 3.1 3.1 3.1 3.1 3.1 3.1 3.1 3.1 3.1 3.1 3.1 3.1 3.2 3.1
## [3835] 3.1 3.1 3.2 3.1 3.1 3.1 3.1 3.2 3.1 3.1 3.1 3.1 3.1 3.2 3.1 3.1 3.1 3.1
## [3853] 3.1 3.1 3.1 3.1 3.1 3.1 3.1 3.1 3.1 3.2 3.1 3.1 3.1 3.1 3.1 3.1 3.2 3.1
## [3871] 3.1 3.2 3.1 3.1 3.1 3.1 3.1 3.2 3.1 3.2 3.1 3.1 3.2 3.1 3.1 3.2 3.1 3.2
## [3889] 3.1 3.1 3.1 3.1 3.1 3.1 3.1 3.1 3.2 3.1 3.1 3.1 3.1 3.1 3.1 3.1 3.1 3.1
## [3907] 3.1 3.2 3.2 3.2 3.1 3.1 3.1 3.2 3.1 3.2 3.1 3.1 3.1 3.1 3.1 3.2 3.1 3.1
## [3925] 3.2 3.1 3.2 3.2 3.1 3.1 3.1 3.1 3.2 3.1 3.2 3.1 3.1 3.2 3.1 3.1 3.1 3.1
## [3943] 3.1 3.1 3.1 3.1 3.1 3.1 3.2 3.1 3.2 3.1 3.1 3.1 3.2 3.1 3.1 3.1 3.2 3.1
## [3961] 3.1 3.2 3.1 3.1 3.1 3.1 3.1 3.2 3.1 3.1 3.2 3.1 3.1 3.1 3.1 3.1 3.1 3.1
## [3979] 3.1 3.1 3.1 3.1 3.1 3.1 3.1 3.1 3.1 3.1 3.1 3.1 3.2 3.1 3.1 3.1 3.1 3.1
## [3997] 3.1 3.1 3.1 3.2 3.1 3.2 3.2 3.2 3.1 3.1 3.1 3.1 3.1 3.1 3.1 3.2 3.1 3.1
## [4015] 3.1 3.1 3.1 3.1 3.1 3.1 3.1 3.1 3.1 3.2 3.1 3.2 3.1 3.1 3.1 3.1 3.1 3.1
## [4033] 3.1 3.1 3.1 3.1 3.1 3.1 3.1 3.1 3.1 3.1 3.1 3.2 3.1 3.1 3.1 3.1 3.1 3.1
## [4051] 3.1 3.1 3.1 3.1 3.2 3.1 3.1 3.2 3.1 3.1 3.1 3.1 3.2 3.1 3.1 3.1 3.1 3.1
## [4069] 3.1 3.1 3.1 3.1 3.2 3.1 3.1 3.2 3.1 3.1 3.1 3.1 3.1 3.1 3.1 3.1 3.1 3.1
## [4087] 3.1 3.2 3.1 3.1 3.1 3.1 3.1 3.1 3.1 3.1 3.1 3.1 3.1 3.2 3.1 3.1 3.1 3.1
## [4105] 3.1 3.1 3.1 3.2 3.1 3.1 3.1 3.1 3.1 3.1 3.1 3.1 3.1 3.1 3.2 3.1 3.1 3.2
## [4123] 3.1 3.2 3.1 3.1 3.1 3.2 3.1 3.1 3.1 3.1 3.1 3.1 3.1 3.1 3.1 3.2 3.1 3.2
## [4141] 3.1 3.1 3.1 3.1 3.2 3.1 3.1 3.1 3.1 3.2 3.1 3.1 3.2 3.2 3.2 3.1 3.2 3.1
## [4159] 3.1 3.1 3.1 3.1 3.1 3.1 3.1 3.1 3.1 3.1 3.2 3.1 3.2 3.1 3.1 3.1 3.1 3.1
## [4177] 3.1 3.2 3.1 3.1 3.2 3.1 3.1 3.1 3.2 3.1 3.1 3.1 3.2 3.1 3.1 3.1 3.1 3.1
## [4195] 3.2 3.1 3.1 3.1 3.1 3.1 3.1 3.1 3.1 3.2 3.1 3.1 3.1 3.1 3.1 3.1 3.1 3.2
## [4213] 3.1 3.1 3.2 3.1 3.1 3.2 3.2 3.1 3.1 3.1 3.1 3.1 3.2 3.1 3.1 3.1 3.1 3.2
## [4231] 3.1 3.1 3.1 3.1 3.1 3.1 3.1 3.1 3.1 3.1 3.1 3.2 3.2 3.1 3.1 3.1 3.1 3.1
## [4249] 3.1 3.1 3.2 3.2 3.2 3.1 3.2 3.2 3.1 3.2 3.2 3.1 3.1 3.2 3.1 3.1 3.1 3.1
## [4267] 3.1 3.1 3.2 3.2 3.1 3.2 3.1 3.2 3.2 3.2 3.2 3.2 3.1 3.1 3.1 3.1 3.1 3.1
## [4285] 3.1 3.1 3.2 3.2 3.2 3.1 3.1 3.1 3.1 3.2 3.1 3.2 3.1 3.2 3.1 3.1 3.1 3.1
## [4303] 3.2 3.1 3.2 3.1 3.1 3.2 3.1 3.1 3.1 3.2 3.1 3.1 3.1 3.1 3.1 3.1 3.2 3.2
## [4321] 3.2 3.2 3.2 3.2 3.2 3.2 3.1 3.2 3.2 3.1 3.2 3.1 3.2 3.1 3.1 3.1 3.1 3.1
## [4339] 3.1 3.1 3.1 3.1 3.1 3.1 3.1 3.1 3.1 3.1 3.1 3.1 3.1 3.2 3.2 3.1 3.1 3.1
## [4357] 3.1 3.1 3.1 3.1 3.2 3.1 3.1 3.1 3.1 3.1 3.1 3.1 3.1 3.1 3.2 3.1 3.1 3.2
## [4375] 3.1 3.2 3.1 3.1 3.2 3.2 3.1 3.1 3.1 3.1 3.1 3.1 3.1 3.1 3.1 3.1 3.1 3.2
## [4393] 3.1 3.1 3.2 3.1 3.1 3.2 3.1 3.1 3.2 3.1 3.1 3.1 3.2 3.2 3.1 3.2 3.1 3.1
## [4411] 3.1 3.2 3.1 3.1 3.2 3.1 3.2 3.1 3.1 3.1 3.1 3.1 3.1 3.2 3.2 3.1 3.2 3.2
## [4429] 3.2 3.1 3.2 3.1 3.1 3.1 3.1 3.1 3.1 3.1 3.2 3.1 3.2 3.2 3.2 3.1 3.1 3.1
## [4447] 3.1 3.2 3.2 3.2 3.1 3.1 3.2 3.2 3.1 3.1 3.1 3.1 3.1 3.2 3.1 3.1 3.1 3.2
## [4465] 3.1 3.1 3.1 3.1 3.2 3.2 3.1 3.1 3.1 3.1 3.1 3.1 3.1 3.1 3.1 3.1 3.1 3.1
## [4483] 3.1 3.2 3.1 3.2 3.2 3.1 3.1 3.1 3.1 3.1 3.1 3.1 3.1 3.2 3.1 3.1 3.1 3.1
## [4501] 3.2 3.1 3.1 3.1 3.1 3.1 3.1 3.1 3.2 3.1 3.2 3.2 3.1 3.1 3.1 3.1 3.1 3.1
## [4519] 3.2 3.2 3.1 3.1 3.1 3.2 3.1 3.1 3.1 3.2 3.2 3.1 3.1 3.1 3.2 3.1 3.1 3.1
## [4537] 3.1 3.1 3.1 3.1 3.1 3.1 3.1 3.1 3.1 3.1 3.1 3.1 3.2 3.2 3.2 3.2 3.1 3.1
## [4555] 3.2 3.1 3.2 3.2 3.1 3.1 3.1 3.1 3.1 3.1 3.2 3.2 3.1 3.1 3.1 3.1 3.2 3.2
## [4573] 3.1 3.1 3.1 3.1 3.1 3.1 3.1 3.1 3.1 3.1 3.1 3.1 3.1 3.1 3.1 3.1 3.2 3.1
## [4591] 3.1 3.1 3.1 3.1 3.1 3.1 3.1 3.1 3.1 3.1 3.1 3.2 3.1 3.2 3.1 3.2 3.1 3.1
## [4609] 3.1 3.1 3.1 3.1 3.2 3.2 3.2 3.1 3.1 3.1 3.2 3.1 3.1 3.2 3.2 3.1 3.2 3.2
## [4627] 3.1 3.2 3.1 3.2 3.2 3.1 3.1 3.1 3.2 3.1 3.2 3.2 3.2 3.2 3.2 3.2 3.1 3.2
## [4645] 3.2 3.2 3.1 3.2 3.2 3.2 3.2 3.2 3.2 3.2 3.2 3.2 3.2 3.2 3.1 3.1 3.1 3.1
## [4663] 3.1 3.2 3.2 3.1 3.1 3.1 3.1 3.1 3.2 3.2 3.1 3.1 3.1 3.1 3.1 3.1 3.1 3.2
## [4681] 3.1 3.2 3.1 3.1 3.1 3.1 3.1 3.1 3.1 3.2 3.2 3.1 3.1 3.1 3.2 3.1 3.1 3.2
## [4699] 3.1 3.1 3.2 3.1 3.1 3.1 3.1 3.2 3.2 3.2 3.2 3.1 3.1 3.1 3.1 3.2 3.1 3.1
## [4717] 3.1 3.1 3.1 3.2 3.1 3.1 3.1 3.1 3.1 3.1 3.2 3.1 3.1 3.1 3.1 3.2 3.2 3.1
## [4735] 3.1 3.1 3.1 3.2 3.1 3.2 3.2 3.1 3.1 3.1 3.1 3.1 3.2 3.1 3.1 3.1 3.1 3.2
## [4753] 3.2 3.2 3.1 3.2 3.2 3.2 3.1 3.1 3.1 3.2 3.2 3.1 3.1 3.1 3.2 3.2 3.1 3.1
## [4771] 3.2 3.1 3.1 3.2 3.2 3.1 4.1 4.1 4.2 4.1 4.1 4.1 4.1 4.1 4.1 4.1 4.2 4.2
## [4789] 4.2 4.1 4.1 4.1 4.1 4.1 4.2 4.1 4.1 4.1 4.1 4.2 4.1 4.1 4.1 4.2 4.1 4.2
## [4807] 4.1 4.2 4.2 4.1 4.2 4.2 4.2 4.2 4.1 4.2 4.1 4.2 4.1 4.1 4.2 4.2 4.2 4.2
## [4825] 4.2 4.2 4.2 4.2 4.1 4.1 4.1 4.2 4.1 4.1 4.1 4.1 4.1 4.1 4.1 4.2 4.1 4.1
## [4843] 4.1 4.1 4.1 4.2 4.2 4.2 4.1 4.1 4.1 4.1 4.1 4.2 4.1 4.1 4.1 4.1 4.1 4.1
## [4861] 4.2 4.1 4.2 4.2 4.1 4.1 4.2 4.2 4.2 4.2 4.2 4.1 4.2 4.1 4.1 4.1 4.2 4.2
## [4879] 4.2 4.2 4.1 4.2 4.2 4.2 4.2 4.2 4.2 4.2 4.1 4.2 4.2 4.2 4.2 4.2 4.1 4.2
## [4897] 4.2 4.1 4.1 4.2 4.2 4.2 4.2 4.2 4.2 4.1 4.1 4.1 4.2 4.2 4.2 4.1 4.1 4.1
## [4915] 4.2 4.2 4.1 4.1 4.1 4.2 4.1 4.2 4.1 4.1 4.1 4.1 4.2 4.2 4.1 4.2 4.1 4.1
## [4933] 4.1 4.1 4.2 4.2 4.2 4.1 4.1 4.2 4.2 4.2 4.2 4.2 4.1 4.1 4.2 4.1 4.2 4.1
## [4951] 4.2 4.2 4.1 4.2 4.2 4.2 4.2 4.1 4.1 4.1 4.1 4.1 4.1 4.2 4.1 4.1 4.2 4.1
## [4969] 4.2 4.1 4.2 4.1 4.1 4.1 4.2 4.2 4.1 4.2 4.2 4.1 4.1 4.2 4.1 4.1 4.2 4.1
## [4987] 4.1 4.1 4.2 4.1 4.2 4.2 4.2 4.1 4.1 4.1 4.2 4.2 4.2 4.2 4.2 4.1 4.2 4.1
## [5005] 4.1 4.1 4.1 4.2 4.2 4.2 4.2 4.2 4.2 4.2 4.1 4.2 4.2 4.2 4.2 4.2 4.2 4.1
## [5023] 4.2 4.2 4.1 4.2 4.2 4.2 4.1 4.2 4.2 4.2 4.2 4.1 4.2 4.2 4.2 4.1 4.1 4.1
## [5041] 4.1 4.1 4.1 4.1 4.1 4.2 4.1 4.1 4.1 4.1 4.1 4.1 4.2 4.2 4.1 4.2 4.1 4.1
## [5059] 4.1 4.1 4.1 4.1 4.1 4.2 4.1 4.1 4.1 4.1 4.1 4.2 4.1 4.1 4.1 4.1 4.1 4.1
## [5077] 4.1 4.1 4.1 4.1 4.1 4.2 4.2 4.2 4.1 4.1 4.1 4.1 4.1 4.2 4.1 4.2 4.1 4.1
## [5095] 4.1 4.1 4.1 4.1 4.1 4.1 4.1 4.1 4.1 4.1 4.1 4.1 4.1 4.1 4.1 4.1 4.1 4.1
## [5113] 4.1 4.1 4.1 4.1 4.1 4.1 4.1 4.1 4.1 4.1 4.2 4.1 4.2 4.2 4.1 4.1 4.1 4.1
## [5131] 4.1 4.1 4.1 4.1 4.1 4.1 4.1 4.1 4.2 4.2 4.1 4.1 4.1 4.2 4.1 4.1 4.1 4.1
## [5149] 4.1 4.1 4.2 4.1 4.1 4.1 4.1 4.1 4.1 4.1 4.1 4.1 4.1 4.2 4.1 4.1 4.1 4.1
## [5167] 4.1 4.1 4.1 4.1 4.1 4.1 4.1 4.1 4.1 4.1 4.1 4.1 4.1 4.1 4.1 4.1 4.2 4.1
## [5185] 4.2 4.1 4.1 4.1 4.2 4.1 4.1 4.1 4.1 4.1 4.2 4.1 4.1 4.1 4.1 4.2 4.1 4.1
## [5203] 4.1 4.1 4.1 4.1 4.1 4.1 4.1 4.1 4.1 4.1 4.1 4.1 4.1 4.1 4.1 4.2 4.1 4.1
## [5221] 4.1 4.1 4.1 4.1 4.2 4.1 4.1 4.2 4.1 4.1 4.1 4.1 4.1 4.2 4.1 4.2 4.1 4.1
## [5239] 4.2 4.1 4.1 4.2 4.1 4.2 4.1 4.1 4.1 4.1 4.1 4.1 4.1 4.1 4.2 4.1 4.1 4.1
## [5257] 4.1 4.1 4.1 4.1 4.1 4.1 4.1 4.2 4.2 4.1 4.1 4.1 4.2 4.1 4.2 4.1 4.1 4.1
## [5275] 4.1 4.1 4.2 4.1 4.1 4.1 4.2 4.2 4.2 4.1 4.1 4.1 4.1 4.2 4.1 4.2 4.1 4.1
## [5293] 4.1 4.2 4.1 4.1 4.1 4.1 4.1 4.1 4.1 4.1 4.1 4.1 4.1 4.2 4.1 4.2 4.1 4.1
## [5311] 4.1 4.1 4.1 4.1 4.1 4.1 4.2 4.1 4.1 4.2 4.1 4.1 4.1 4.1 4.1 4.2 4.1 4.1
## [5329] 4.2 4.1 4.1 4.1 4.1 4.1 4.1 4.1 4.1 4.1 4.1 4.1 4.1 4.1 4.1 4.1 4.1 4.1
## [5347] 4.1 4.1 4.1 4.1 4.1 4.2 4.1 4.1 4.1 4.1 4.1 4.2 4.1 4.1 4.1 4.2 4.1 4.2
## [5365] 4.2 4.2 4.1 4.1 4.1 4.1 4.1 4.1 4.1 4.2 4.1 4.1 4.1 4.1 4.1 4.1 4.1 4.1
## [5383] 4.1 4.1 4.1 4.1 4.2 4.1 4.2 4.1 4.2 4.1 4.1 4.1 4.1 4.1 4.1 4.1 4.1 4.1
## [5401] 4.1 4.1 4.1 4.1 4.1 4.1 4.1 4.1 4.1 4.1 4.2 4.1 4.1 4.1 4.1 4.1 4.1 4.1
## [5419] 4.1 4.1 4.1 4.1 4.1 4.1 4.2 4.1 4.1 4.2 4.1 4.1 4.1 4.1 4.1 4.2 4.1 4.1
## [5437] 4.1 4.1 4.1 4.1 4.1 4.1 4.2 4.1 4.1 4.1 4.2 4.1 4.1 4.1 4.1 4.1 4.1 4.1
## [5455] 4.1 4.1 4.1 4.1 4.1 4.1 4.2 4.1 4.1 4.1 4.1 4.1 4.1 4.1 4.1 4.1 4.1 4.1
## [5473] 4.1 4.1 4.1 4.1 4.1 4.2 4.1 4.1 4.1 4.1 4.1 4.1 4.2 4.1 4.1 4.1 4.1 4.1
## [5491] 4.1 4.1 4.1 4.1 4.2 4.1 4.1 4.2 4.1 4.2 4.1 4.1 4.1 4.1 4.1 4.1 4.1 4.1
## [5509] 4.1 4.2 4.1 4.1 4.1 4.2 4.1 4.1 4.1 4.1 4.1 4.1 4.2 4.1 4.1 4.2 4.2 4.1
## [5527] 4.1 4.1 4.1 4.2 4.1 4.1 4.1 4.1 4.2 4.1 4.1 4.2 4.2 4.2 4.2 4.1 4.2 4.1
## [5545] 4.1 4.1 4.1 4.1 4.1 4.1 4.1 4.1 4.1 4.1 4.1 4.1 4.2 4.1 4.2 4.1 4.1 4.1
## [5563] 4.1 4.1 4.2 4.1 4.1 4.2 4.1 4.1 4.1 4.2 4.1 4.1 4.1 4.2 4.1 4.1 4.1 4.1
## [5581] 4.1 4.2 4.1 4.1 4.1 4.1 4.1 4.1 4.1 4.1 4.1 4.2 4.1 4.1 4.1 4.1 4.1 4.1
## [5599] 4.1 4.1 4.2 4.1 4.1 4.1 4.2 4.1 4.2 4.2 4.1 4.1 4.1 4.1 4.2 4.1 4.1 4.1
## [5617] 4.1 4.2 4.1 4.1 4.1 4.1 4.1 4.1 4.1 4.1 4.1 4.1 4.1 4.1 4.1 4.2 4.2 4.1
## [5635] 4.1 4.1 4.1 4.1 4.1 4.1 4.2 4.2 4.2 4.1 4.1 4.2 4.2 4.1 4.2 4.1 4.1 4.1
## [5653] 4.1 4.1 4.1 4.1 4.1 4.2 4.1 4.1 4.2 4.1 4.2 4.2 4.2 4.2 4.2 4.1 4.1 4.1
## [5671] 4.1 4.1 4.1 4.1 4.1 4.1 4.2 4.2 4.2 4.1 4.1 4.1 4.1 4.1 4.2 4.1 4.2 4.1
## [5689] 4.2 4.1 4.1 4.1 4.2 4.1 4.2 4.1 4.1 4.2 4.1 4.1 4.1 4.2 4.2 4.1 4.1 4.1
## [5707] 4.1 4.1 4.1 4.2 4.2 4.2 4.2 4.2 4.2 4.2 4.1 4.2 4.2 4.1 4.2 4.2 4.1 4.2
## [5725] 4.1 4.2 4.1 4.1 4.1 4.1 4.1 4.1 4.1 4.1 4.1 4.1 4.1 4.1 4.1 4.1 4.1 4.1
## [5743] 4.2 4.2 4.1 4.1 4.1 4.1 4.1 4.1 4.1 4.1 4.1 4.1 4.2 4.1 4.1 4.1 4.1 4.1
## [5761] 4.1 4.1 4.1 4.1 4.1 4.1 4.2 4.1 4.1 4.1 4.2 4.1 4.1 4.1 4.1 4.1 4.2 4.2
## [5779] 4.1 4.1 4.1 4.1 4.1 4.1 4.1 4.1 4.1 4.1 4.1 4.1 4.1 4.2 4.1 4.1 4.2 4.1
## [5797] 4.1 4.2 4.1 4.2 4.1 4.1 4.2 4.1 4.2 4.1 4.2 4.2 4.1 4.1 4.2 4.2 4.2 4.2
## [5815] 4.1 4.1 4.2 4.2 4.1 4.1 4.1 4.1 4.1 4.1 4.1 4.2 4.1 4.2 4.2 4.2 4.1 4.1
## [5833] 4.1 4.1 4.2 4.1 4.1 4.2 4.1 4.1 4.2 4.1 4.1 4.2 4.1 4.2 4.1 4.1 4.1 4.1
## [5851] 4.1 4.1 4.1 4.1 4.1 4.2 4.1 4.1 4.1 4.2 4.1 4.1 4.1 4.1 4.1 4.1 4.1 4.1
## [5869] 4.1 4.2 4.1 4.1 4.1 4.1 4.2 4.1 4.1 4.1 4.2 4.1 4.1 4.1 4.1 4.1 4.1 4.1
## [5887] 4.2 4.2 4.2 4.1 4.2 4.2 4.1 4.1 4.1 4.1 4.2 4.1 4.1 4.1 4.2 4.1 4.1 4.1
## [5905] 4.2 4.1 4.1 4.1 4.1 4.1 4.1 4.1 4.2 4.1 4.1 4.1 4.1 4.2 4.2 4.1 4.1 4.1
## [5923] 4.1 4.2 4.2 4.1 4.2 4.2 4.1 4.1 4.2 4.2 4.2 4.1 4.1 4.1 4.2 4.1 4.2 4.1
## [5941] 4.1 4.2 4.1 4.2 4.2 4.2 4.2 4.2 4.2 4.2 4.2 4.1 4.2 4.1 4.2 4.1 4.1 4.1
## [5959] 4.2 4.2 4.1 4.1 4.1 4.2 4.1 4.1 4.1 4.2 4.1 4.2 4.1 4.1 4.1 4.1 4.1 4.2
## [5977] 4.1 4.1 4.1 4.2 4.1 4.1 4.1 4.1 4.2 4.1 4.2 4.1 4.1 4.1 4.2 4.1 4.1 4.2
## [5995] 4.1 4.1 4.1 4.1 4.2 4.1 4.1 4.1 4.1 4.1 4.2 4.2 4.2 4.2 4.1 4.2 4.1 4.1
## [6013] 4.1 4.1 4.2 4.1 4.1 4.1 4.1 4.1 4.1 4.1 4.1 4.2 4.2 4.1 4.1 4.1 4.1 4.1
## [6031] 4.1 4.1 4.1 4.2 4.1 4.1 4.1 4.2 4.1 4.1 4.1 4.1 4.2 4.1 4.2 4.1 4.2 4.1
## [6049] 4.1 4.2 4.1 4.1 4.1 4.1 4.1 4.1 4.1 4.2 4.1 4.2 4.1 4.1 4.2 4.2 4.1 4.1
## [6067] 4.2 4.2 4.1 4.1 4.2 4.2 4.1 4.1 4.1 4.2 4.2 4.1 4.2 4.1 4.1 4.1 4.2 4.1
## [6085] 4.2 4.2 4.1 5.2 5.1 5.2 5.1 5.1 5.1 5.1 5.2 5.2 5.1 5.1 5.2 5.1 5.1 5.1
## [6103] 5.1 5.1 5.2 5.1 5.1 5.2 5.2 5.1 5.2 5.2 5.1 5.1 5.2 5.2 5.2 5.2 5.1 5.2
## [6121] 5.1 5.2 5.2 5.1 5.1 5.2 5.2 5.2 5.2 5.2 5.2 5.2 5.1 5.2 5.1 5.1 5.1 5.2
## [6139] 5.1 5.2 5.1 5.1 5.1 5.1 5.1 5.2 5.1 5.1 5.1 5.1 5.2 5.2 5.1 5.1 5.1 5.1
## [6157] 5.2 5.1 5.1 5.1 5.1 5.1 5.2 5.2 5.1 5.2 5.2 5.1 5.1 5.1 5.2 5.2 5.1 5.1
## [6175] 5.1 5.2 5.2 5.2 5.1 5.2 5.2 5.2 5.2 5.2 5.2 5.2 5.1 5.2 5.2 5.2 5.2 5.2
## [6193] 5.1 5.2 5.1 5.1 5.2 5.2 5.2 5.2 5.2 5.2 5.1 5.1 5.1 5.2 5.2 5.1 5.1 5.1
## [6211] 5.2 5.2 5.1 5.1 5.1 5.2 5.1 5.2 5.1 5.1 5.2 5.2 5.1 5.2 5.1 5.1 5.1 5.1
## [6229] 5.2 5.2 5.2 5.1 5.1 5.2 5.2 5.2 5.2 5.2 5.1 5.1 5.2 5.2 5.1 5.2 5.1 5.2
## [6247] 5.2 5.2 5.2 5.1 5.1 5.1 5.1 5.1 5.2 5.1 5.1 5.2 5.2 5.1 5.2 5.1 5.1 5.1
## [6265] 5.2 5.1 5.1 5.2 5.2 5.1 5.2 5.1 5.1 5.2 5.1 5.1 5.1 5.2 5.2 5.2 5.2 5.1
## [6283] 5.1 5.1 5.2 5.2 5.2 5.2 5.2 5.1 5.2 5.1 5.1 5.1 5.1 5.2 5.2 5.2 5.2 5.2
## [6301] 5.2 5.2 5.1 5.2 5.2 5.2 5.2 5.2 5.2 5.1 5.2 5.2 5.1 5.2 5.2 5.2 5.1 5.2
## [6319] 5.2 5.2 5.2 5.1 5.2 5.2 5.2 5.1 5.1 5.1 5.1 5.1 5.1 5.1 5.2 5.1 5.1 5.1
## [6337] 5.1 5.1 5.1 5.1 5.1 5.1 5.1 5.1 5.1 5.1 5.2 5.2 5.2 5.1 5.1 5.1 5.1 5.2
## [6355] 5.1 5.2 5.1 5.1 5.1 5.1 5.1 5.1 5.1 5.1 5.1 5.1 5.1 5.1 5.1 5.1 5.1 5.1
## [6373] 5.1 5.1 5.1 5.1 5.1 5.1 5.1 5.1 5.1 5.1 5.1 5.1 5.1 5.1 5.1 5.1 5.2 5.2
## [6391] 5.1 5.1 5.1 5.1 5.1 5.1 5.1 5.1 5.1 5.1 5.1 5.2 5.2 5.1 5.1 5.1 5.2 5.1
## [6409] 5.1 5.1 5.1 5.1 5.1 5.1 5.1 5.1 5.1 5.1 5.1 5.1 5.1 5.1 5.1 5.2 5.2 5.1
## [6427] 5.1 5.1 5.1 5.1 5.1 5.1 5.1 5.1 5.1 5.1 5.1 5.1 5.1 5.1 5.1 5.1 5.1 5.1
## [6445] 5.1 5.1 5.2 5.1 5.2 5.1 5.1 5.1 5.2 5.1 5.1 5.1 5.1 5.1 5.1 5.2 5.1 5.1
## [6463] 5.1 5.1 5.1 5.2 5.1 5.1 5.1 5.1 5.1 5.1 5.1 5.1 5.1 5.1 5.1 5.1 5.1 5.1
## [6481] 5.2 5.1 5.1 5.1 5.1 5.1 5.1 5.2 5.1 5.1 5.2 5.1 5.1 5.1 5.1 5.1 5.2 5.2
## [6499] 5.1 5.1 5.2 5.1 5.2 5.1 5.1 5.1 5.1 5.1 5.1 5.1 5.1 5.1 5.1 5.2 5.1 5.1
## [6517] 5.1 5.1 5.1 5.1 5.1 5.1 5.1 5.1 5.2 5.2 5.2 5.1 5.1 5.1 5.2 5.1 5.2 5.1
## [6535] 5.1 5.1 5.1 5.1 5.2 5.1 5.1 5.1 5.2 5.1 5.2 5.2 5.1 5.1 5.1 5.1 5.2 5.1
## [6553] 5.2 5.1 5.1 5.1 5.1 5.1 5.1 5.1 5.1 5.1 5.1 5.1 5.1 5.1 5.1 5.2 5.2 5.1
## [6571] 5.1 5.1 5.1 5.2 5.1 5.1 5.1 5.1 5.2 5.1 5.2 5.1 5.1 5.1 5.1 5.1 5.2 5.1
## [6589] 5.1 5.2 5.1 5.1 5.1 5.1 5.1 5.1 5.1 5.1 5.1 5.1 5.1 5.1 5.1 5.1 5.1 5.1
## [6607] 5.1 5.1 5.1 5.2 5.1 5.1 5.1 5.1 5.1 5.2 5.1 5.1 5.1 5.2 5.1 5.2 5.2 5.2
## [6625] 5.1 5.1 5.1 5.1 5.1 5.1 5.1 5.2 5.1 5.1 5.1 5.1 5.1 5.1 5.1 5.1 5.1 5.1
## [6643] 5.1 5.1 5.2 5.1 5.2 5.1 5.1 5.2 5.1 5.1 5.1 5.1 5.1 5.1 5.1 5.1 5.1 5.1
## [6661] 5.1 5.1 5.1 5.1 5.1 5.1 5.1 5.2 5.1 5.1 5.1 5.1 5.1 5.1 5.1 5.1 5.1 5.1
## [6679] 5.1 5.1 5.1 5.1 5.2 5.1 5.1 5.2 5.1 5.1 5.1 5.1 5.1 5.2 5.1 5.1 5.1 5.1
## [6697] 5.1 5.1 5.1 5.2 5.1 5.1 5.1 5.2 5.1 5.1 5.1 5.1 5.1 5.1 5.1 5.2 5.1 5.1
## [6715] 5.1 5.1 5.2 5.1 5.1 5.1 5.1 5.1 5.1 5.1 5.1 5.1 5.1 5.1 5.1 5.1 5.1 5.2
## [6733] 5.1 5.1 5.1 5.1 5.1 5.1 5.2 5.1 5.1 5.1 5.1 5.1 5.1 5.1 5.1 5.1 5.1 5.1
## [6751] 5.1 5.1 5.2 5.1 5.1 5.2 5.2 5.1 5.1 5.1 5.1 5.1 5.2 5.1 5.1 5.1 5.2 5.1
## [6769] 5.1 5.1 5.2 5.1 5.1 5.1 5.1 5.2 5.1 5.1 5.2 5.2 5.1 5.1 5.1 5.1 5.2 5.1
## [6787] 5.1 5.1 5.1 5.2 5.1 5.1 5.2 5.2 5.2 5.2 5.1 5.2 5.1 5.1 5.1 5.1 5.1 5.1
## [6805] 5.1 5.1 5.1 5.1 5.1 5.1 5.1 5.2 5.1 5.2 5.1 5.1 5.1 5.1 5.1 5.1 5.2 5.1
## [6823] 5.1 5.2 5.1 5.1 5.1 5.2 5.1 5.1 5.1 5.1 5.2 5.1 5.1 5.1 5.1 5.2 5.1 5.1
## [6841] 5.1 5.1 5.1 5.1 5.1 5.2 5.1 5.1 5.1 5.1 5.1 5.1 5.1 5.1 5.1 5.1 5.1 5.2
## [6859] 5.1 5.1 5.1 5.2 5.2 5.1 5.1 5.1 5.1 5.2 5.1 5.1 5.1 5.2 5.1 5.1 5.1 5.1
## [6877] 5.1 5.1 5.1 5.1 5.1 5.1 5.1 5.2 5.2 5.1 5.1 5.1 5.1 5.1 5.1 5.1 5.2 5.2
## [6895] 5.2 5.1 5.1 5.2 5.2 5.1 5.2 5.1 5.1 5.2 5.1 5.1 5.1 5.1 5.1 5.1 5.2 5.2
## [6913] 5.1 5.1 5.2 5.1 5.2 5.2 5.2 5.2 5.2 5.1 5.1 5.1 5.1 5.1 5.1 5.2 5.2 5.2
## [6931] 5.2 5.1 5.1 5.1 5.2 5.1 5.2 5.1 5.2 5.1 5.2 5.1 5.2 5.1 5.1 5.1 5.2 5.1
## [6949] 5.1 5.2 5.1 5.1 5.1 5.2 5.2 5.1 5.1 5.1 5.1 5.1 5.2 5.2 5.2 5.2 5.2 5.2
## [6967] 5.2 5.1 5.2 5.2 5.1 5.2 5.1 5.2 5.1 5.2 5.1 5.2 5.1 5.1 5.1 5.1 5.1 5.1
## [6985] 5.1 5.1 5.1 5.1 5.1 5.1 5.1 5.1 5.2 5.2 5.1 5.1 5.1 5.1 5.1 5.1 5.1 5.1
## [7003] 5.2 5.1 5.1 5.1 5.1 5.1 5.1 5.1 5.1 5.1 5.1 5.1 5.2 5.1 5.2 5.1 5.1 5.1
## [7021] 5.2 5.2 5.1 5.1 5.1 5.1 5.1 5.1 5.1 5.1 5.1 5.2 5.1 5.1 5.2 5.1 5.2 5.1
## [7039] 5.2 5.1 5.1 5.1 5.1 5.1 5.1 5.1 5.2 5.2 5.1 5.1 5.1 5.1 5.2 5.1 5.1 5.2
## [7057] 5.1 5.2 5.1 5.1 5.1 5.1 5.1 5.1 5.1 5.1 5.2 5.1 5.1 5.1 5.2 5.1 5.1 5.1
## [7075] 5.1 5.1 5.1 5.1 5.2 5.1 5.1 5.1 5.1 5.2 5.1 5.2 5.1 5.2 5.1 5.1 5.1 5.1
## [7093] 5.1 5.1 5.1 5.1 5.2 5.2 5.2 5.2 5.1 5.2 5.1 5.1 5.1 5.1 5.2 5.1 5.1 5.1
## [7111] 5.1 5.1 5.1 5.1 5.1 5.2 5.2 5.1 5.1 5.2 5.1 5.1 5.1 5.1 5.1 5.1 5.1 5.2
## [7129] 5.1 5.2 5.1 5.1 5.1 5.2 5.1 5.1 5.1 5.1 5.2 5.1 5.2 5.1 5.2 5.1 5.1 5.1
## [7147] 5.2 5.1 5.1 5.1 5.1 5.1 5.1 5.1 5.1 5.1 5.2 5.1 5.1 5.1 5.2 5.1 5.1 5.2
## [7165] 5.2 5.2 5.2 5.1 5.2 5.1 5.1 5.1 5.2 5.2 5.2 5.1 5.2 5.1 5.1 5.2 5.2 5.2
## [7183] 5.1 5.2 5.2 5.2 5.2 5.1 6.1 6.1 6.2 6.2 6.2 6.2 6.2 6.1 6.2 6.2 6.2 6.1
## [7201] 6.1 6.2 6.2 6.2 6.2 6.2 6.2 6.2 6.1 6.2 6.1 6.1 6.1 6.2 6.1 6.2 6.1 6.1
## [7219] 6.1 6.1 6.1 6.1 6.2 6.1 6.1 6.1 6.2 6.2 6.1 6.1 6.1 6.1 6.1 6.2 6.1 6.1
## [7237] 6.1 6.1 6.1 6.2 6.2 6.1 6.2 6.2 6.1 6.1 6.1 6.2 6.2 6.2 6.2 6.2 6.2 6.1
## [7255] 6.1 6.1 6.2 6.2 6.2 6.1 6.2 6.2 6.2 6.2 6.2 6.2 6.2 6.2 6.2 6.2 6.2 6.1
## [7273] 6.2 6.2 6.2 6.1 6.2 6.2 6.2 6.2 6.2 6.2 6.1 6.1 6.2 6.1 6.1 6.1 6.2 6.2
## [7291] 6.1 6.1 6.2 6.1 6.2 6.1 6.2 6.2 6.1 6.2 6.1 6.1 6.1 6.1 6.2 6.2 6.2 6.1
## [7309] 6.2 6.2 6.2 6.2 6.2 6.1 6.1 6.2 6.2 6.1 6.2 6.1 6.2 6.2 6.2 6.2 6.1 6.1
## [7327] 6.1 6.1 6.1 6.1 6.2 6.1 6.2 6.1 6.2 6.1 6.1 6.1 6.1 6.2 6.1 6.2 6.2 6.1
## [7345] 6.2 6.1 6.1 6.2 6.1 6.1 6.1 6.2 6.2 6.1 6.1 6.2 6.2 6.2 6.2 6.2 6.1 6.2
## [7363] 6.1 6.1 6.1 6.1 6.2 6.2 6.2 6.2 6.2 6.2 6.2 6.1 6.2 6.2 6.2 6.2 6.2 6.2
## [7381] 6.1 6.2 6.2 6.1 6.2 6.2 6.2 6.1 6.2 6.2 6.2 6.1 6.2 6.2 6.2 6.1 6.1 6.1
## [7399] 6.1 6.1 6.1 6.1 6.2 6.1 6.2 6.1 6.1 6.1 6.1 6.1 6.1 6.2 6.2 6.2 6.1 6.1
## [7417] 6.1 6.1 6.1 6.1 6.1 6.1 6.1 6.2 6.1 6.1 6.1 6.1 6.1 6.1 6.1 6.1 6.1 6.1
## [7435] 6.1 6.1 6.2 6.2 6.2 6.1 6.1 6.1 6.1 6.2 6.1 6.2 6.1 6.1 6.1 6.1 6.1 6.1
## [7453] 6.1 6.1 6.1 6.1 6.1 6.1 6.1 6.1 6.1 6.1 6.1 6.1 6.1 6.1 6.1 6.1 6.1 6.1
## [7471] 6.1 6.1 6.1 6.1 6.1 6.1 6.2 6.2 6.1 6.1 6.1 6.1 6.1 6.1 6.1 6.1 6.1 6.2
## [7489] 6.2 6.1 6.2 6.1 6.1 6.1 6.1 6.1 6.2 6.1 6.1 6.1 6.1 6.1 6.1 6.1 6.1 6.1
## [7507] 6.1 6.2 6.2 6.1 6.1 6.1 6.1 6.1 6.1 6.1 6.1 6.1 6.1 6.1 6.1 6.1 6.1 6.1
## [7525] 6.1 6.1 6.1 6.1 6.2 6.1 6.2 6.1 6.1 6.1 6.2 6.1 6.1 6.1 6.1 6.1 6.1 6.2
## [7543] 6.1 6.1 6.1 6.1 6.2 6.1 6.1 6.1 6.1 6.1 6.1 6.1 6.1 6.1 6.1 6.1 6.1 6.2
## [7561] 6.1 6.1 6.1 6.1 6.1 6.1 6.2 6.1 6.1 6.2 6.1 6.1 6.1 6.1 6.1 6.2 6.2 6.1
## [7579] 6.1 6.2 6.1 6.1 6.1 6.1 6.1 6.1 6.1 6.1 6.1 6.2 6.1 6.1 6.1 6.1 6.1 6.1
## [7597] 6.1 6.1 6.1 6.1 6.2 6.2 6.1 6.1 6.1 6.1 6.2 6.1 6.1 6.1 6.1 6.1 6.2 6.1
## [7615] 6.1 6.2 6.1 6.2 6.2 6.1 6.1 6.1 6.2 6.1 6.1 6.1 6.1 6.1 6.1 6.1 6.1 6.1
## [7633] 6.1 6.1 6.1 6.1 6.1 6.2 6.2 6.1 6.1 6.1 6.1 6.2 6.1 6.1 6.1 6.1 6.1 6.2
## [7651] 6.1 6.1 6.1 6.1 6.1 6.2 6.1 6.1 6.1 6.1 6.1 6.1 6.1 6.1 6.1 6.1 6.1 6.1
## [7669] 6.1 6.1 6.1 6.1 6.1 6.1 6.1 6.1 6.1 6.1 6.2 6.1 6.1 6.1 6.1 6.1 6.2 6.1
## [7687] 6.1 6.1 6.2 6.1 6.2 6.2 6.2 6.1 6.1 6.1 6.1 6.1 6.1 6.2 6.1 6.1 6.1 6.1
## [7705] 6.1 6.1 6.1 6.1 6.1 6.2 6.1 6.2 6.1 6.1 6.2 6.1 6.1 6.1 6.1 6.1 6.1 6.1
## [7723] 6.1 6.1 6.1 6.1 6.1 6.1 6.1 6.1 6.1 6.2 6.1 6.1 6.1 6.1 6.1 6.1 6.1 6.1
## [7741] 6.1 6.1 6.1 6.1 6.1 6.2 6.1 6.1 6.2 6.1 6.1 6.1 6.1 6.1 6.2 6.1 6.1 6.1
## [7759] 6.1 6.1 6.1 6.1 6.1 6.2 6.1 6.1 6.1 6.1 6.1 6.1 6.1 6.1 6.1 6.1 6.2 6.1
## [7777] 6.1 6.1 6.1 6.1 6.1 6.1 6.1 6.1 6.1 6.1 6.1 6.1 6.1 6.1 6.1 6.1 6.1 6.1
## [7795] 6.2 6.1 6.1 6.1 6.1 6.2 6.1 6.1 6.1 6.1 6.1 6.1 6.1 6.1 6.1 6.1 6.1 6.2
## [7813] 6.1 6.1 6.2 6.2 6.1 6.1 6.1 6.1 6.2 6.1 6.1 6.1 6.1 6.1 6.2 6.1 6.1 6.1
## [7831] 6.2 6.1 6.1 6.2 6.2 6.1 6.1 6.1 6.1 6.2 6.1 6.1 6.1 6.1 6.2 6.1 6.2 6.2
## [7849] 6.2 6.2 6.1 6.2 6.1 6.1 6.1 6.1 6.1 6.1 6.1 6.1 6.1 6.1 6.1 6.1 6.2 6.1
## [7867] 6.2 6.1 6.1 6.1 6.1 6.1 6.2 6.1 6.1 6.2 6.1 6.1 6.1 6.2 6.1 6.1 6.1 6.1
## [7885] 6.2 6.1 6.1 6.1 6.1 6.2 6.1 6.1 6.1 6.1 6.1 6.1 6.2 6.1 6.1 6.1 6.1 6.1
## [7903] 6.1 6.1 6.1 6.1 6.1 6.1 6.2 6.1 6.1 6.1 6.2 6.2 6.1 6.1 6.1 6.2 6.1 6.1
## [7921] 6.1 6.2 6.1 6.1 6.1 6.1 6.1 6.1 6.1 6.1 6.1 6.1 6.1 6.2 6.2 6.1 6.1 6.1
## [7939] 6.1 6.1 6.1 6.1 6.2 6.2 6.2 6.1 6.1 6.2 6.2 6.1 6.2 6.1 6.1 6.2 6.1 6.1
## [7957] 6.1 6.1 6.1 6.1 6.2 6.2 6.1 6.1 6.2 6.1 6.2 6.2 6.1 6.2 6.2 6.1 6.1 6.1
## [7975] 6.1 6.1 6.2 6.2 6.2 6.2 6.1 6.1 6.1 6.2 6.1 6.2 6.1 6.2 6.1 6.2 6.1 6.1
## [7993] 6.1 6.1 6.2 6.1 6.1 6.1 6.2 6.1 6.1 6.1 6.2 6.1 6.1 6.1 6.1 6.2 6.2 6.2
## [8011] 6.2 6.2 6.2 6.2 6.1 6.2 6.1 6.2 6.1 6.2 6.1 6.2 6.1 6.2 6.1 6.1 6.1 6.1
## [8029] 6.1 6.1 6.1 6.1 6.1 6.1 6.1 6.1 6.1 6.1 6.1 6.1 6.2 6.2 6.1 6.1 6.1 6.1
## [8047] 6.1 6.1 6.1 6.1 6.1 6.2 6.1 6.1 6.1 6.1 6.1 6.1 6.1 6.1 6.1 6.1 6.1 6.1
## [8065] 6.2 6.1 6.2 6.1 6.1 6.1 6.2 6.2 6.1 6.1 6.1 6.1 6.1 6.1 6.1 6.1 6.1 6.2
## [8083] 6.1 6.2 6.1 6.2 6.1 6.2 6.1 6.1 6.2
## Levels: 1.1 2.1 3.1 4.1 5.1 6.1 1.2 2.2 3.2 4.2 5.2 6.2
```

```
M.SWLS = lme(SWLS ~ MZP*CLASS, data = GM6, random = ~1|ID/MZP)
summary(M.SWLS)
```

```
## Linear mixed-effects model fit by REML
##  Data: GM6 
##     AIC   BIC  logLik
##   18811 18916 -9390.3
## 
## Random effects:
##  Formula: ~1 | ID
##         (Intercept)
## StdDev:     0.51672
## 
##  Formula: ~1 | MZP %in% ID
##         (Intercept) Residual
## StdDev:     0.61542  0.25534
## 
## Fixed effects: SWLS ~ MZP * CLASS 
##               Value Std.Error   DF t-value p-value
## (Intercept)  5.6603  0.023705 6179 238.784  0.0000
## MZP2         0.0268  0.027138 6179   0.986  0.3242
## MZP3         0.1022  0.029670 6179   3.446  0.0006
## MZP4         0.1168  0.029553 6179   3.952  0.0001
## MZP5         0.0444  0.031344 6179   1.416  0.1568
## MZP6         0.0853  0.033536 6179   2.545  0.0110
## CLASS2      -1.8455  0.043340 1900 -42.582  0.0000
## MZP2:CLASS2 -0.2398  0.050240 6179  -4.772  0.0000
## MZP3:CLASS2 -0.2962  0.054268 6179  -5.457  0.0000
## MZP4:CLASS2 -0.2908  0.054779 6179  -5.308  0.0000
## MZP5:CLASS2 -0.1821  0.058030 6179  -3.137  0.0017
## MZP6:CLASS2 -0.2863  0.062335 6179  -4.593  0.0000
##  Correlation: 
##             (Intr) MZP2   MZP3   MZP4   MZP5   MZP6   CLASS2 MZP2:C MZP3:C
## MZP2        -0.561                                                        
## MZP3        -0.514  0.448                                                 
## MZP4        -0.518  0.450  0.433                                          
## MZP5        -0.487  0.426  0.408  0.421                                   
## MZP6        -0.456  0.400  0.383  0.395  0.389                            
## CLASS2      -0.547  0.307  0.281  0.283  0.267  0.249                     
## MZP2:CLASS2  0.303 -0.540 -0.242 -0.243 -0.230 -0.216 -0.554              
## MZP3:CLASS2  0.281 -0.245 -0.547 -0.237 -0.223 -0.210 -0.515  0.441       
## MZP4:CLASS2  0.279 -0.243 -0.234 -0.539 -0.227 -0.213 -0.511  0.439  0.428
## MZP5:CLASS2  0.263 -0.230 -0.220 -0.228 -0.540 -0.210 -0.483  0.417  0.404
## MZP6:CLASS2  0.245 -0.215 -0.206 -0.213 -0.209 -0.538 -0.450  0.389  0.379
##             MZP4:C MZP5:C
## MZP2                     
## MZP3                     
## MZP4                     
## MZP5                     
## MZP6                     
## CLASS2                   
## MZP2:CLASS2              
## MZP3:CLASS2              
## MZP4:CLASS2              
## MZP5:CLASS2  0.412       
## MZP6:CLASS2  0.387  0.382
## 
## Standardized Within-Group Residuals:
##       Min        Q1       Med        Q3       Max 
## -2.827399 -0.163372  0.030039  0.188608  1.505953 
## 
## Number of Observations: 8091
## Number of Groups: 
##          ID MZP %in% ID 
##        1902        8091
```

```
anova(M.SWLS)
```

```
##             numDF denDF F-value p-value
## (Intercept)     1  6179  129216  <.0001
## MZP             5  6179       2  0.0873
## CLASS           1  1900    4306  <.0001
## MZP:CLASS       5  6179       9  <.0001
```

```
MI.SWLS = lme(SWLS ~ INT, data = GM6, random = ~1|ID/MZP)
MCP.SWLS = glht(MI.SWLS, linfct = mcp(INT = CH2))
summary(MCP.SWLS)
```

```
## 
##   Simultaneous Tests for General Linear Hypotheses
## 
## Multiple Comparisons of Means: User-defined Contrasts
## 
## 
## Fit: lme.formula(fixed = SWLS ~ INT, data = GM6, random = ~1 | ID/MZP)
## 
## Linear Hypotheses:
##             Estimate Std. Error z value Pr(>|z|)    
## G1|G2 == 0   -2.0613     0.0316  -65.20   <0.001 ***
## G1: M1 == 0   0.0626     0.0184    3.41   0.0085 ** 
## G1: M2 == 0   0.0358     0.0186    1.93   0.4914    
## G1: M3 == 0  -0.0396     0.0206   -1.93   0.4923    
## G1: M4 == 0  -0.0542     0.0203   -2.67   0.0920 .  
## G1: M5 == 0   0.0182     0.0219    0.83   0.9974    
## G1: M6 == 0  -0.0228     0.0239   -0.95   0.9918    
## G2: M1 == 0  -0.1533     0.0284   -5.40   <0.001 ***
## G2: M2 == 0   0.0597     0.0294    2.03   0.4091    
## G2: M3 == 0   0.0407     0.0317    1.28   0.9258    
## G2: M4 == 0   0.0207     0.0320    0.65   0.9997    
## G2: M5 == 0  -0.0156     0.0343   -0.45   1.0000    
## G2: M6 == 0   0.0477     0.0377    1.26   0.9330    
## ---
## Signif. codes:  0 '***' 0.001 '**' 0.01 '*' 0.05 '.' 0.1 ' ' 1
## (Adjusted p values reported -- single-step method)
```

```
# C3
GM6 = data.frame(rep(c(1:nrow(M6)), 6), rep(M6$SWLS_C3, 6), rep(c(1:6), each = nrow(M6)), c(M6$SWLS_1, M6$SWLS_2, M6$SWLS_3, M6$SWLS_4, M6$SWLS_5, M6$SWLS_6))
colnames(GM6) = c("ID", "CLASS", "MZP", "SWLS")
GM6$ID = factor(GM6$ID)
GM6$CLASS = factor(GM6$CLASS)
GM6$MZP = factor(GM6$MZP)
GM6 = na.omit(GM6)
GM6$INT = interaction(GM6$MZP, GM6$CLASS)

M.SWLS = lme(SWLS ~ MZP*CLASS, data = GM6, random = ~1|ID/MZP)
summary(M.SWLS)
```

```
## Linear mixed-effects model fit by REML
##  Data: GM6 
##     AIC   BIC  logLik
##   17533 17680 -8745.5
## 
## Random effects:
##  Formula: ~1 | ID
##         (Intercept)
## StdDev:     0.29681
## 
##  Formula: ~1 | MZP %in% ID
##         (Intercept) Residual
## StdDev:     0.61953  0.23185
## 
## Fixed effects: SWLS ~ MZP * CLASS 
##               Value Std.Error   DF t-value p-value
## (Intercept)  5.9561  0.024430 6174 243.808  0.0000
## MZP2         0.0229  0.031962 6174   0.718  0.4728
## MZP3         0.0277  0.034817 6174   0.797  0.4255
## MZP4         0.0618  0.034540 6174   1.790  0.0735
## MZP5         0.0289  0.036422 6174   0.793  0.4278
## MZP6         0.0738  0.038901 6174   1.896  0.0580
## CLASS2      -1.1763  0.038562 1899 -30.504  0.0000
## CLASS3      -2.6012  0.047738 1899 -54.489  0.0000
## MZP2:CLASS2 -0.0780  0.050717 6174  -1.538  0.1241
## MZP3:CLASS2  0.1198  0.054784 6174   2.186  0.0288
## MZP4:CLASS2  0.0837  0.054610 6174   1.533  0.1254
## MZP5:CLASS2 -0.0737  0.057763 6174  -1.276  0.2020
## MZP6:CLASS2 -0.0752  0.061912 6174  -1.215  0.2244
## MZP2:CLASS3 -0.2354  0.063238 6174  -3.723  0.0002
## MZP3:CLASS3 -0.3079  0.067954 6174  -4.531  0.0000
## MZP4:CLASS3 -0.3654  0.069197 6174  -5.280  0.0000
## MZP5:CLASS3 -0.1119  0.073437 6174  -1.524  0.1275
## MZP6:CLASS3 -0.2824  0.078364 6174  -3.604  0.0003
##  Correlation: 
##             (Intr) MZP2   MZP3   MZP4   MZP5   MZP6   CLASS2 CLASS3 MZP2:CLASS2
## MZP2        -0.643                                                             
## MZP3        -0.591  0.452                                                      
## MZP4        -0.597  0.456  0.430                                               
## MZP5        -0.566  0.433  0.408  0.417                                        
## MZP6        -0.530  0.406  0.383  0.391  0.380                                 
## CLASS2      -0.634  0.408  0.375  0.378  0.358  0.336                          
## CLASS3      -0.512  0.329  0.303  0.306  0.289  0.271  0.324                   
## MZP2:CLASS2  0.406 -0.630 -0.285 -0.287 -0.273 -0.256 -0.640 -0.208            
## MZP3:CLASS2  0.376 -0.287 -0.636 -0.273 -0.259 -0.243 -0.593 -0.192  0.451     
## MZP4:CLASS2  0.378 -0.288 -0.272 -0.632 -0.264 -0.248 -0.596 -0.193  0.452     
## MZP5:CLASS2  0.357 -0.273 -0.257 -0.263 -0.631 -0.240 -0.563 -0.182  0.429     
## MZP6:CLASS2  0.333 -0.255 -0.240 -0.246 -0.239 -0.628 -0.526 -0.170  0.401     
## MZP2:CLASS3  0.325 -0.505 -0.228 -0.230 -0.219 -0.205 -0.206 -0.636  0.319     
## MZP3:CLASS3  0.303 -0.231 -0.512 -0.220 -0.209 -0.196 -0.192 -0.593  0.146     
## MZP4:CLASS3  0.298 -0.228 -0.215 -0.499 -0.208 -0.195 -0.189 -0.584  0.143     
## MZP5:CLASS3  0.280 -0.215 -0.202 -0.207 -0.496 -0.189 -0.178 -0.550  0.135     
## MZP6:CLASS3  0.263 -0.202 -0.190 -0.194 -0.189 -0.496 -0.167 -0.515  0.127     
##             MZP3:CLASS2 MZP4:CLASS2 MZP5:CLASS2 MZP6:CLASS2 MZP2:CLASS3
## MZP2                                                                   
## MZP3                                                                   
## MZP4                                                                   
## MZP5                                                                   
## MZP6                                                                   
## CLASS2                                                                 
## CLASS3                                                                 
## MZP2:CLASS2                                                            
## MZP3:CLASS2                                                            
## MZP4:CLASS2  0.429                                                     
## MZP5:CLASS2  0.405       0.413                                         
## MZP6:CLASS2  0.380       0.387       0.376                             
## MZP2:CLASS3  0.145       0.146       0.138       0.129                 
## MZP3:CLASS3  0.326       0.139       0.132       0.123       0.446     
## MZP4:CLASS3  0.136       0.316       0.131       0.123       0.439     
## MZP5:CLASS3  0.129       0.131       0.313       0.119       0.415     
## MZP6:CLASS3  0.121       0.123       0.119       0.312       0.389     
##             MZP3:CLASS3 MZP4:CLASS3 MZP5:CLASS3
## MZP2                                           
## MZP3                                           
## MZP4                                           
## MZP5                                           
## MZP6                                           
## CLASS2                                         
## CLASS3                                         
## MZP2:CLASS2                                    
## MZP3:CLASS2                                    
## MZP4:CLASS2                                    
## MZP5:CLASS2                                    
## MZP6:CLASS2                                    
## MZP2:CLASS3                                    
## MZP3:CLASS3                                    
## MZP4:CLASS3  0.422                             
## MZP5:CLASS3  0.398       0.399                 
## MZP6:CLASS3  0.374       0.375       0.364     
## 
## Standardized Within-Group Residuals:
##       Min        Q1       Med        Q3       Max 
## -2.632074 -0.163203  0.011576  0.190205  1.402848 
## 
## Number of Observations: 8091
## Number of Groups: 
##          ID MZP %in% ID 
##        1902        8091
```

```
anova(M.SWLS)
```

```
##             numDF denDF F-value p-value
## (Intercept)     1  6174  251152  <.0001
## MZP             5  6174       2  0.0489
## CLASS           2  1899    5021  <.0001
## MZP:CLASS      10  6174       8  <.0001
```

```
MI.SWLS = lme(SWLS ~ INT, data = GM6, random = ~1|ID/MZP)
MCP.SWLS = glht(MI.SWLS, linfct = mcp(INT = CH3))
summary(MCP.SWLS)
```

```
## 
##   Simultaneous Tests for General Linear Hypotheses
## 
## Multiple Comparisons of Means: User-defined Contrasts
## 
## 
## Fit: lme.formula(fixed = SWLS ~ INT, data = GM6, random = ~1 | ID/MZP)
## 
## Linear Hypotheses:
##             Estimate Std. Error z value Pr(>|z|)    
## G1|G2 == 0  -1.18021    0.02339  -50.46   <0.001 ***
## G1|G3 == 0  -2.81835    0.02938  -95.92   <0.001 ***
## G2|G3 == 0  -1.63814    0.03119  -52.52   <0.001 ***
## G1: M1 == 0  0.03586    0.02146    1.67   0.8468    
## G1: M2 == 0  0.01291    0.02168    0.60   1.0000    
## G1: M3 == 0  0.00811    0.02416    0.34   1.0000    
## G1: M4 == 0 -0.02596    0.02378   -1.09   0.9970    
## G1: M5 == 0  0.00698    0.02549    0.27   1.0000    
## G1: M6 == 0 -0.03789    0.02781   -1.36   0.9698    
## G2: M1 == 0  0.03194    0.02625    1.22   0.9901    
## G2: M2 == 0  0.08700    0.02686    3.24   0.0245 *  
## G2: M3 == 0 -0.11557    0.02940   -3.93   0.0017 ** 
## G2: M4 == 0 -0.11357    0.02928   -3.88   0.0022 ** 
## G2: M5 == 0  0.07677    0.03154    2.43   0.2586    
## G2: M6 == 0  0.03342    0.03461    0.97   0.9993    
## G3: M1 == 0 -0.18132    0.03651   -4.97   <0.001 ***
## G3: M2 == 0  0.03114    0.03775    0.82   0.9999    
## G3: M3 == 0  0.09884    0.04076    2.42   0.2643    
## G3: M4 == 0  0.12225    0.04200    2.91   0.0710 .  
## G3: M5 == 0 -0.09827    0.04540   -2.16   0.4534    
## G3: M6 == 0  0.02735    0.04931    0.55   1.0000    
## ---
## Signif. codes:  0 '***' 0.001 '**' 0.01 '*' 0.05 '.' 0.1 ' ' 1
## (Adjusted p values reported -- single-step method)
```

```
# C4
GM6 = data.frame(rep(c(1:nrow(M6)), 6), rep(M6$SWLS_C4, 6), rep(c(1:6), each = nrow(M6)), c(M6$SWLS_1, M6$SWLS_2, M6$SWLS_3, M6$SWLS_4, M6$SWLS_5, M6$SWLS_6))
colnames(GM6) = c("ID", "CLASS", "MZP", "SWLS")
GM6$ID = factor(GM6$ID)
GM6$CLASS = factor(GM6$CLASS)
GM6$MZP = factor(GM6$MZP)
GM6 = na.omit(GM6)
GM6$INT = interaction(GM6$MZP, GM6$CLASS)

M.SWLS = lme(SWLS ~ MZP*CLASS, data = GM6, random = ~1|ID/MZP)
summary(M.SWLS)
```

```
## Linear mixed-effects model fit by REML
##  Data: GM6 
##     AIC   BIC  logLik
##   16706 16895 -8326.1
## 
## Random effects:
##  Formula: ~1 | ID
##         (Intercept)
## StdDev:     0.15476
## 
##  Formula: ~1 | MZP %in% ID
##         (Intercept) Residual
## StdDev:     0.62378  0.20692
## 
## Fixed effects: SWLS ~ MZP * CLASS 
##               Value Std.Error   DF t-value p-value
## (Intercept)  6.0236  0.024326 6169 247.625  0.0000
## MZP2         0.0140  0.033757 6169   0.413  0.6794
## MZP3         0.0325  0.036678 6169   0.886  0.3756
## MZP4         0.0717  0.036348 6169   1.973  0.0485
## MZP5         0.0299  0.038152 6169   0.783  0.4339
## MZP6         0.0769  0.040641 6169   1.893  0.0585
## CLASS2      -1.0113  0.037887 1898 -26.693  0.0000
## CLASS3      -1.8594  0.045096 1898 -41.233  0.0000
## CLASS4      -3.2320  0.060239 1898 -53.653  0.0000
## MZP2:CLASS2  0.0189  0.052886 6169   0.358  0.7206
## MZP3:CLASS2  0.1825  0.056804 6169   3.213  0.0013
## MZP4:CLASS2  0.1154  0.056696 6169   2.036  0.0418
## MZP5:CLASS2  0.0187  0.059950 6169   0.313  0.7547
## MZP6:CLASS2  0.0047  0.063801 6169   0.074  0.9412
## MZP2:CLASS3 -0.2263  0.063142 6169  -3.585  0.0003
## MZP3:CLASS3 -0.2988  0.067797 6169  -4.407  0.0000
## MZP4:CLASS3 -0.2659  0.067634 6169  -3.932  0.0001
## MZP5:CLASS3 -0.2525  0.071149 6169  -3.549  0.0004
## MZP6:CLASS3 -0.3370  0.076439 6169  -4.409  0.0000
## MZP2:CLASS4 -0.2678  0.084727 6169  -3.161  0.0016
## MZP3:CLASS4 -0.2827  0.090722 6169  -3.116  0.0018
## MZP4:CLASS4 -0.3522  0.093349 6169  -3.773  0.0002
## MZP5:CLASS4 -0.0366  0.099094 6169  -0.369  0.7121
## MZP6:CLASS4 -0.2163  0.106191 6169  -2.037  0.0417
##  Correlation: 
##             (Intr) MZP2   MZP3   MZP4   MZP5   MZP6   CLASS2 CLASS3 CLASS4
## MZP2        -0.685                                                        
## MZP3        -0.630  0.454                                                 
## MZP4        -0.637  0.459  0.426                                          
## MZP5        -0.606  0.437  0.406  0.412                                   
## MZP6        -0.569  0.410  0.381  0.387  0.372                            
## CLASS2      -0.642  0.440  0.405  0.409  0.389  0.365                     
## CLASS3      -0.539  0.369  0.340  0.343  0.327  0.307  0.346              
## CLASS4      -0.404  0.277  0.255  0.257  0.245  0.230  0.259  0.218       
## MZP2:CLASS2  0.437 -0.638 -0.290 -0.293 -0.279 -0.262 -0.681 -0.236 -0.177
## MZP3:CLASS2  0.407 -0.293 -0.646 -0.275 -0.262 -0.246 -0.634 -0.220 -0.164
## MZP4:CLASS2  0.408 -0.294 -0.273 -0.641 -0.264 -0.248 -0.636 -0.220 -0.165
## MZP5:CLASS2  0.386 -0.278 -0.258 -0.262 -0.636 -0.237 -0.601 -0.208 -0.156
## MZP6:CLASS2  0.363 -0.261 -0.243 -0.247 -0.237 -0.637 -0.565 -0.196 -0.146
## MZP2:CLASS3  0.366 -0.535 -0.243 -0.245 -0.234 -0.219 -0.235 -0.679 -0.148
## MZP3:CLASS3  0.341 -0.246 -0.541 -0.231 -0.219 -0.206 -0.219 -0.632 -0.138
## MZP4:CLASS3  0.342 -0.246 -0.229 -0.537 -0.221 -0.208 -0.220 -0.634 -0.138
## MZP5:CLASS3  0.325 -0.234 -0.218 -0.221 -0.536 -0.200 -0.209 -0.603 -0.131
## MZP6:CLASS3  0.303 -0.218 -0.203 -0.206 -0.198 -0.532 -0.194 -0.561 -0.122
## MZP2:CLASS4  0.273 -0.398 -0.181 -0.183 -0.174 -0.164 -0.175 -0.147 -0.676
## MZP3:CLASS4  0.255 -0.184 -0.404 -0.172 -0.164 -0.154 -0.164 -0.137 -0.632
## MZP4:CLASS4  0.248 -0.179 -0.166 -0.389 -0.160 -0.151 -0.159 -0.134 -0.615
## MZP5:CLASS4  0.233 -0.168 -0.156 -0.159 -0.385 -0.143 -0.150 -0.126 -0.580
## MZP6:CLASS4  0.218 -0.157 -0.146 -0.148 -0.142 -0.383 -0.140 -0.118 -0.540
##             MZP2:CLASS2 MZP3:CLASS2 MZP4:CLASS2 MZP5:CLASS2 MZP6:CLASS2
## MZP2                                                                   
## MZP3                                                                   
## MZP4                                                                   
## MZP5                                                                   
## MZP6                                                                   
## CLASS2                                                                 
## CLASS3                                                                 
## CLASS4                                                                 
## MZP2:CLASS2                                                            
## MZP3:CLASS2  0.454                                                     
## MZP4:CLASS2  0.455       0.428                                         
## MZP5:CLASS2  0.431       0.404       0.408                             
## MZP6:CLASS2  0.405       0.380       0.383       0.367                 
## MZP2:CLASS3  0.341       0.157       0.157       0.149       0.140     
## MZP3:CLASS3  0.157       0.349       0.148       0.140       0.131     
## MZP4:CLASS3  0.157       0.148       0.345       0.141       0.133     
## MZP5:CLASS3  0.150       0.140       0.142       0.341       0.127     
## MZP6:CLASS3  0.139       0.131       0.132       0.126       0.339     
## MZP2:CLASS4  0.254       0.117       0.117       0.111       0.104     
## MZP3:CLASS4  0.117       0.261       0.110       0.104       0.098     
## MZP4:CLASS4  0.114       0.107       0.250       0.102       0.096     
## MZP5:CLASS4  0.107       0.101       0.102       0.245       0.091     
## MZP6:CLASS4  0.100       0.094       0.095       0.091       0.244     
##             MZP2:CLASS3 MZP3:CLASS3 MZP4:CLASS3 MZP5:CLASS3 MZP6:CLASS3
## MZP2                                                                   
## MZP3                                                                   
## MZP4                                                                   
## MZP5                                                                   
## MZP6                                                                   
## CLASS2                                                                 
## CLASS3                                                                 
## CLASS4                                                                 
## MZP2:CLASS2                                                            
## MZP3:CLASS2                                                            
## MZP4:CLASS2                                                            
## MZP5:CLASS2                                                            
## MZP6:CLASS2                                                            
## MZP2:CLASS3                                                            
## MZP3:CLASS3  0.451                                                     
## MZP4:CLASS3  0.453       0.425                                         
## MZP5:CLASS3  0.430       0.404       0.408                             
## MZP6:CLASS3  0.401       0.377       0.380       0.365                 
## MZP2:CLASS4  0.213       0.098       0.098       0.093       0.087     
## MZP3:CLASS4  0.098       0.219       0.093       0.088       0.082     
## MZP4:CLASS4  0.095       0.090       0.209       0.086       0.080     
## MZP5:CLASS4  0.090       0.084       0.085       0.206       0.076     
## MZP6:CLASS4  0.084       0.079       0.080       0.076       0.203     
##             MZP2:CLASS4 MZP3:CLASS4 MZP4:CLASS4 MZP5:CLASS4
## MZP2                                                       
## MZP3                                                       
## MZP4                                                       
## MZP5                                                       
## MZP6                                                       
## CLASS2                                                     
## CLASS3                                                     
## CLASS4                                                     
## MZP2:CLASS2                                                
## MZP3:CLASS2                                                
## MZP4:CLASS2                                                
## MZP5:CLASS2                                                
## MZP6:CLASS2                                                
## MZP2:CLASS3                                                
## MZP3:CLASS3                                                
## MZP4:CLASS3                                                
## MZP5:CLASS3                                                
## MZP6:CLASS3                                                
## MZP2:CLASS4                                                
## MZP3:CLASS4  0.448                                         
## MZP4:CLASS4  0.437       0.413                             
## MZP5:CLASS4  0.412       0.390       0.382                 
## MZP6:CLASS4  0.384       0.364       0.357       0.340     
## 
## Standardized Within-Group Residuals:
##        Min         Q1        Med         Q3        Max 
## -2.4218288 -0.1631429 -0.0023584  0.1853930  1.3772431 
## 
## Number of Observations: 8091
## Number of Groups: 
##          ID MZP %in% ID 
##        1902        8091
```

```
anova(M.SWLS)
```

```
##             numDF denDF F-value p-value
## (Intercept)     1  6169  389424  <.0001
## MZP             5  6169       3  0.0158
## CLASS           3  1898    5474  <.0001
## MZP:CLASS      15  6169       6  <.0001
```

```
# C5
GM6 = data.frame(rep(c(1:nrow(M6)), 6), rep(M6$SWLS_C5, 6), rep(c(1:6), each = nrow(M6)), c(M6$SWLS_1, M6$SWLS_2, M6$SWLS_3, M6$SWLS_4, M6$SWLS_5, M6$SWLS_6))
colnames(GM6) = c("ID", "CLASS", "MZP", "SWLS")
GM6$ID = factor(GM6$ID)
GM6$CLASS = factor(GM6$CLASS)
GM6$MZP = factor(GM6$MZP)
GM6 = na.omit(GM6)
GM6$INT = interaction(GM6$MZP, GM6$CLASS)

M.SWLS = lme(SWLS ~ MZP*CLASS, data = GM6, random = ~1|ID/MZP)
summary(M.SWLS)
```

```
## Linear mixed-effects model fit by REML
##  Data: GM6 
##     AIC   BIC logLik
##   16082 16313  -8008
## 
## Random effects:
##  Formula: ~1 | ID
##         (Intercept)
## StdDev:  8.4843e-05
## 
##  Formula: ~1 | MZP %in% ID
##         (Intercept)   Residual
## StdDev:     0.64688 0.00028059
## 
## Fixed effects: SWLS ~ MZP * CLASS 
##               Value Std.Error   DF t-value p-value
## (Intercept)  5.6023  0.027837 6164 201.253  0.0000
## MZP2        -0.0004  0.039591 6164  -0.011  0.9915
## MZP3         0.0286  0.042086 6164   0.680  0.4962
## MZP4         0.0420  0.041974 6164   1.001  0.3168
## MZP5         0.0437  0.044704 6164   0.978  0.3279
## MZP6         0.0056  0.047362 6164   0.118  0.9062
## CLASS2       0.6234  0.041416 1897  15.053  0.0000
## CLASS3      -0.9325  0.042403 1897 -21.992  0.0000
## CLASS4      -1.5845  0.048457 1897 -32.699  0.0000
## CLASS5      -2.9760  0.066199 1897 -44.956  0.0000
## MZP2:CLASS2  0.0183  0.059038 6164   0.309  0.7570
## MZP3:CLASS2  0.0192  0.063729 6164   0.301  0.7634
## MZP4:CLASS2  0.0741  0.063010 6164   1.175  0.2399
## MZP5:CLASS2 -0.0300  0.066347 6164  -0.452  0.6516
## MZP6:CLASS2  0.0897  0.070297 6164   1.276  0.2020
## MZP2:CLASS3  0.0611  0.060712 6164   1.007  0.3142
## MZP3:CLASS3  0.2476  0.064724 6164   3.825  0.0001
## MZP4:CLASS3  0.1964  0.064797 6164   3.032  0.0024
## MZP5:CLASS3 -0.0286  0.068426 6164  -0.418  0.6758
## MZP6:CLASS3  0.0971  0.072853 6164   1.332  0.1828
## MZP2:CLASS4 -0.2794  0.069386 6164  -4.027  0.0001
## MZP3:CLASS4 -0.4285  0.073733 6164  -5.811  0.0000
## MZP4:CLASS4 -0.5130  0.074259 6164  -6.908  0.0000
## MZP5:CLASS4 -0.2702  0.078350 6164  -3.448  0.0006
## MZP6:CLASS4 -0.4021  0.083729 6164  -4.802  0.0000
## MZP2:CLASS5 -0.3171  0.096111 6164  -3.300  0.0010
## MZP3:CLASS5 -0.3321  0.103322 6164  -3.215  0.0013
## MZP4:CLASS5 -0.2654  0.106928 6164  -2.482  0.0131
## MZP5:CLASS5 -0.1942  0.113228 6164  -1.715  0.0864
## MZP6:CLASS5 -0.1876  0.123939 6164  -1.513  0.1303
##  Correlation: 
##             (Intr) MZP2   MZP3   MZP4   MZP5   MZP6   CLASS2 CLASS3 CLASS4
## MZP2        -0.703                                                        
## MZP3        -0.661  0.465                                                 
## MZP4        -0.663  0.466  0.439                                          
## MZP5        -0.623  0.438  0.412  0.413                                   
## MZP6        -0.588  0.413  0.389  0.390  0.366                            
## CLASS2      -0.672  0.473  0.445  0.446  0.419  0.395                     
## CLASS3      -0.656  0.462  0.434  0.435  0.409  0.386  0.441              
## CLASS4      -0.574  0.404  0.380  0.381  0.358  0.338  0.386  0.377       
## CLASS5      -0.421  0.296  0.278  0.279  0.262  0.247  0.283  0.276  0.242
## MZP2:CLASS2  0.472 -0.671 -0.312 -0.313 -0.294 -0.277 -0.702 -0.310 -0.271
## MZP3:CLASS2  0.437 -0.307 -0.660 -0.290 -0.272 -0.257 -0.650 -0.287 -0.251
## MZP4:CLASS2  0.442 -0.311 -0.292 -0.666 -0.275 -0.260 -0.657 -0.290 -0.254
## MZP5:CLASS2  0.420 -0.295 -0.278 -0.278 -0.674 -0.247 -0.624 -0.275 -0.241
## MZP6:CLASS2  0.396 -0.278 -0.262 -0.263 -0.247 -0.674 -0.589 -0.260 -0.227
## MZP2:CLASS3  0.459 -0.652 -0.303 -0.304 -0.286 -0.269 -0.308 -0.698 -0.263
## MZP3:CLASS3  0.430 -0.302 -0.650 -0.285 -0.268 -0.253 -0.289 -0.655 -0.247
## MZP4:CLASS3  0.430 -0.302 -0.284 -0.648 -0.268 -0.253 -0.289 -0.654 -0.247
## MZP5:CLASS3  0.407 -0.286 -0.269 -0.270 -0.653 -0.239 -0.273 -0.620 -0.234
## MZP6:CLASS3  0.382 -0.269 -0.253 -0.253 -0.238 -0.650 -0.257 -0.582 -0.220
## MZP2:CLASS4  0.401 -0.571 -0.265 -0.266 -0.250 -0.236 -0.270 -0.263 -0.698
## MZP3:CLASS4  0.378 -0.265 -0.571 -0.250 -0.235 -0.222 -0.254 -0.248 -0.657
## MZP4:CLASS4  0.375 -0.264 -0.248 -0.565 -0.233 -0.220 -0.252 -0.246 -0.653
## MZP5:CLASS4  0.355 -0.250 -0.235 -0.236 -0.571 -0.209 -0.239 -0.233 -0.618
## MZP6:CLASS4  0.332 -0.234 -0.220 -0.220 -0.207 -0.566 -0.223 -0.218 -0.579
## MZP2:CLASS5  0.290 -0.412 -0.192 -0.192 -0.180 -0.170 -0.195 -0.190 -0.166
## MZP3:CLASS5  0.269 -0.189 -0.407 -0.179 -0.168 -0.158 -0.181 -0.177 -0.155
## MZP4:CLASS5  0.260 -0.183 -0.172 -0.393 -0.162 -0.153 -0.175 -0.171 -0.150
## MZP5:CLASS5  0.246 -0.173 -0.163 -0.163 -0.395 -0.145 -0.165 -0.161 -0.141
## MZP6:CLASS5  0.225 -0.158 -0.149 -0.149 -0.140 -0.382 -0.151 -0.147 -0.129
##             CLASS5 MZP2:CLASS2 MZP3:CLASS2 MZP4:CLASS2 MZP5:CLASS2 MZP6:CLASS2
## MZP2                                                                          
## MZP3                                                                          
## MZP4                                                                          
## MZP5                                                                          
## MZP6                                                                          
## CLASS2                                                                        
## CLASS3                                                                        
## CLASS4                                                                        
## CLASS5                                                                        
## MZP2:CLASS2 -0.198                                                            
## MZP3:CLASS2 -0.184  0.456                                                     
## MZP4:CLASS2 -0.186  0.461       0.427                                         
## MZP5:CLASS2 -0.176  0.438       0.406       0.410                             
## MZP6:CLASS2 -0.167  0.413       0.383       0.387       0.368                 
## MZP2:CLASS3 -0.193  0.437       0.200       0.203       0.192       0.182     
## MZP3:CLASS3 -0.181  0.203       0.429       0.190       0.180       0.170     
## MZP4:CLASS3 -0.181  0.203       0.188       0.432       0.180       0.170     
## MZP5:CLASS3 -0.171  0.192       0.178       0.180       0.440       0.161     
## MZP6:CLASS3 -0.161  0.180       0.167       0.169       0.160       0.438     
## MZP2:CLASS4 -0.169  0.383       0.175       0.177       0.168       0.159     
## MZP3:CLASS4 -0.159  0.178       0.377       0.167       0.158       0.150     
## MZP4:CLASS4 -0.158  0.177       0.164       0.377       0.157       0.148     
## MZP5:CLASS4 -0.149  0.168       0.155       0.157       0.384       0.141     
## MZP6:CLASS4 -0.140  0.157       0.145       0.147       0.139       0.381     
## MZP2:CLASS5 -0.689  0.276       0.127       0.128       0.122       0.115     
## MZP3:CLASS5 -0.641  0.127       0.269       0.119       0.113       0.107     
## MZP4:CLASS5 -0.619  0.123       0.114       0.261       0.109       0.103     
## MZP5:CLASS5 -0.585  0.116       0.107       0.109       0.266       0.097     
## MZP6:CLASS5 -0.534  0.106       0.098       0.099       0.094       0.257     
##             MZP2:CLASS3 MZP3:CLASS3 MZP4:CLASS3 MZP5:CLASS3 MZP6:CLASS3
## MZP2                                                                   
## MZP3                                                                   
## MZP4                                                                   
## MZP5                                                                   
## MZP6                                                                   
## CLASS2                                                                 
## CLASS3                                                                 
## CLASS4                                                                 
## CLASS5                                                                 
## MZP2:CLASS2                                                            
## MZP3:CLASS2                                                            
## MZP4:CLASS2                                                            
## MZP5:CLASS2                                                            
## MZP6:CLASS2                                                            
## MZP2:CLASS3                                                            
## MZP3:CLASS3  0.458                                                     
## MZP4:CLASS3  0.457       0.429                                         
## MZP5:CLASS3  0.433       0.406       0.406                             
## MZP6:CLASS3  0.407       0.381       0.381       0.361                 
## MZP2:CLASS4  0.372       0.173       0.172       0.163       0.153     
## MZP3:CLASS4  0.173       0.371       0.162       0.154       0.144     
## MZP4:CLASS4  0.172       0.161       0.366       0.153       0.143     
## MZP5:CLASS4  0.163       0.153       0.153       0.373       0.136     
## MZP6:CLASS4  0.152       0.143       0.143       0.135       0.368     
## MZP2:CLASS5  0.269       0.125       0.124       0.118       0.111     
## MZP3:CLASS5  0.124       0.265       0.116       0.110       0.103     
## MZP4:CLASS5  0.119       0.112       0.254       0.106       0.099     
## MZP5:CLASS5  0.113       0.106       0.106       0.258       0.094     
## MZP6:CLASS5  0.103       0.097       0.096       0.091       0.248     
##             MZP2:CLASS4 MZP3:CLASS4 MZP4:CLASS4 MZP5:CLASS4 MZP6:CLASS4
## MZP2                                                                   
## MZP3                                                                   
## MZP4                                                                   
## MZP5                                                                   
## MZP6                                                                   
## CLASS2                                                                 
## CLASS3                                                                 
## CLASS4                                                                 
## CLASS5                                                                 
## MZP2:CLASS2                                                            
## MZP3:CLASS2                                                            
## MZP4:CLASS2                                                            
## MZP5:CLASS2                                                            
## MZP6:CLASS2                                                            
## MZP2:CLASS3                                                            
## MZP3:CLASS3                                                            
## MZP4:CLASS3                                                            
## MZP5:CLASS3                                                            
## MZP6:CLASS3                                                            
## MZP2:CLASS4                                                            
## MZP3:CLASS4  0.459                                                     
## MZP4:CLASS4  0.456       0.429                                         
## MZP5:CLASS4  0.432       0.406       0.404                             
## MZP6:CLASS4  0.404       0.380       0.378       0.358                 
## MZP2:CLASS5  0.235       0.109       0.109       0.103       0.096     
## MZP3:CLASS5  0.108       0.232       0.101       0.096       0.090     
## MZP4:CLASS5  0.104       0.098       0.222       0.092       0.087     
## MZP5:CLASS5  0.099       0.093       0.092       0.225       0.082     
## MZP6:CLASS5  0.090       0.085       0.084       0.080       0.216     
##             MZP2:CLASS5 MZP3:CLASS5 MZP4:CLASS5 MZP5:CLASS5
## MZP2                                                       
## MZP3                                                       
## MZP4                                                       
## MZP5                                                       
## MZP6                                                       
## CLASS2                                                     
## CLASS3                                                     
## CLASS4                                                     
## CLASS5                                                     
## MZP2:CLASS2                                                
## MZP3:CLASS2                                                
## MZP4:CLASS2                                                
## MZP5:CLASS2                                                
## MZP6:CLASS2                                                
## MZP2:CLASS3                                                
## MZP3:CLASS3                                                
## MZP4:CLASS3                                                
## MZP5:CLASS3                                                
## MZP6:CLASS3                                                
## MZP2:CLASS4                                                
## MZP3:CLASS4                                                
## MZP4:CLASS4                                                
## MZP5:CLASS4                                                
## MZP6:CLASS4                                                
## MZP2:CLASS5                                                
## MZP3:CLASS5  0.441                                         
## MZP4:CLASS5  0.426       0.397                             
## MZP5:CLASS5  0.403       0.375       0.362                 
## MZP6:CLASS5  0.368       0.342       0.331       0.312     
## 
## Standardized Within-Group Residuals:
##         Min          Q1         Med          Q3         Max 
## -0.00311423 -0.00021525 -0.00000127  0.00025388  0.00199965 
## 
## Number of Observations: 8091
## Number of Groups: 
##          ID MZP %in% ID 
##        1902        8091
```

```
anova(M.SWLS)
```

```
##             numDF denDF F-value p-value
## (Intercept)     1  6164  508572  <.0001
## MZP             5  6164       3  0.0045
## CLASS           4  1897    5488  <.0001
## MZP:CLASS      20  6164       7  <.0001
```

```
## JSAT
# C2
JM6 = data.frame(rep(c(1:nrow(J6)), 6), rep(J6$JSAT_C2, 6), rep(c(1:6), each = nrow(J6)), c(J6$JSAT_1, J6$JSAT_2, J6$JSAT_3, J6$JSAT_4, J6$JSAT_5, J6$JSAT_6))
colnames(JM6) = c("ID", "CLASS", "MZP", "JSAT")
JM6$ID = factor(JM6$ID)
JM6$CLASS = factor(JM6$CLASS)
JM6$MZP = factor(JM6$MZP)
JM6 = na.omit(JM6)
JM6$INT = interaction(JM6$MZP, JM6$CLASS)

M.JSAT = lme(JSAT ~ MZP*CLASS, data = JM6, random = ~1|ID/MZP)
summary(M.JSAT)
```

```
## Linear mixed-effects model fit by REML
##  Data: JM6 
##      AIC    BIC  logLik
##   5505.1 5608.6 -2737.6
## 
## Random effects:
##  Formula: ~1 | ID
##         (Intercept)
## StdDev:     0.15176
## 
##  Formula: ~1 | MZP %in% ID
##         (Intercept) Residual
## StdDev:     0.30328  0.11412
## 
## Fixed effects: JSAT ~ MZP * CLASS 
##                Value Std.Error   DF t-value p-value
## (Intercept)  2.97951  0.012883 5565 231.275  0.0000
## MZP2        -0.06278  0.016464 5565  -3.813  0.0001
## MZP3        -0.08400  0.017216 5565  -4.879  0.0000
## MZP4        -0.10522  0.017407 5565  -6.045  0.0000
## MZP5        -0.08534  0.018305 5565  -4.662  0.0000
## MZP6        -0.07555  0.019699 5565  -3.835  0.0001
## CLASS2       0.52989  0.018956 1767  27.954  0.0000
## MZP2:CLASS2  0.04938  0.024297 5565   2.032  0.0422
## MZP3:CLASS2  0.09118  0.025613 5565   3.560  0.0004
## MZP4:CLASS2  0.08701  0.025750 5565   3.379  0.0007
## MZP5:CLASS2  0.05381  0.027088 5565   1.986  0.0470
## MZP6:CLASS2  0.03703  0.028989 5565   1.277  0.2015
##  Correlation: 
##             (Intr) MZP2   MZP3   MZP4   MZP5   MZP6   CLASS2 MZP2:C MZP3:C
## MZP2        -0.667                                                        
## MZP3        -0.642  0.500                                                 
## MZP4        -0.637  0.495  0.485                                          
## MZP5        -0.607  0.472  0.462  0.465                                   
## MZP6        -0.563  0.439  0.429  0.431  0.422                            
## CLASS2      -0.680  0.453  0.436  0.433  0.412  0.383                     
## MZP2:CLASS2  0.452 -0.678 -0.339 -0.335 -0.320 -0.297 -0.663              
## MZP3:CLASS2  0.432 -0.336 -0.672 -0.326 -0.310 -0.288 -0.633  0.492       
## MZP4:CLASS2  0.431 -0.335 -0.328 -0.676 -0.314 -0.291 -0.632  0.489  0.477
## MZP5:CLASS2  0.410 -0.319 -0.312 -0.314 -0.676 -0.285 -0.600  0.466  0.453
## MZP6:CLASS2  0.382 -0.298 -0.292 -0.293 -0.287 -0.680 -0.562  0.436  0.424
##             MZP4:C MZP5:C
## MZP2                     
## MZP3                     
## MZP4                     
## MZP5                     
## MZP6                     
## CLASS2                   
## MZP2:CLASS2              
## MZP3:CLASS2              
## MZP4:CLASS2              
## MZP5:CLASS2  0.458       
## MZP6:CLASS2  0.429  0.418
## 
## Standardized Within-Group Residuals:
##       Min        Q1       Med        Q3       Max 
## -2.104621 -0.194247  0.020872  0.213286  1.370103 
## 
## Number of Observations: 7344
## Number of Groups: 
##          ID MZP %in% ID 
##        1769        7344
```

```
anova(M.JSAT)
```

```
##             numDF denDF F-value p-value
## (Intercept)     1  5565  356542  <.0001
## MZP             5  5565       8  <.0001
## CLASS           1  1767    2963  <.0001
## MZP:CLASS       5  5565       3  0.0044
```

```
MI.JSAT = lme(JSAT ~ INT, data = JM6, random = ~1|ID/MZP)
MCP.JSAT = glht(MI.JSAT, linfct = mcp(INT = CH2))
summary(MCP.JSAT)
```

```
## 
##   Simultaneous Tests for General Linear Hypotheses
## 
## Multiple Comparisons of Means: User-defined Contrasts
## 
## 
## Fit: lme.formula(fixed = JSAT ~ INT, data = JM6, random = ~1 | ID/MZP)
## 
## Linear Hypotheses:
##             Estimate Std. Error z value Pr(>|z|)    
## G1|G2 == 0   0.58296    0.01085   53.73   <0.001 ***
## G1: M1 == 0 -0.06882    0.01118   -6.16   <0.001 ***
## G1: M2 == 0 -0.00604    0.01079   -0.56     1.00    
## G1: M3 == 0  0.01518    0.01139    1.33     0.91    
## G1: M4 == 0  0.03641    0.01151    3.16     0.02 *  
## G1: M5 == 0  0.01653    0.01234    1.34     0.90    
## G1: M6 == 0  0.00674    0.01370    0.49     1.00    
## G2: M1 == 0 -0.01575    0.01209   -1.30     0.92    
## G2: M2 == 0 -0.00235    0.01183   -0.20     1.00    
## G2: M3 == 0 -0.02293    0.01273   -1.80     0.59    
## G2: M4 == 0  0.00246    0.01267    0.19     1.00    
## G2: M5 == 0  0.01579    0.01359    1.16     0.96    
## G2: M6 == 0  0.02277    0.01484    1.53     0.80    
## ---
## Signif. codes:  0 '***' 0.001 '**' 0.01 '*' 0.05 '.' 0.1 ' ' 1
## (Adjusted p values reported -- single-step method)
```

```
# C3
JM6 = data.frame(rep(c(1:nrow(J6)), 6), rep(J6$JSAT_C3, 6), rep(c(1:6), each = nrow(J6)), c(J6$JSAT_1, J6$JSAT_2, J6$JSAT_3, J6$JSAT_4, J6$JSAT_5, J6$JSAT_6))
colnames(JM6) = c("ID", "CLASS", "MZP", "JSAT")
JM6$ID = factor(JM6$ID)
JM6$CLASS = factor(JM6$CLASS)
JM6$MZP = factor(JM6$MZP)
JM6 = na.omit(JM6)
JM6$INT = interaction(JM6$MZP, JM6$CLASS)

M.JSAT = lme(JSAT ~ MZP*CLASS, data = JM6, random = ~1|ID/MZP)
summary(M.JSAT)
```

```
## Linear mixed-effects model fit by REML
##  Data: JM6 
##      AIC    BIC  logLik
##   4492.4 4637.3 -2225.2
## 
## Random effects:
##  Formula: ~1 | ID
##         (Intercept)
## StdDev:    0.057132
## 
##  Formula: ~1 | MZP %in% ID
##         (Intercept) Residual
## StdDev:     0.30402  0.10207
## 
## Fixed effects: JSAT ~ MZP * CLASS 
##                Value Std.Error   DF t-value p-value
## (Intercept)  3.15204  0.012882 5560 244.688  0.0000
## MZP2        -0.05637  0.017458 5560  -3.229  0.0012
## MZP3        -0.07387  0.018137 5560  -4.073  0.0000
## MZP4        -0.08692  0.018297 5560  -4.751  0.0000
## MZP5        -0.08267  0.018932 5560  -4.367  0.0000
## MZP6        -0.08007  0.020281 5560  -3.948  0.0001
## CLASS2       0.40794  0.019316 1766  21.120  0.0000
## CLASS3      -0.40018  0.023922 1766 -16.728  0.0000
## MZP2:CLASS2  0.06295  0.026541 5560   2.372  0.0177
## MZP3:CLASS2  0.11064  0.027808 5560   3.979  0.0001
## MZP4:CLASS2  0.09393  0.027855 5560   3.372  0.0008
## MZP5:CLASS2  0.07163  0.029134 5560   2.459  0.0140
## MZP6:CLASS2  0.07254  0.031082 5560   2.334  0.0196
## MZP2:CLASS3 -0.04166  0.033047 5560  -1.261  0.2075
## MZP3:CLASS3 -0.06269  0.034540 5560  -1.815  0.0696
## MZP4:CLASS3 -0.09026  0.034861 5560  -2.589  0.0096
## MZP5:CLASS3 -0.04949  0.037305 5560  -1.327  0.1847
## MZP6:CLASS3 -0.03883  0.039783 5560  -0.976  0.3291
##  Correlation: 
##             (Intr) MZP2   MZP3   MZP4   MZP5   MZP6   CLASS2 CLASS3 MZP2:CLASS2
## MZP2        -0.719                                                             
## MZP3        -0.694  0.511                                                      
## MZP4        -0.688  0.507  0.490                                               
## MZP5        -0.665  0.490  0.474  0.471                                        
## MZP6        -0.621  0.458  0.442  0.440  0.427                                 
## CLASS2      -0.667  0.480  0.463  0.459  0.443  0.414                          
## CLASS3      -0.538  0.387  0.374  0.370  0.358  0.334  0.359                   
## MZP2:CLASS2  0.473 -0.658 -0.336 -0.333 -0.322 -0.301 -0.709 -0.255            
## MZP3:CLASS2  0.452 -0.333 -0.652 -0.320 -0.309 -0.289 -0.678 -0.244  0.493     
## MZP4:CLASS2  0.452 -0.333 -0.322 -0.657 -0.309 -0.289 -0.677 -0.243  0.492     
## MZP5:CLASS2  0.432 -0.318 -0.308 -0.306 -0.650 -0.278 -0.647 -0.233  0.470     
## MZP6:CLASS2  0.405 -0.299 -0.289 -0.287 -0.279 -0.652 -0.607 -0.218  0.441     
## MZP2:CLASS3  0.380 -0.528 -0.270 -0.268 -0.259 -0.242 -0.253 -0.706  0.348     
## MZP3:CLASS3  0.364 -0.268 -0.525 -0.257 -0.249 -0.232 -0.243 -0.676  0.177     
## MZP4:CLASS3  0.361 -0.266 -0.257 -0.525 -0.247 -0.231 -0.241 -0.670  0.175     
## MZP5:CLASS3  0.337 -0.249 -0.240 -0.239 -0.507 -0.217 -0.225 -0.626  0.164     
## MZP6:CLASS3  0.316 -0.233 -0.226 -0.224 -0.218 -0.510 -0.211 -0.587  0.153     
##             MZP3:CLASS2 MZP4:CLASS2 MZP5:CLASS2 MZP6:CLASS2 MZP2:CLASS3
## MZP2                                                                   
## MZP3                                                                   
## MZP4                                                                   
## MZP5                                                                   
## MZP6                                                                   
## CLASS2                                                                 
## CLASS3                                                                 
## MZP2:CLASS2                                                            
## MZP3:CLASS2                                                            
## MZP4:CLASS2  0.472                                                     
## MZP5:CLASS2  0.451       0.452                                         
## MZP6:CLASS2  0.423       0.424       0.408                             
## MZP2:CLASS3  0.176       0.176       0.168       0.158                 
## MZP3:CLASS3  0.342       0.169       0.162       0.152       0.489     
## MZP4:CLASS3  0.168       0.345       0.161       0.151       0.484     
## MZP5:CLASS3  0.157       0.157       0.330       0.142       0.453     
## MZP6:CLASS3  0.147       0.147       0.142       0.333       0.425     
##             MZP3:CLASS3 MZP4:CLASS3 MZP5:CLASS3
## MZP2                                           
## MZP3                                           
## MZP4                                           
## MZP5                                           
## MZP6                                           
## CLASS2                                         
## CLASS3                                         
## MZP2:CLASS2                                    
## MZP3:CLASS2                                    
## MZP4:CLASS2                                    
## MZP5:CLASS2                                    
## MZP6:CLASS2                                    
## MZP2:CLASS3                                    
## MZP3:CLASS3                                    
## MZP4:CLASS3  0.466                             
## MZP5:CLASS3  0.436       0.433                 
## MZP6:CLASS3  0.409       0.406       0.383     
## 
## Standardized Within-Group Residuals:
##        Min         Q1        Med         Q3        Max 
## -2.0228256 -0.2050053  0.0057865  0.2294921  1.4195682 
## 
## Number of Observations: 7344
## Number of Groups: 
##          ID MZP %in% ID 
##        1769        7344
```

```
anova(M.JSAT)
```

```
##             numDF denDF F-value p-value
## (Intercept)     1  5560  629058  <.0001
## MZP             5  5560       8  <.0001
## CLASS           2  1766    3256  <.0001
## MZP:CLASS      10  5560       4  <.0001
```

```
MI.JSAT = lme(JSAT ~ INT, data = JM6, random = ~1|ID/MZP)
MCP.JSAT = glht(MI.JSAT, linfct = mcp(INT = CH3))


# C4
JM6 = data.frame(rep(c(1:nrow(J6)), 6), rep(J6$JSAT_C4, 6), rep(c(1:6), each = nrow(J6)), c(J6$JSAT_1, J6$JSAT_2, J6$JSAT_3, J6$JSAT_4, J6$JSAT_5, J6$JSAT_6))
colnames(JM6) = c("ID", "CLASS", "MZP", "JSAT")
JM6$ID = factor(JM6$ID)
JM6$CLASS = factor(JM6$CLASS)
JM6$MZP = factor(JM6$MZP)
JM6 = na.omit(JM6)
JM6$INT = interaction(JM6$MZP, JM6$CLASS)


M.JSAT = lme(JSAT ~ MZP*CLASS, data = JM6, random = ~1|ID/MZP)
summary(M.JSAT)
```

```
## Linear mixed-effects model fit by REML
##  Data: JM6 
##      AIC    BIC  logLik
##   3752.5 3938.7 -1849.2
## 
## Random effects:
##  Formula: ~1 | ID
##         (Intercept)
## StdDev:  2.3541e-05
## 
##  Formula: ~1 | MZP %in% ID
##         (Intercept)   Residual
## StdDev:     0.30868 1.4456e-05
## 
## Fixed effects: JSAT ~ MZP * CLASS 
##                Value Std.Error   DF t-value p-value
## (Intercept)  2.92749  0.014016 5555 208.860  0.0000
## MZP2         0.05758  0.019423 5555   2.965  0.0030
## MZP3         0.03073  0.020023 5555   1.535  0.1249
## MZP4         0.02467  0.020158 5555   1.224  0.2210
## MZP5         0.02709  0.020849 5555   1.299  0.1938
## MZP6         0.00053  0.022359 5555   0.024  0.9809
## CLASS2       0.47812  0.020124 1765  23.759  0.0000
## CLASS3       0.67642  0.022067 1765  30.653  0.0000
## CLASS4      -0.13172  0.029453 1765  -4.472  0.0000
## MZP2:CLASS2 -0.21885  0.027877 5555  -7.851  0.0000
## MZP3:CLASS2 -0.16271  0.028957 5555  -5.619  0.0000
## MZP4:CLASS2 -0.18794  0.029069 5555  -6.465  0.0000
## MZP5:CLASS2 -0.19166  0.030230 5555  -6.340  0.0000
## MZP6:CLASS2 -0.11291  0.032365 5555  -3.489  0.0005
## MZP2:CLASS3  0.02803  0.030871 5555   0.908  0.3640
## MZP3:CLASS3  0.06504  0.032316 5555   2.013  0.0442
## MZP4:CLASS3  0.04660  0.032320 5555   1.442  0.1494
## MZP5:CLASS3  0.04480  0.033810 5555   1.325  0.1852
## MZP6:CLASS3  0.00976  0.035943 5555   0.272  0.7859
## MZP2:CLASS4 -0.33884  0.041188 5555  -8.227  0.0000
## MZP3:CLASS4 -0.37245  0.043274 5555  -8.607  0.0000
## MZP4:CLASS4 -0.46829  0.044268 5555 -10.579  0.0000
## MZP5:CLASS4 -0.45798  0.048746 5555  -9.395  0.0000
## MZP6:CLASS4 -0.28759  0.051350 5555  -5.601  0.0000
##  Correlation: 
##             (Intr) MZP2   MZP3   MZP4   MZP5   MZP6   CLASS2 CLASS3 CLASS4
## MZP2        -0.722                                                        
## MZP3        -0.700  0.505                                                 
## MZP4        -0.695  0.502  0.487                                          
## MZP5        -0.672  0.485  0.471  0.467                                   
## MZP6        -0.627  0.452  0.439  0.436  0.421                            
## CLASS2      -0.697  0.503  0.488  0.484  0.468  0.437                     
## CLASS3      -0.635  0.458  0.445  0.442  0.427  0.398  0.442              
## CLASS4      -0.476  0.343  0.333  0.331  0.320  0.298  0.331  0.302       
## MZP2:CLASS2  0.503 -0.697 -0.352 -0.350 -0.338 -0.315 -0.722 -0.319 -0.239
## MZP3:CLASS2  0.484 -0.349 -0.691 -0.337 -0.325 -0.303 -0.695 -0.307 -0.230
## MZP4:CLASS2  0.482 -0.348 -0.338 -0.693 -0.324 -0.302 -0.692 -0.306 -0.229
## MZP5:CLASS2  0.464 -0.335 -0.325 -0.322 -0.690 -0.291 -0.666 -0.295 -0.221
## MZP6:CLASS2  0.433 -0.313 -0.303 -0.301 -0.291 -0.691 -0.622 -0.275 -0.206
## MZP2:CLASS3  0.454 -0.629 -0.318 -0.316 -0.305 -0.285 -0.316 -0.715 -0.216
## MZP3:CLASS3  0.434 -0.313 -0.620 -0.302 -0.292 -0.272 -0.302 -0.683 -0.206
## MZP4:CLASS3  0.434 -0.313 -0.304 -0.624 -0.292 -0.272 -0.302 -0.683 -0.206
## MZP5:CLASS3  0.415 -0.299 -0.290 -0.288 -0.617 -0.260 -0.289 -0.653 -0.197
## MZP6:CLASS3  0.390 -0.281 -0.273 -0.271 -0.262 -0.622 -0.272 -0.614 -0.186
## MZP2:CLASS4  0.340 -0.472 -0.238 -0.237 -0.229 -0.213 -0.237 -0.216 -0.715
## MZP3:CLASS4  0.324 -0.234 -0.463 -0.225 -0.218 -0.203 -0.226 -0.206 -0.681
## MZP4:CLASS4  0.317 -0.228 -0.222 -0.455 -0.213 -0.198 -0.221 -0.201 -0.665
## MZP5:CLASS4  0.288 -0.208 -0.201 -0.200 -0.428 -0.180 -0.200 -0.183 -0.604
## MZP6:CLASS4  0.273 -0.197 -0.191 -0.190 -0.184 -0.435 -0.190 -0.173 -0.574
##             MZP2:CLASS2 MZP3:CLASS2 MZP4:CLASS2 MZP5:CLASS2 MZP6:CLASS2
## MZP2                                                                   
## MZP3                                                                   
## MZP4                                                                   
## MZP5                                                                   
## MZP6                                                                   
## CLASS2                                                                 
## CLASS3                                                                 
## CLASS4                                                                 
## MZP2:CLASS2                                                            
## MZP3:CLASS2  0.502                                                     
## MZP4:CLASS2  0.500       0.481                                         
## MZP5:CLASS2  0.481       0.463       0.461                             
## MZP6:CLASS2  0.449       0.432       0.430       0.414                 
## MZP2:CLASS3  0.438       0.220       0.219       0.211       0.197     
## MZP3:CLASS3  0.218       0.428       0.209       0.201       0.188     
## MZP4:CLASS3  0.218       0.210       0.433       0.201       0.188     
## MZP5:CLASS3  0.208       0.201       0.200       0.425       0.180     
## MZP6:CLASS3  0.196       0.189       0.188       0.181       0.430     
## MZP2:CLASS4  0.329       0.165       0.164       0.158       0.147     
## MZP3:CLASS4  0.163       0.320       0.156       0.150       0.140     
## MZP4:CLASS4  0.159       0.153       0.316       0.147       0.137     
## MZP5:CLASS4  0.145       0.139       0.139       0.295       0.125     
## MZP6:CLASS4  0.137       0.132       0.132       0.127       0.301     
##             MZP2:CLASS3 MZP3:CLASS3 MZP4:CLASS3 MZP5:CLASS3 MZP6:CLASS3
## MZP2                                                                   
## MZP3                                                                   
## MZP4                                                                   
## MZP5                                                                   
## MZP6                                                                   
## CLASS2                                                                 
## CLASS3                                                                 
## CLASS4                                                                 
## MZP2:CLASS2                                                            
## MZP3:CLASS2                                                            
## MZP4:CLASS2                                                            
## MZP5:CLASS2                                                            
## MZP6:CLASS2                                                            
## MZP2:CLASS3                                                            
## MZP3:CLASS3  0.488                                                     
## MZP4:CLASS3  0.488       0.466                                         
## MZP5:CLASS3  0.467       0.446       0.446                             
## MZP6:CLASS3  0.439       0.419       0.419       0.401                 
## MZP2:CLASS4  0.297       0.148       0.148       0.141       0.133     
## MZP3:CLASS4  0.147       0.287       0.140       0.134       0.126     
## MZP4:CLASS4  0.144       0.137       0.284       0.131       0.123     
## MZP5:CLASS4  0.131       0.125       0.125       0.264       0.112     
## MZP6:CLASS4  0.124       0.118       0.118       0.113       0.271     
##             MZP2:CLASS4 MZP3:CLASS4 MZP4:CLASS4 MZP5:CLASS4
## MZP2                                                       
## MZP3                                                       
## MZP4                                                       
## MZP5                                                       
## MZP6                                                       
## CLASS2                                                     
## CLASS3                                                     
## CLASS4                                                     
## MZP2:CLASS2                                                
## MZP3:CLASS2                                                
## MZP4:CLASS2                                                
## MZP5:CLASS2                                                
## MZP6:CLASS2                                                
## MZP2:CLASS3                                                
## MZP3:CLASS3                                                
## MZP4:CLASS3                                                
## MZP5:CLASS3                                                
## MZP6:CLASS3                                                
## MZP2:CLASS4                                                
## MZP3:CLASS4  0.487                                         
## MZP4:CLASS4  0.476       0.453                             
## MZP5:CLASS4  0.432       0.411       0.402                 
## MZP6:CLASS4  0.410       0.390       0.382       0.347     
## 
## Standardized Within-Group Residuals:
##         Min          Q1         Med          Q3         Max 
## -2.9242e-04 -2.6671e-05  6.0828e-06  3.2175e-05  2.5000e-04 
## 
## Number of Observations: 7344
## Number of Groups: 
##          ID MZP %in% ID 
##        1769        7344
```

```
anova(M.JSAT)
```

```
##             numDF denDF F-value p-value
## (Intercept)     1  5555  779396  <.0001
## MZP             5  5555       9  <.0001
## CLASS           3  1765    2884  <.0001
## MZP:CLASS      15  5555      17  <.0001
```

```
# C5
JM6 = data.frame(rep(c(1:nrow(J6)), 6), rep(J6$JSAT_C5, 6), rep(c(1:6), each = nrow(J6)), c(J6$JSAT_1, J6$JSAT_2, J6$JSAT_3, J6$JSAT_4, J6$JSAT_5, J6$JSAT_6))
colnames(JM6) = c("ID", "CLASS", "MZP", "JSAT")
JM6$ID = factor(JM6$ID)
JM6$CLASS = factor(JM6$CLASS)
JM6$MZP = factor(JM6$MZP)
JM6 = na.omit(JM6)
JM6$INT = interaction(JM6$MZP, JM6$CLASS)

M.JSAT = lme(JSAT ~ MZP*CLASS, data = JM6, random = ~1|ID/MZP)
summary(M.JSAT)
```

```
## Linear mixed-effects model fit by REML
##  Data: JM6 
##      AIC    BIC  logLik
##   3180.7 3408.3 -1557.3
## 
## Random effects:
##  Formula: ~1 | ID
##         (Intercept)
## StdDev:  0.00010454
## 
##  Formula: ~1 | MZP %in% ID
##         (Intercept)  Residual
## StdDev:     0.29609 0.0010412
## 
## Fixed effects: JSAT ~ MZP * CLASS 
##                Value Std.Error   DF t-value p-value
## (Intercept)  2.91199  0.014695 5550 198.165  0.0000
## MZP2        -0.00517  0.020376 5550  -0.254  0.7998
## MZP3         0.00371  0.020899 5550   0.178  0.8589
## MZP4         0.04620  0.021035 5550   2.196  0.0281
## MZP5         0.06289  0.021784 5550   2.887  0.0039
## MZP6        -0.00985  0.023383 5550  -0.421  0.6736
## CLASS2       0.61681  0.022521 1764  27.388  0.0000
## CLASS3       0.78298  0.022881 1764  34.220  0.0000
## CLASS4       0.17382  0.022929 1764   7.581  0.0000
## CLASS5      -0.10542  0.029255 1764  -3.603  0.0003
## MZP2:CLASS2 -0.20211  0.031005 5550  -6.519  0.0000
## MZP3:CLASS2 -0.34107  0.032003 5550 -10.657  0.0000
## MZP4:CLASS2 -0.49756  0.032220 5550 -15.442  0.0000
## MZP5:CLASS2 -0.57887  0.033677 5550 -17.189  0.0000
## MZP6:CLASS2 -0.40148  0.036082 5550 -11.127  0.0000
## MZP2:CLASS3  0.02226  0.031969 5550   0.696  0.4863
## MZP3:CLASS3  0.01671  0.033405 5550   0.500  0.6169
## MZP4:CLASS3 -0.07657  0.033352 5550  -2.296  0.0217
## MZP5:CLASS3 -0.10059  0.034813 5550  -2.889  0.0039
## MZP6:CLASS3 -0.06855  0.037056 5550  -1.850  0.0644
## MZP2:CLASS4  0.13076  0.032084 5550   4.076  0.0000
## MZP3:CLASS4  0.29439  0.033606 5550   8.760  0.0000
## MZP4:CLASS4  0.29303  0.033691 5550   8.698  0.0000
## MZP5:CLASS4  0.30689  0.034874 5550   8.800  0.0000
## MZP6:CLASS4  0.37980  0.037254 5550  10.195  0.0000
## MZP2:CLASS5 -0.27209  0.040950 5550  -6.644  0.0000
## MZP3:CLASS5 -0.35571  0.043041 5550  -8.264  0.0000
## MZP4:CLASS5 -0.53193  0.043879 5550 -12.123  0.0000
## MZP5:CLASS5 -0.54505  0.048469 5550 -11.245  0.0000
## MZP6:CLASS5 -0.29833  0.050997 5550  -5.850  0.0000
##  Correlation: 
##             (Intr) MZP2   MZP3   MZP4   MZP5   MZP6   CLASS2 CLASS3 CLASS4
## MZP2        -0.721                                                        
## MZP3        -0.703  0.507                                                 
## MZP4        -0.699  0.504  0.491                                          
## MZP5        -0.675  0.486  0.474  0.471                                   
## MZP6        -0.628  0.453  0.442  0.439  0.424                            
## CLASS2      -0.652  0.471  0.459  0.456  0.440  0.410                     
## CLASS3      -0.642  0.463  0.452  0.449  0.433  0.404  0.419              
## CLASS4      -0.641  0.462  0.451  0.448  0.432  0.403  0.418  0.412       
## CLASS5      -0.502  0.362  0.353  0.351  0.339  0.316  0.328  0.323  0.322
## MZP2:CLASS2  0.474 -0.657 -0.333 -0.331 -0.320 -0.298 -0.726 -0.304 -0.304
## MZP3:CLASS2  0.459 -0.331 -0.653 -0.321 -0.310 -0.289 -0.704 -0.295 -0.294
## MZP4:CLASS2  0.456 -0.329 -0.321 -0.653 -0.308 -0.287 -0.699 -0.293 -0.292
## MZP5:CLASS2  0.436 -0.315 -0.307 -0.305 -0.647 -0.274 -0.669 -0.280 -0.280
## MZP6:CLASS2  0.407 -0.294 -0.286 -0.285 -0.275 -0.648 -0.624 -0.262 -0.261
## MZP2:CLASS3  0.460 -0.637 -0.323 -0.321 -0.310 -0.289 -0.300 -0.716 -0.295
## MZP3:CLASS3  0.440 -0.317 -0.626 -0.307 -0.297 -0.276 -0.287 -0.685 -0.282
## MZP4:CLASS3  0.441 -0.318 -0.310 -0.631 -0.297 -0.277 -0.287 -0.686 -0.282
## MZP5:CLASS3  0.422 -0.304 -0.297 -0.295 -0.626 -0.265 -0.275 -0.657 -0.271
## MZP6:CLASS3  0.397 -0.286 -0.279 -0.277 -0.268 -0.631 -0.259 -0.617 -0.254
## MZP2:CLASS4  0.458 -0.635 -0.322 -0.320 -0.309 -0.288 -0.299 -0.294 -0.715
## MZP3:CLASS4  0.437 -0.315 -0.622 -0.305 -0.295 -0.275 -0.285 -0.281 -0.682
## MZP4:CLASS4  0.436 -0.315 -0.307 -0.624 -0.294 -0.274 -0.285 -0.280 -0.681
## MZP5:CLASS4  0.421 -0.304 -0.296 -0.294 -0.625 -0.265 -0.275 -0.271 -0.657
## MZP6:CLASS4  0.394 -0.284 -0.277 -0.276 -0.266 -0.628 -0.257 -0.253 -0.615
## MZP2:CLASS5  0.359 -0.498 -0.252 -0.251 -0.242 -0.226 -0.234 -0.230 -0.230
## MZP3:CLASS5  0.341 -0.246 -0.486 -0.239 -0.230 -0.215 -0.223 -0.219 -0.219
## MZP4:CLASS5  0.335 -0.242 -0.235 -0.479 -0.226 -0.210 -0.219 -0.215 -0.215
## MZP5:CLASS5  0.303 -0.219 -0.213 -0.212 -0.449 -0.191 -0.198 -0.195 -0.194
## MZP6:CLASS5  0.288 -0.208 -0.203 -0.201 -0.194 -0.459 -0.188 -0.185 -0.185
##             CLASS5 MZP2:CLASS2 MZP3:CLASS2 MZP4:CLASS2 MZP5:CLASS2 MZP6:CLASS2
## MZP2                                                                          
## MZP3                                                                          
## MZP4                                                                          
## MZP5                                                                          
## MZP6                                                                          
## CLASS2                                                                        
## CLASS3                                                                        
## CLASS4                                                                        
## CLASS5                                                                        
## MZP2:CLASS2 -0.238                                                            
## MZP3:CLASS2 -0.231  0.511                                                     
## MZP4:CLASS2 -0.229  0.508       0.492                                         
## MZP5:CLASS2 -0.219  0.486       0.471       0.467                             
## MZP6:CLASS2 -0.205  0.453       0.439       0.436       0.417                 
## MZP2:CLASS3 -0.231  0.419       0.211       0.210       0.201       0.187     
## MZP3:CLASS3 -0.221  0.208       0.409       0.201       0.192       0.179     
## MZP4:CLASS3 -0.221  0.209       0.202       0.412       0.192       0.179     
## MZP5:CLASS3 -0.212  0.200       0.194       0.193       0.405       0.172     
## MZP6:CLASS3 -0.199  0.188       0.182       0.181       0.173       0.409     
## MZP2:CLASS4 -0.230  0.417       0.210       0.209       0.200       0.187     
## MZP3:CLASS4 -0.220  0.207       0.406       0.199       0.191       0.178     
## MZP4:CLASS4 -0.219  0.207       0.200       0.408       0.190       0.178     
## MZP5:CLASS4 -0.212  0.200       0.193       0.192       0.404       0.172     
## MZP6:CLASS4 -0.198  0.187       0.181       0.180       0.172       0.407     
## MZP2:CLASS5 -0.714  0.327       0.165       0.164       0.157       0.146     
## MZP3:CLASS5 -0.680  0.162       0.317       0.156       0.149       0.139     
## MZP4:CLASS5 -0.667  0.159       0.154       0.313       0.146       0.136     
## MZP5:CLASS5 -0.604  0.144       0.139       0.138       0.291       0.123     
## MZP6:CLASS5 -0.574  0.137       0.132       0.131       0.126       0.297     
##             MZP2:CLASS3 MZP3:CLASS3 MZP4:CLASS3 MZP5:CLASS3 MZP6:CLASS3
## MZP2                                                                   
## MZP3                                                                   
## MZP4                                                                   
## MZP5                                                                   
## MZP6                                                                   
## CLASS2                                                                 
## CLASS3                                                                 
## CLASS4                                                                 
## CLASS5                                                                 
## MZP2:CLASS2                                                            
## MZP3:CLASS2                                                            
## MZP4:CLASS2                                                            
## MZP5:CLASS2                                                            
## MZP6:CLASS2                                                            
## MZP2:CLASS3                                                            
## MZP3:CLASS3  0.490                                                     
## MZP4:CLASS3  0.491       0.470                                         
## MZP5:CLASS3  0.470       0.450       0.451                             
## MZP6:CLASS3  0.442       0.423       0.424       0.406                 
## MZP2:CLASS4  0.405       0.201       0.202       0.193       0.182     
## MZP3:CLASS4  0.201       0.389       0.193       0.185       0.173     
## MZP4:CLASS4  0.200       0.192       0.394       0.184       0.173     
## MZP5:CLASS4  0.194       0.185       0.186       0.391       0.167     
## MZP6:CLASS4  0.181       0.174       0.174       0.166       0.396     
## MZP2:CLASS5  0.317       0.158       0.158       0.151       0.142     
## MZP3:CLASS5  0.157       0.304       0.150       0.144       0.135     
## MZP4:CLASS5  0.154       0.147       0.302       0.141       0.133     
## MZP5:CLASS5  0.139       0.133       0.134       0.281       0.120     
## MZP6:CLASS5  0.132       0.127       0.127       0.122       0.289     
##             MZP2:CLASS4 MZP3:CLASS4 MZP4:CLASS4 MZP5:CLASS4 MZP6:CLASS4
## MZP2                                                                   
## MZP3                                                                   
## MZP4                                                                   
## MZP5                                                                   
## MZP6                                                                   
## CLASS2                                                                 
## CLASS3                                                                 
## CLASS4                                                                 
## CLASS5                                                                 
## MZP2:CLASS2                                                            
## MZP3:CLASS2                                                            
## MZP4:CLASS2                                                            
## MZP5:CLASS2                                                            
## MZP6:CLASS2                                                            
## MZP2:CLASS3                                                            
## MZP3:CLASS3                                                            
## MZP4:CLASS3                                                            
## MZP5:CLASS3                                                            
## MZP6:CLASS3                                                            
## MZP2:CLASS4                                                            
## MZP3:CLASS4  0.488                                                     
## MZP4:CLASS4  0.486       0.464                                         
## MZP5:CLASS4  0.470       0.449       0.447                             
## MZP6:CLASS4  0.440       0.420       0.419       0.405                 
## MZP2:CLASS5  0.316       0.157       0.157       0.151       0.142     
## MZP3:CLASS5  0.156       0.302       0.149       0.144       0.135     
## MZP4:CLASS5  0.153       0.146       0.299       0.141       0.132     
## MZP5:CLASS5  0.139       0.133       0.132       0.281       0.120     
## MZP6:CLASS5  0.132       0.126       0.126       0.121       0.288     
##             MZP2:CLASS5 MZP3:CLASS5 MZP4:CLASS5 MZP5:CLASS5
## MZP2                                                       
## MZP3                                                       
## MZP4                                                       
## MZP5                                                       
## MZP6                                                       
## CLASS2                                                     
## CLASS3                                                     
## CLASS4                                                     
## CLASS5                                                     
## MZP2:CLASS2                                                
## MZP3:CLASS2                                                
## MZP4:CLASS2                                                
## MZP5:CLASS2                                                
## MZP6:CLASS2                                                
## MZP2:CLASS3                                                
## MZP3:CLASS3                                                
## MZP4:CLASS3                                                
## MZP5:CLASS3                                                
## MZP6:CLASS3                                                
## MZP2:CLASS4                                                
## MZP3:CLASS4                                                
## MZP4:CLASS4                                                
## MZP5:CLASS4                                                
## MZP6:CLASS4                                                
## MZP2:CLASS5                                                
## MZP3:CLASS5  0.486                                         
## MZP4:CLASS5  0.476       0.453                             
## MZP5:CLASS5  0.431       0.410       0.402                 
## MZP6:CLASS5  0.410       0.390       0.382       0.346     
## 
## Standardized Within-Group Residuals:
##        Min         Q1        Med         Q3        Max 
## -0.0227072 -0.0019548  0.0001404  0.0022777  0.0138747 
## 
## Number of Observations: 7344
## Number of Groups: 
##          ID MZP %in% ID 
##        1769        7344
```

```
anova(M.JSAT)
```

```
##             numDF denDF F-value p-value
## (Intercept)     1  5550  847086  <.0001
## MZP             5  5550      10  <.0001
## CLASS           4  1764    2304  <.0001
## MZP:CLASS      20  5550      55  <.0001
```
